# Author Correction: Human PC4 supports telomere stability and viability in cells utilizing the alternative lengthening of telomeres mechanism

**DOI:** 10.1038/s44319-025-00408-6

**Published:** 2025-02-27

**Authors:** Sara Salgado, Patricia L Abreu, Beatriz Moleirinho, Daniela S Guedes, Lee Larcombe, Claus M Azzalin

**Affiliations:** 1 GIMM - Gulbenkian Institute for Molecular Medicine, 1649-035 Lisbon, Portugal; 2 Apexomic, Stevenage Bioscience Catalyst, Hertfordshire, SG1 2FX UK; 3 TessellateBio Ltd, Stevenage Bioscience Catalyst, Hertfordshire, SG1 2FX UK; 4 Faculty of Medicine, University of Lisbon, Lisbon, 1649-028 Portugal

## Abstract

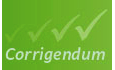

**Correction to:** *EMBO Reports* (2024) 25:5294–5315. 10.1038/s44319-024-00295-3 | Published online 28 October 2024

**One author affiliation is corrected**

The author affiliations for Prof. Claus M. Azzalin are corrected to:

^1^GIMM - Gulbenkian Institute for Molecular Medicine, 1649-035 Lisbon, Portugal

^4^Faculty of Medicine, University of Lisbon, 1649-028 Lisbon, Portugal

