# Supplementary figures and images for: Human PC4 supports telomere stability and viability in cells utilizing the alternative lengthening of telomeres mechanism
Source: EMBO Rep. 2024 Oct 28;25(12):5294–315. doi: 10.1038/s44319-024-00295-3 (PMC11624207; doi:10.1038/s44319-024-00295-3)

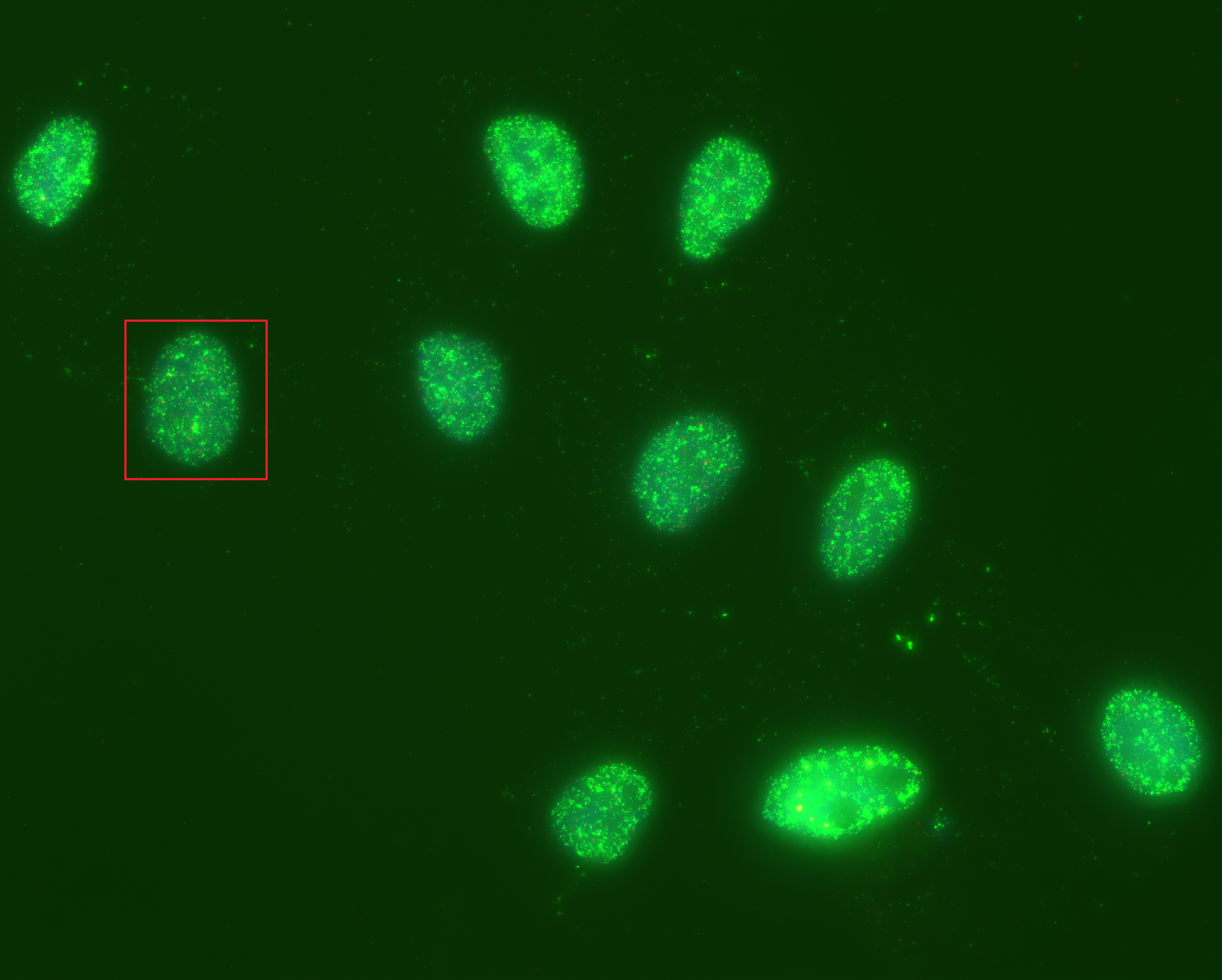

Supplement: Supplementary file 3 — Source data Fig. 2 [file 44319_2024_295_MOESM3_ESM.zip › Figure 2/2A/pS33+TRF2 image - representative nucleus - U2OS siPc2.tif]

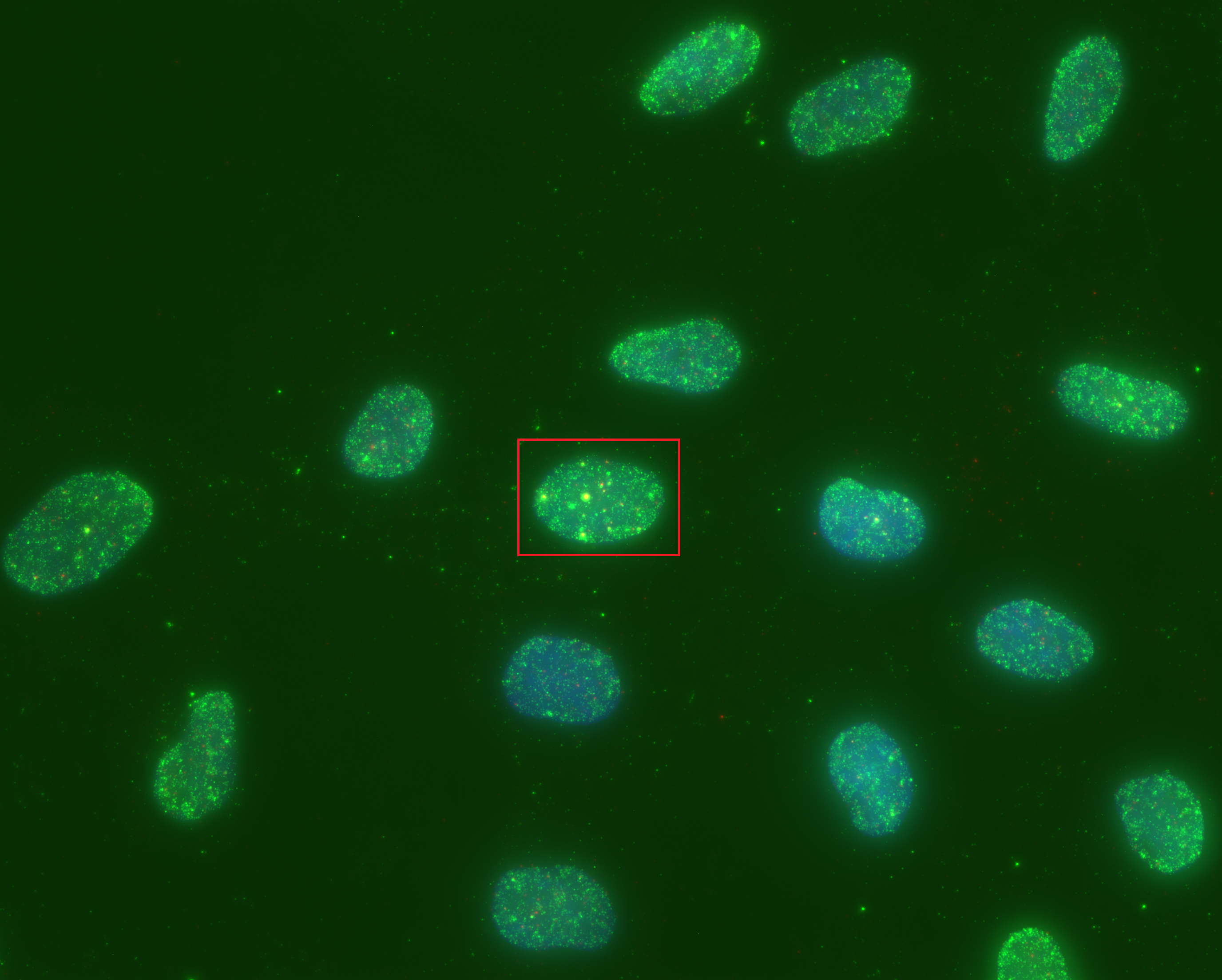

Supplement: Supplementary file 3 — Source data Fig. 2 [file 44319_2024_295_MOESM3_ESM.zip › Figure 2/2A/pS33+TRF2 image - representative nucleus - U2OS siPc1.tif]

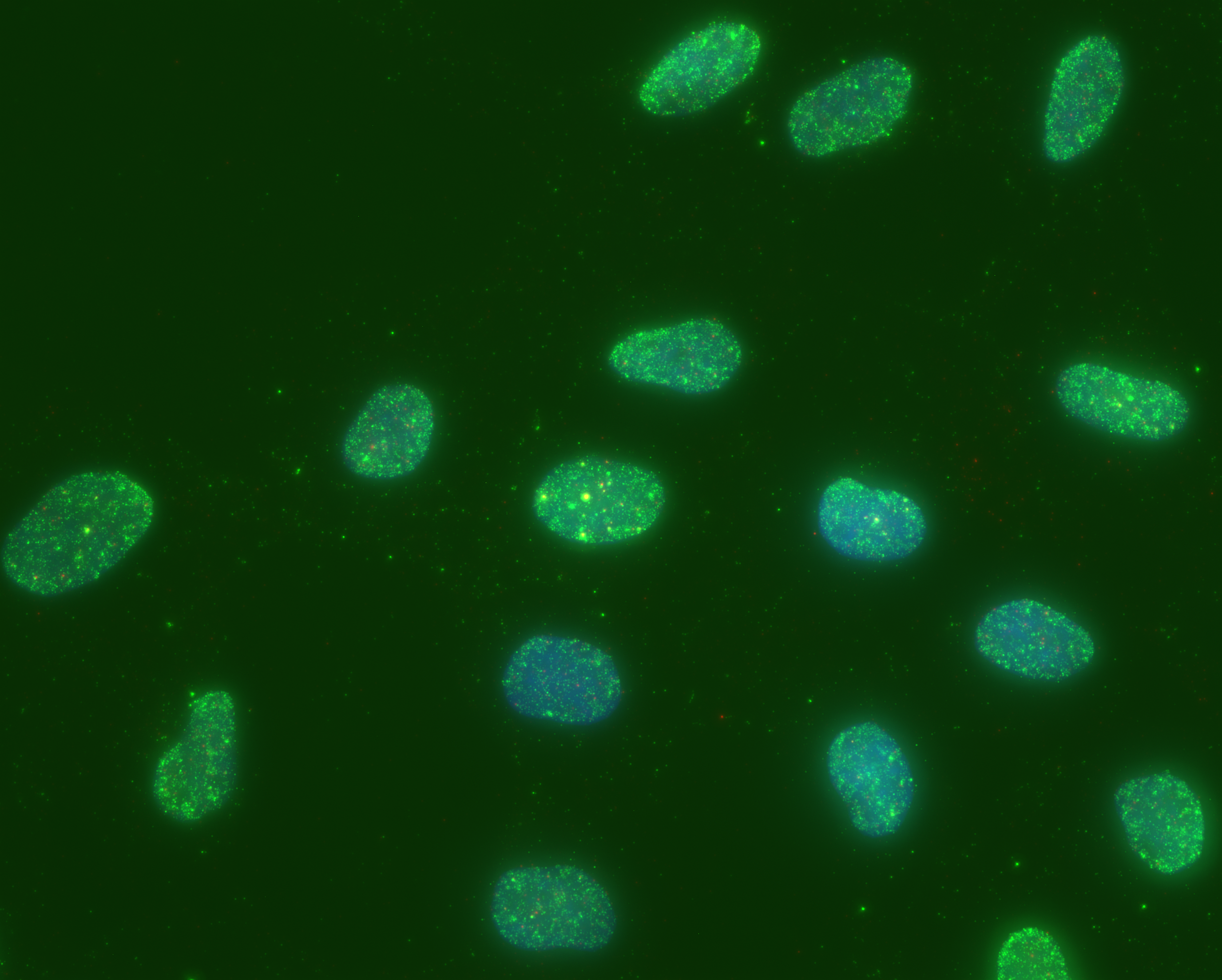

Supplement: Supplementary file 3 — Source data Fig. 2 [file 44319_2024_295_MOESM3_ESM.zip › Figure 2/2A/pS33+TRF2 image - U2OS siPc1.tif]

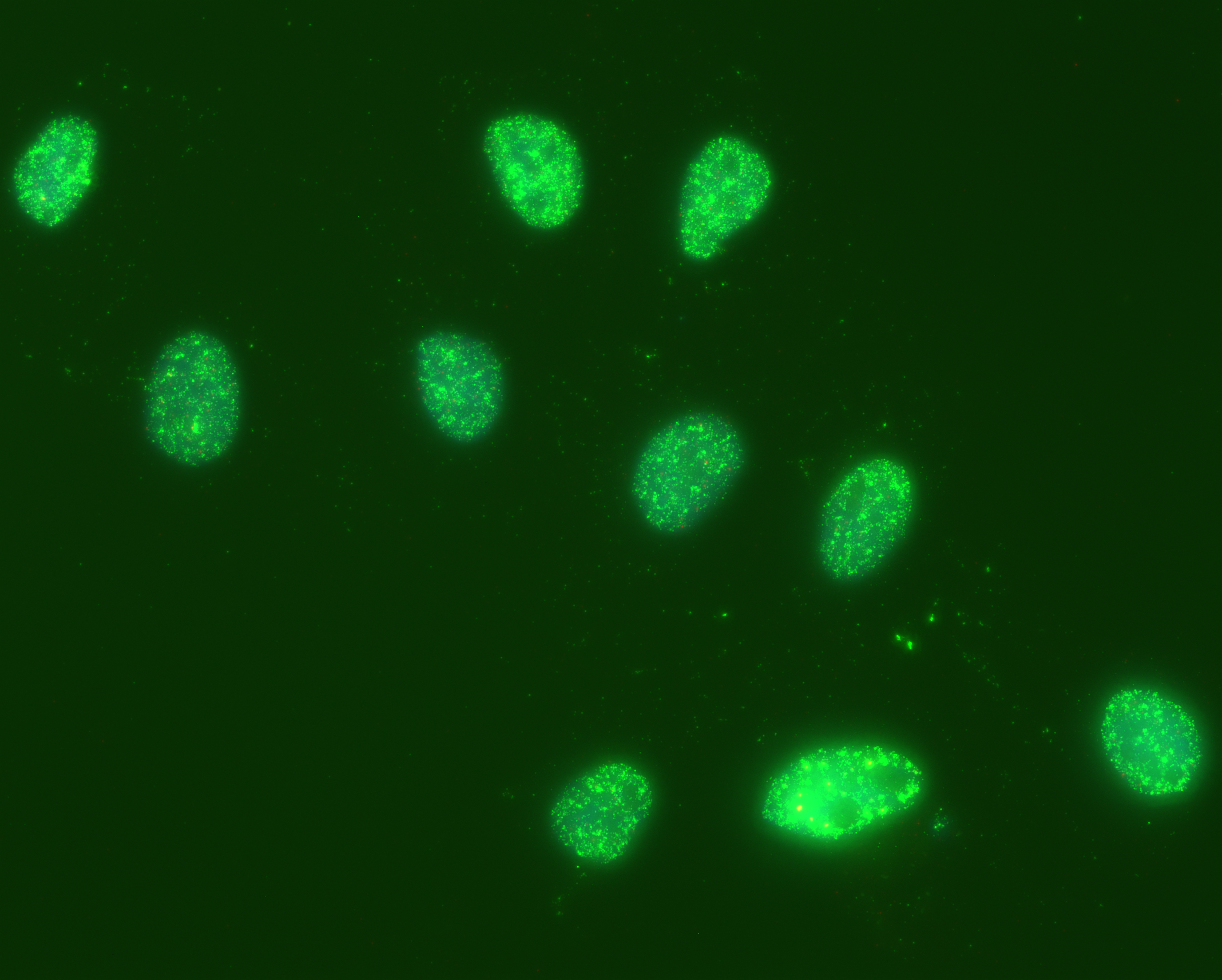

Supplement: Supplementary file 3 — Source data Fig. 2 [file 44319_2024_295_MOESM3_ESM.zip › Figure 2/2A/pS33+TRF2 image - U2OS siPc2.tif]

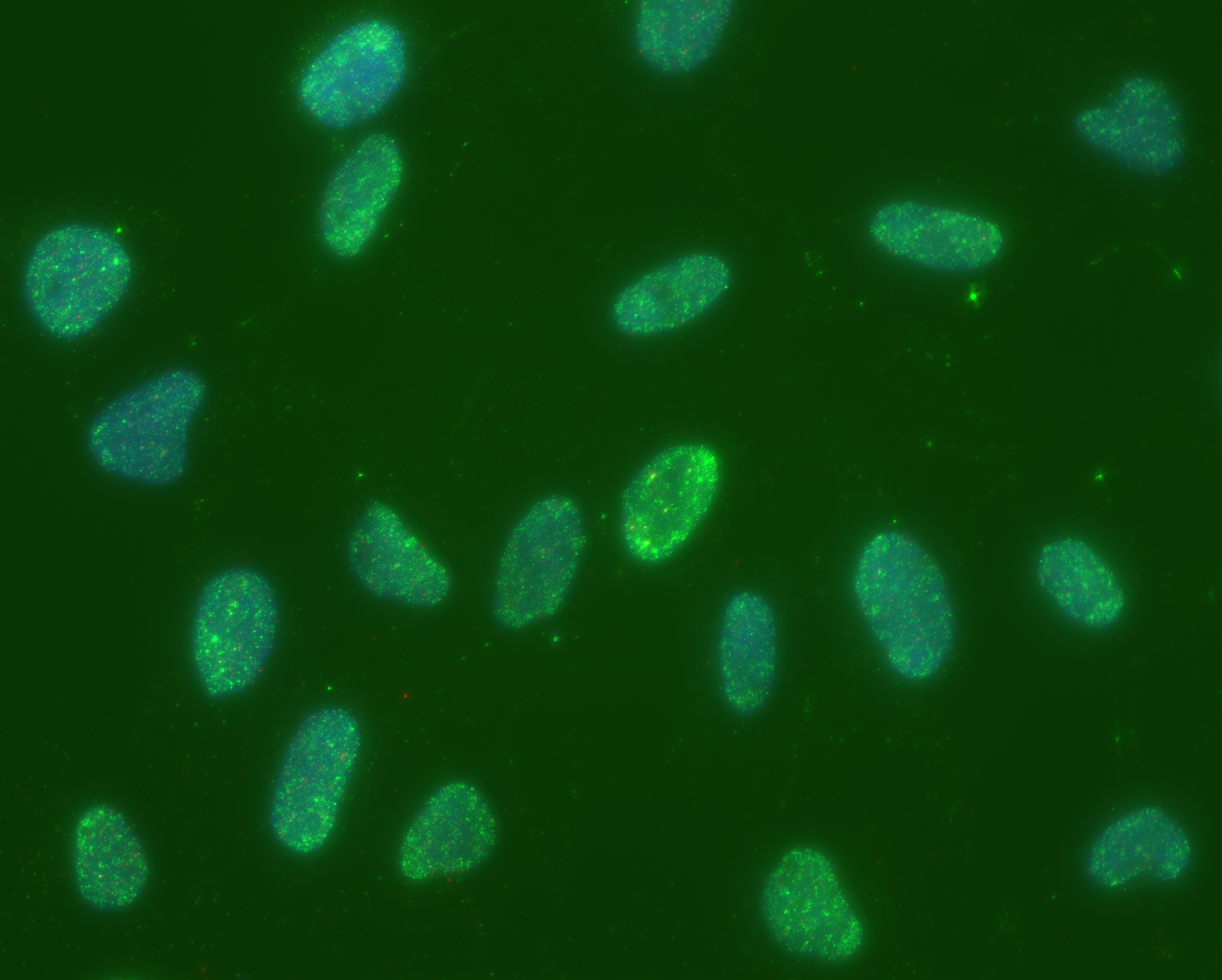

Supplement: Supplementary file 3 — Source data Fig. 2 [file 44319_2024_295_MOESM3_ESM.zip › Figure 2/2A/pS33+TRF2 image - U2OS siCt.tif]

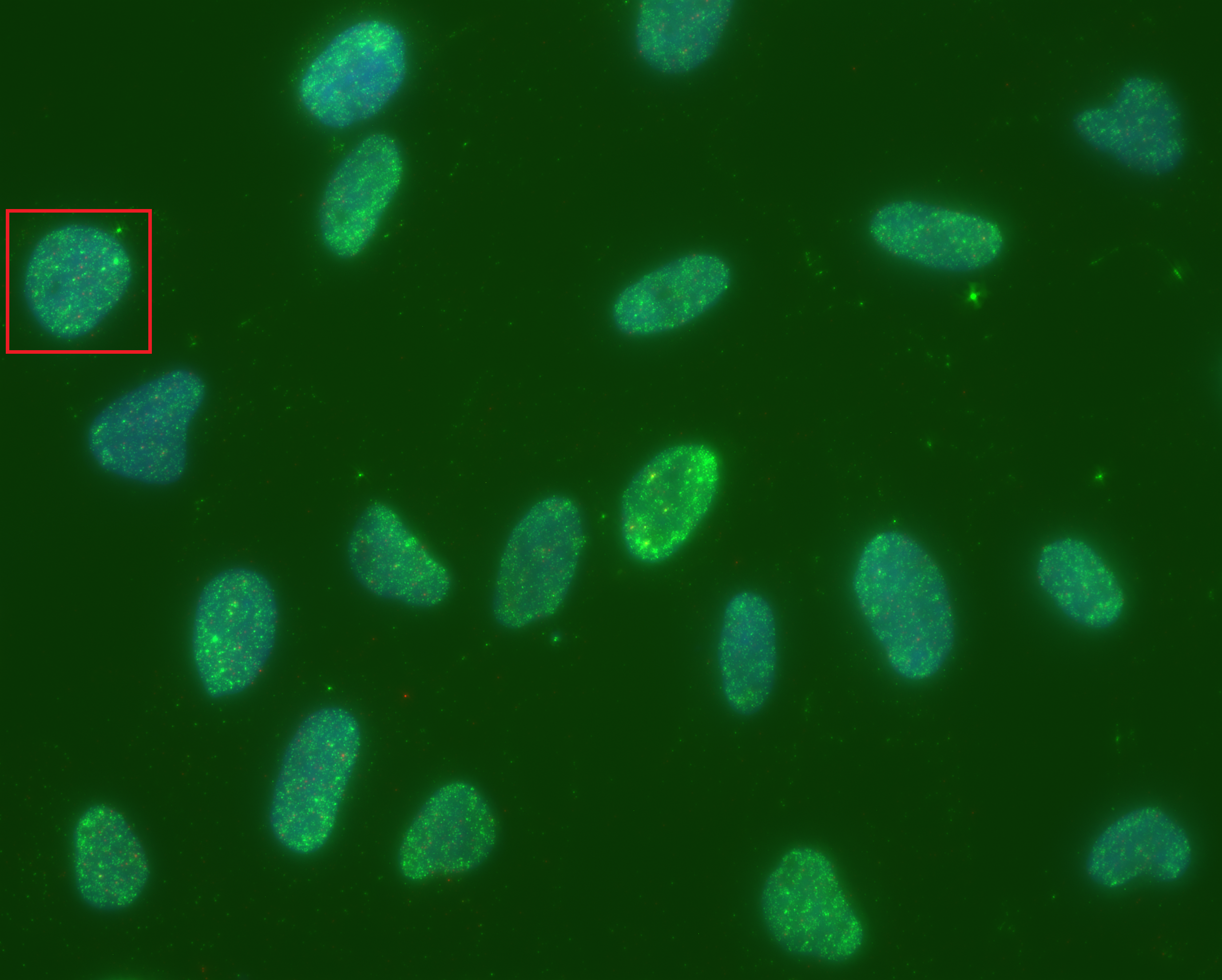

Supplement: Supplementary file 3 — Source data Fig. 2 [file 44319_2024_295_MOESM3_ESM.zip › Figure 2/2A/pS33+TRF2 image - representative nucleus - U2OS siCt.tif]

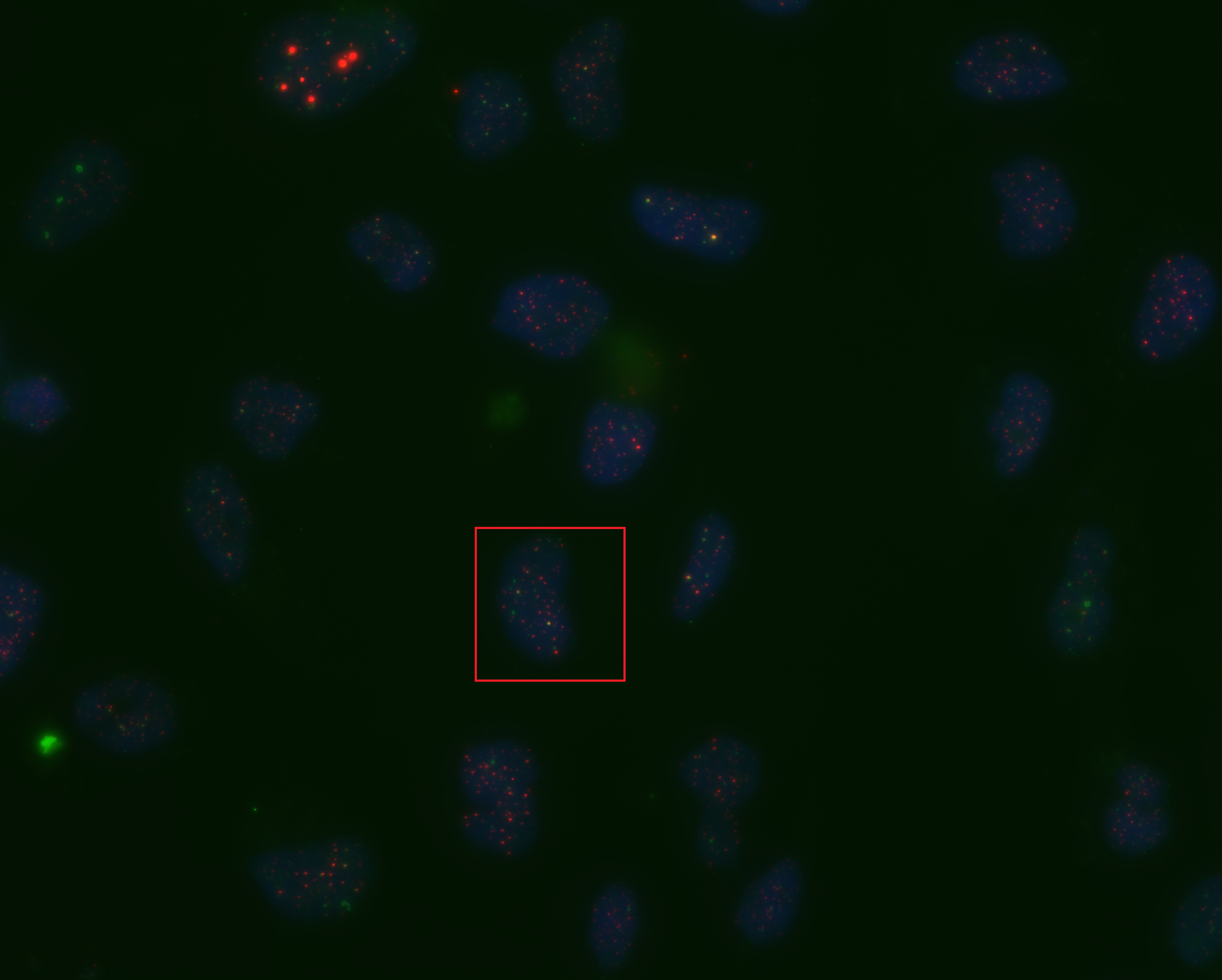

Supplement: Supplementary file 3 — Source data Fig. 2 [file 44319_2024_295_MOESM3_ESM.zip › Figure 2/2C/APBs image - representative nucleus - U2OS siCt.tif]

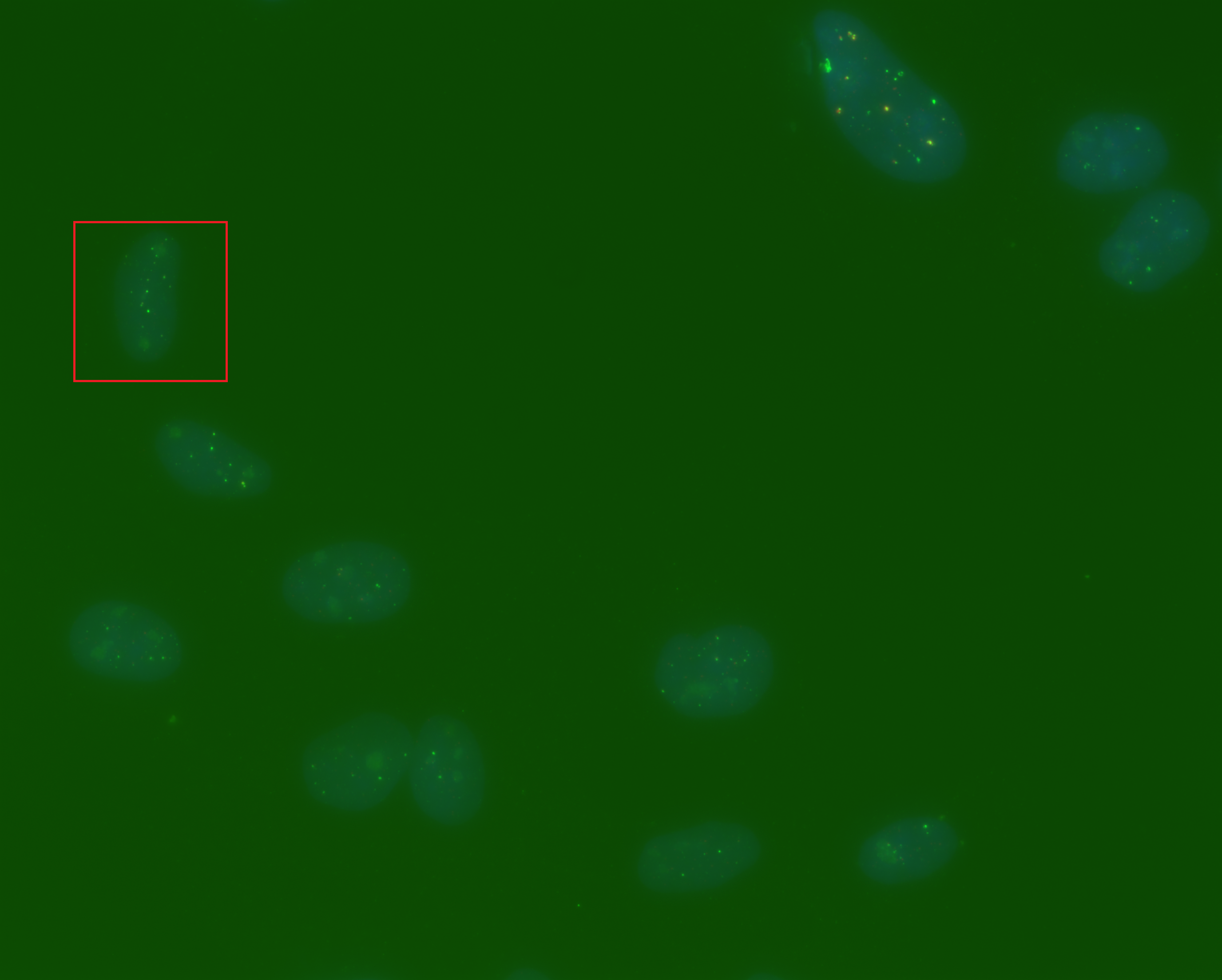

Supplement: Supplementary file 3 — Source data Fig. 2 [file 44319_2024_295_MOESM3_ESM.zip › Figure 2/2C/APBs image - representative nucleus - U2OS siPc2.tif]

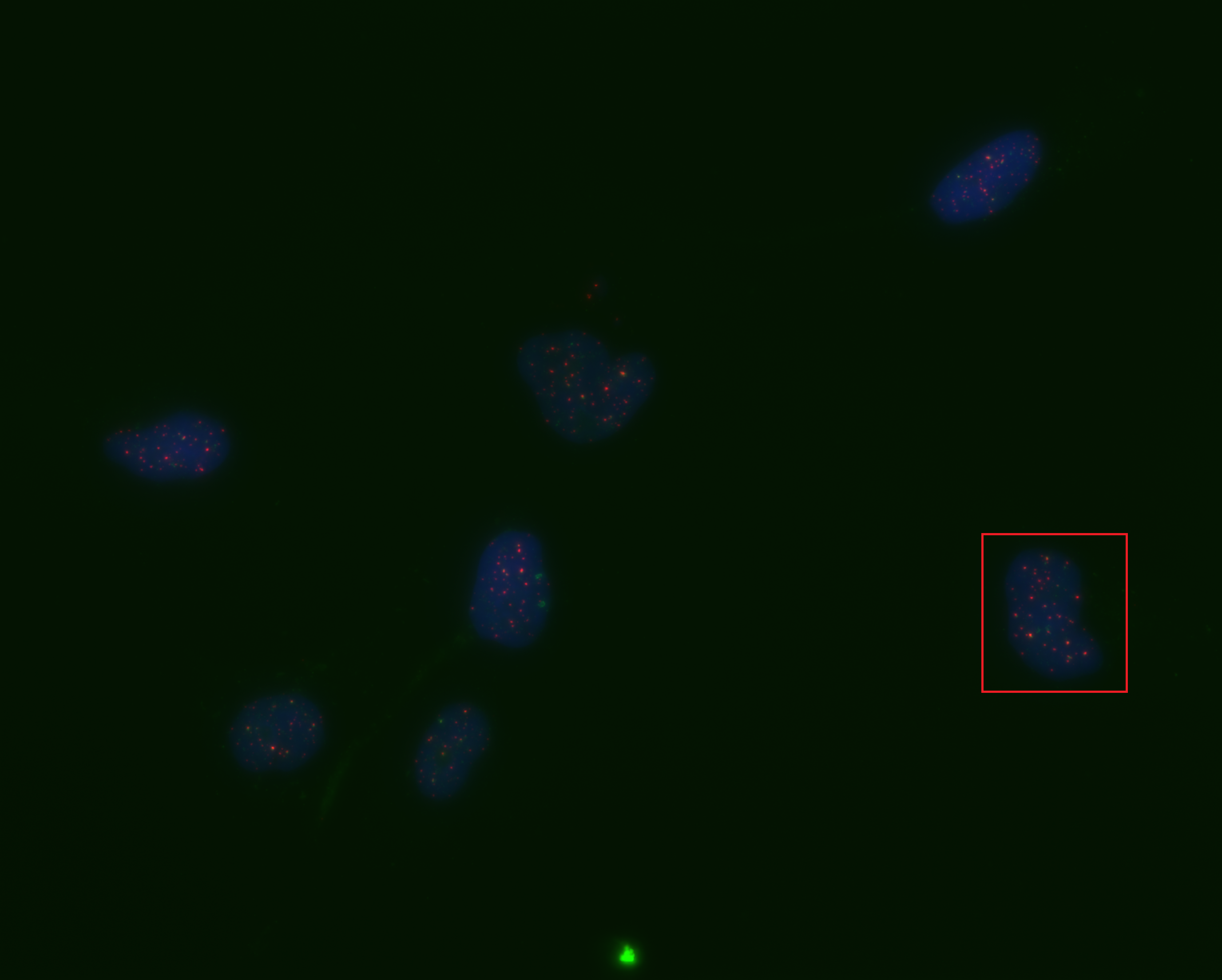

Supplement: Supplementary file 3 — Source data Fig. 2 [file 44319_2024_295_MOESM3_ESM.zip › Figure 2/2C/APBs image - representative nucleus - U2OS siPc1.tif]

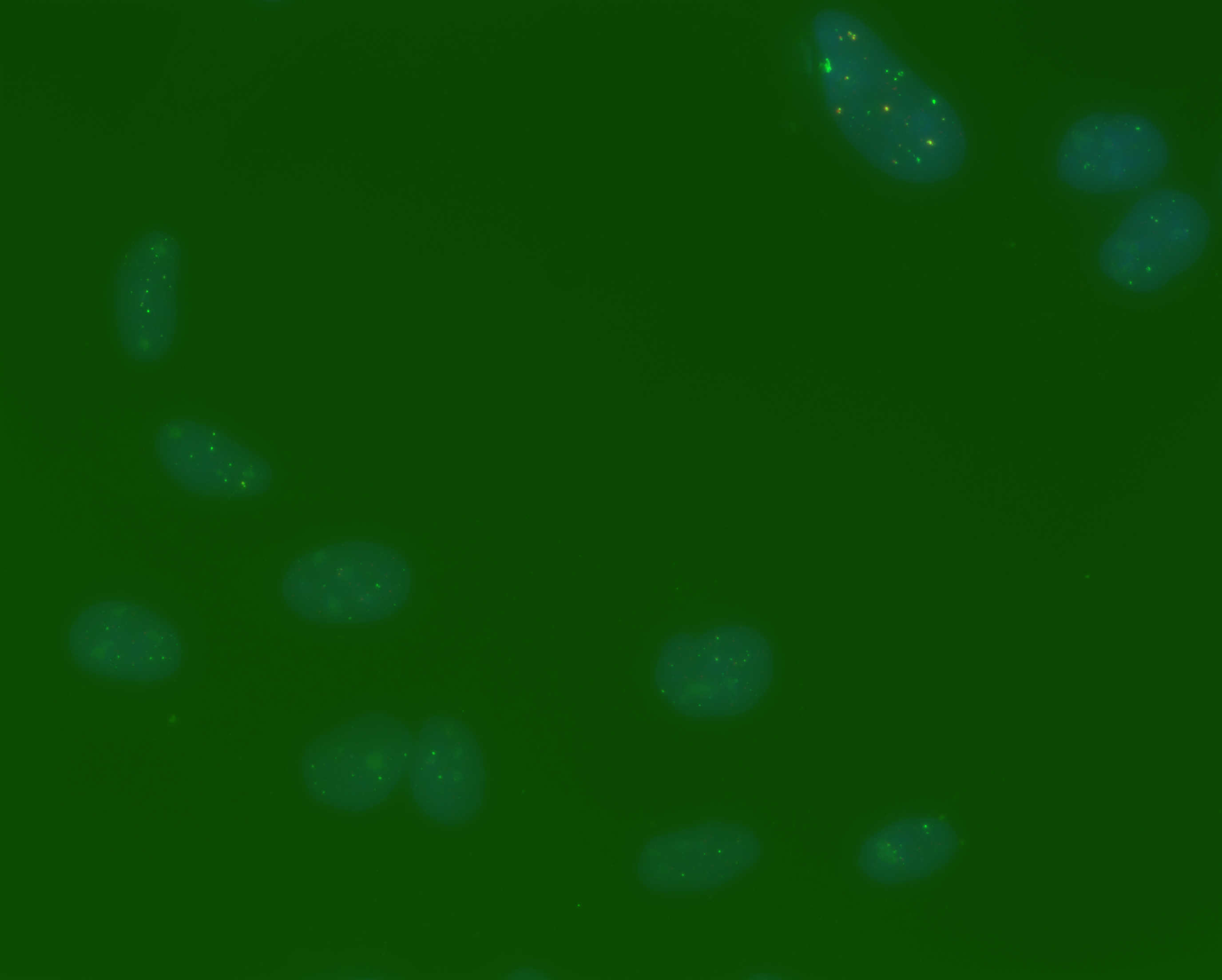

Supplement: Supplementary file 3 — Source data Fig. 2 [file 44319_2024_295_MOESM3_ESM.zip › Figure 2/2C/APBs image - U2OS siPc2.tif]

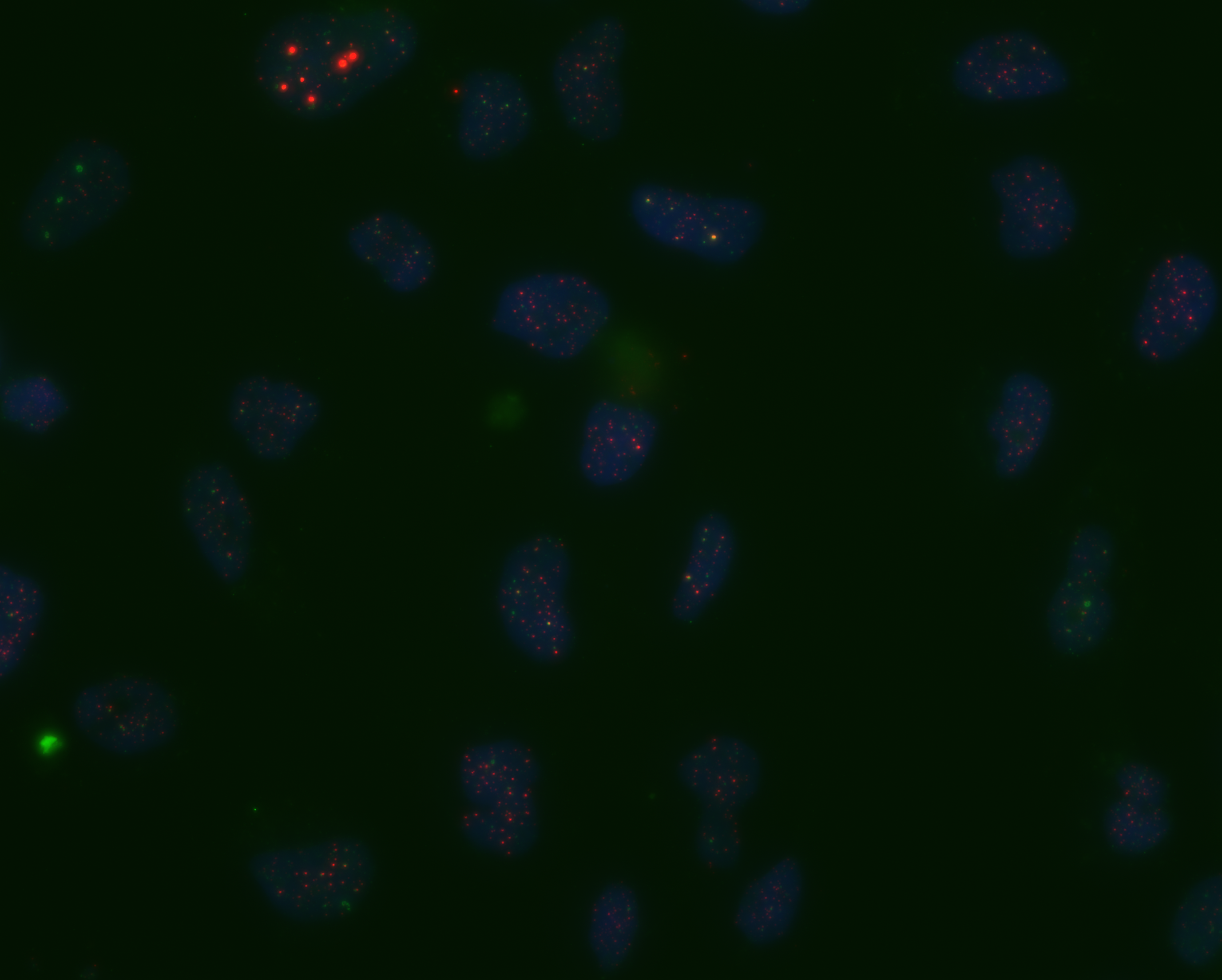

Supplement: Supplementary file 3 — Source data Fig. 2 [file 44319_2024_295_MOESM3_ESM.zip › Figure 2/2C/APBs image - U2OS siCt.tif]

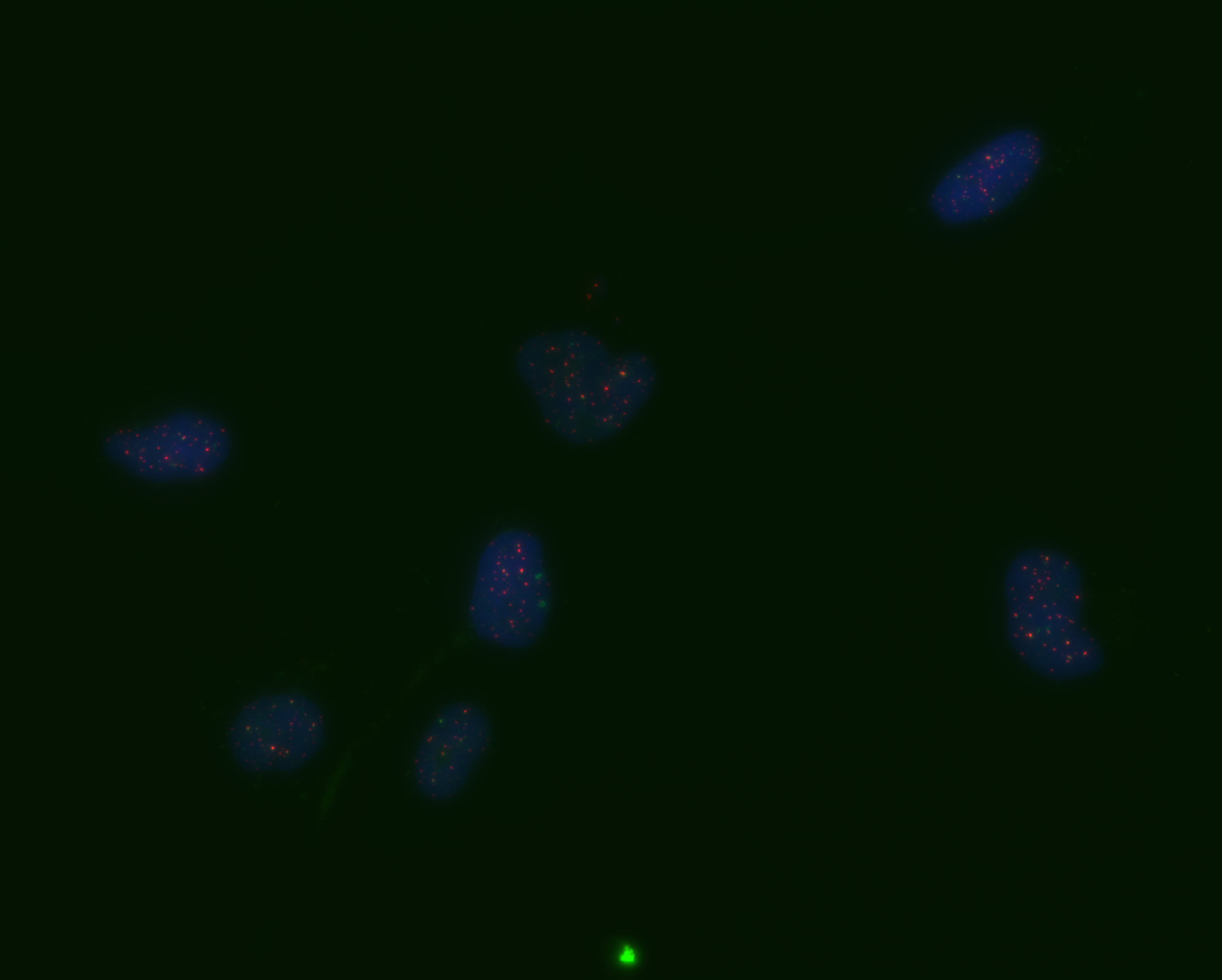

Supplement: Supplementary file 3 — Source data Fig. 2 [file 44319_2024_295_MOESM3_ESM.zip › Figure 2/2C/APBs image - U2OS siPc1.tif]

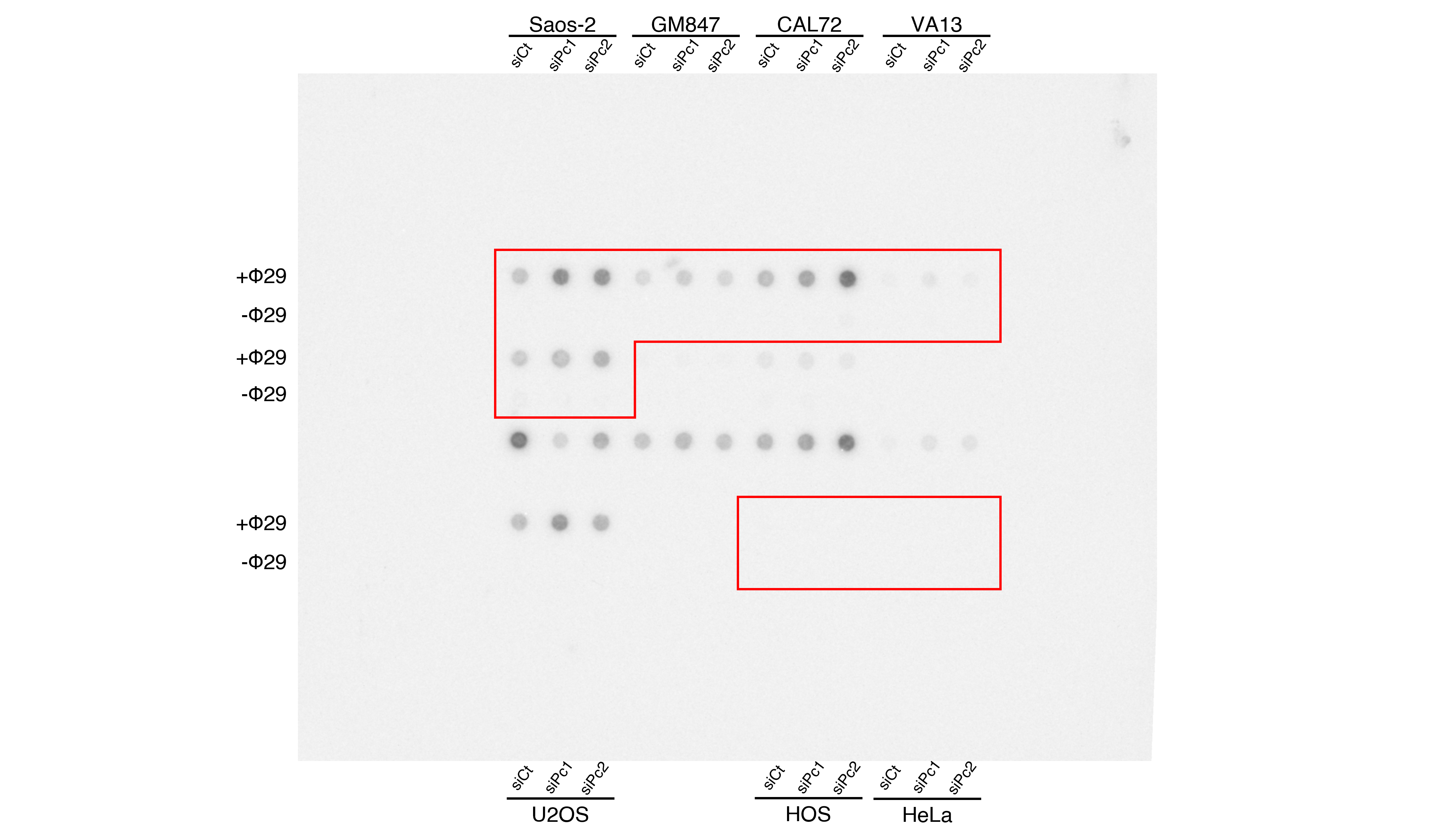

Supplement: Supplementary file 3 — Source data Fig. 2 [file 44319_2024_295_MOESM3_ESM.zip › Figure 2/2D/BlotImage-C-Circles.tif]

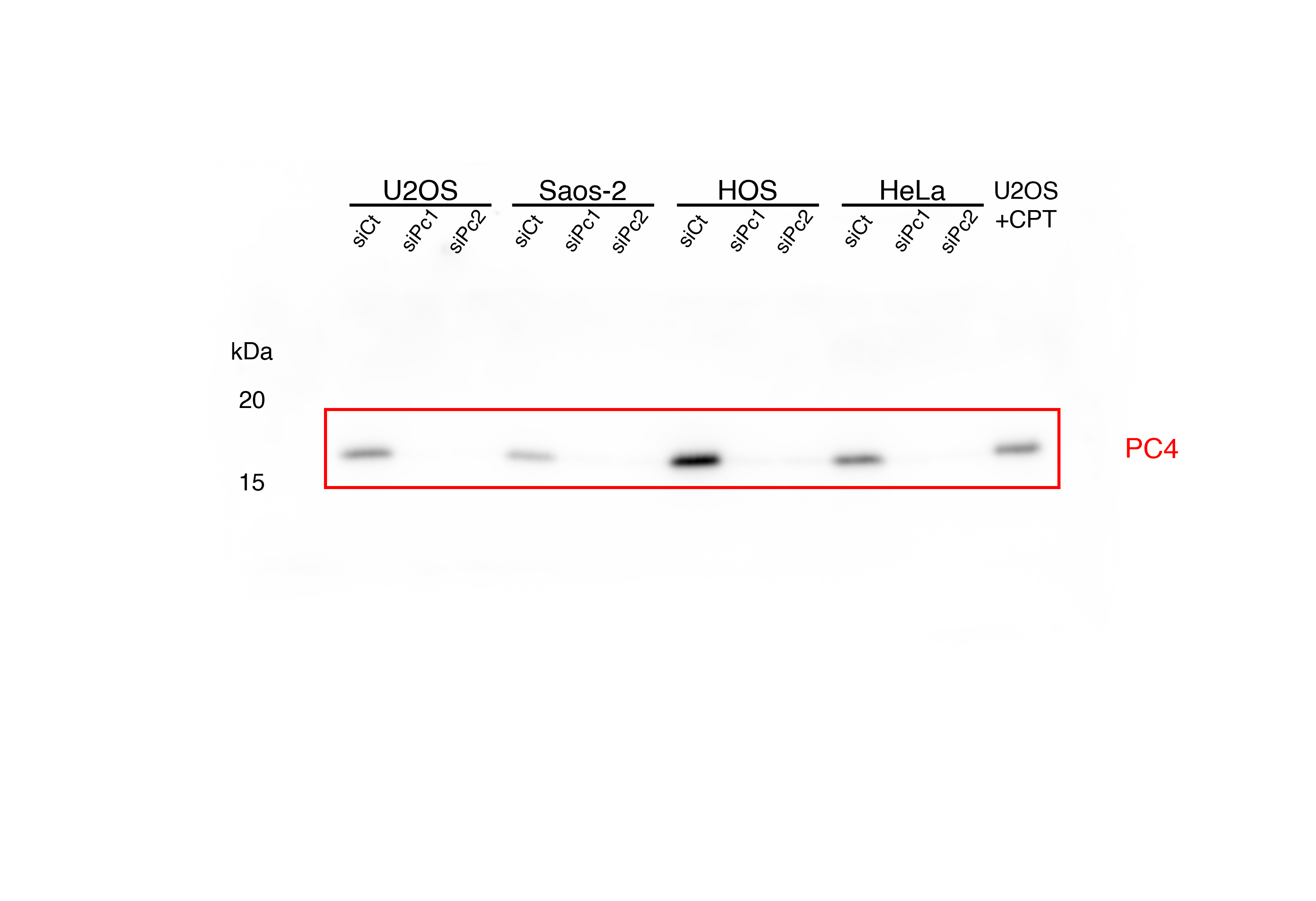

Supplement: Supplementary file 3 — Source data Fig. 2 [file 44319_2024_295_MOESM3_ESM.zip › Figure 2/2E/WesternBlot-PC4.tif]

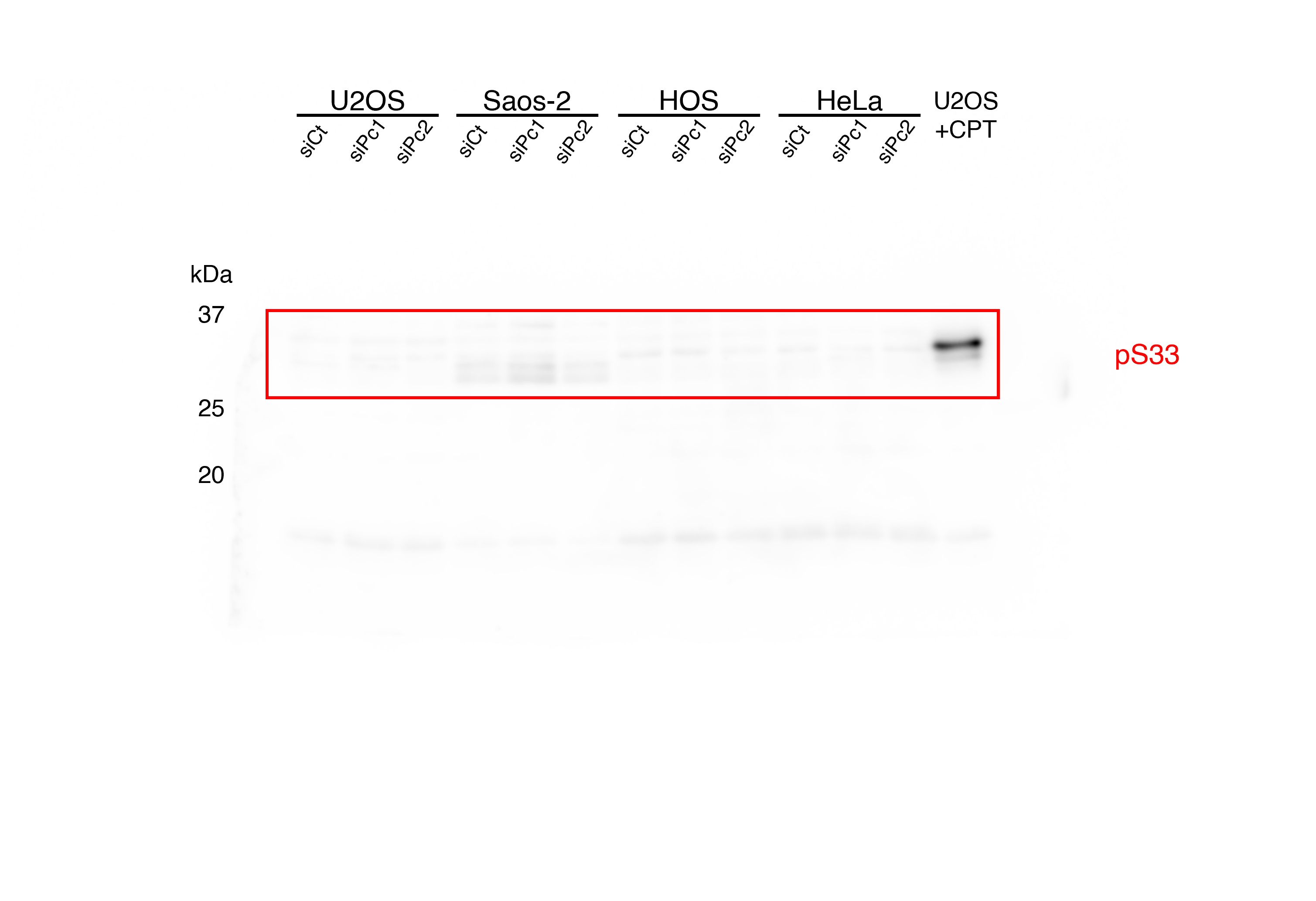

Supplement: Supplementary file 3 — Source data Fig. 2 [file 44319_2024_295_MOESM3_ESM.zip › Figure 2/2E/WesternBlot-pS33.tif]

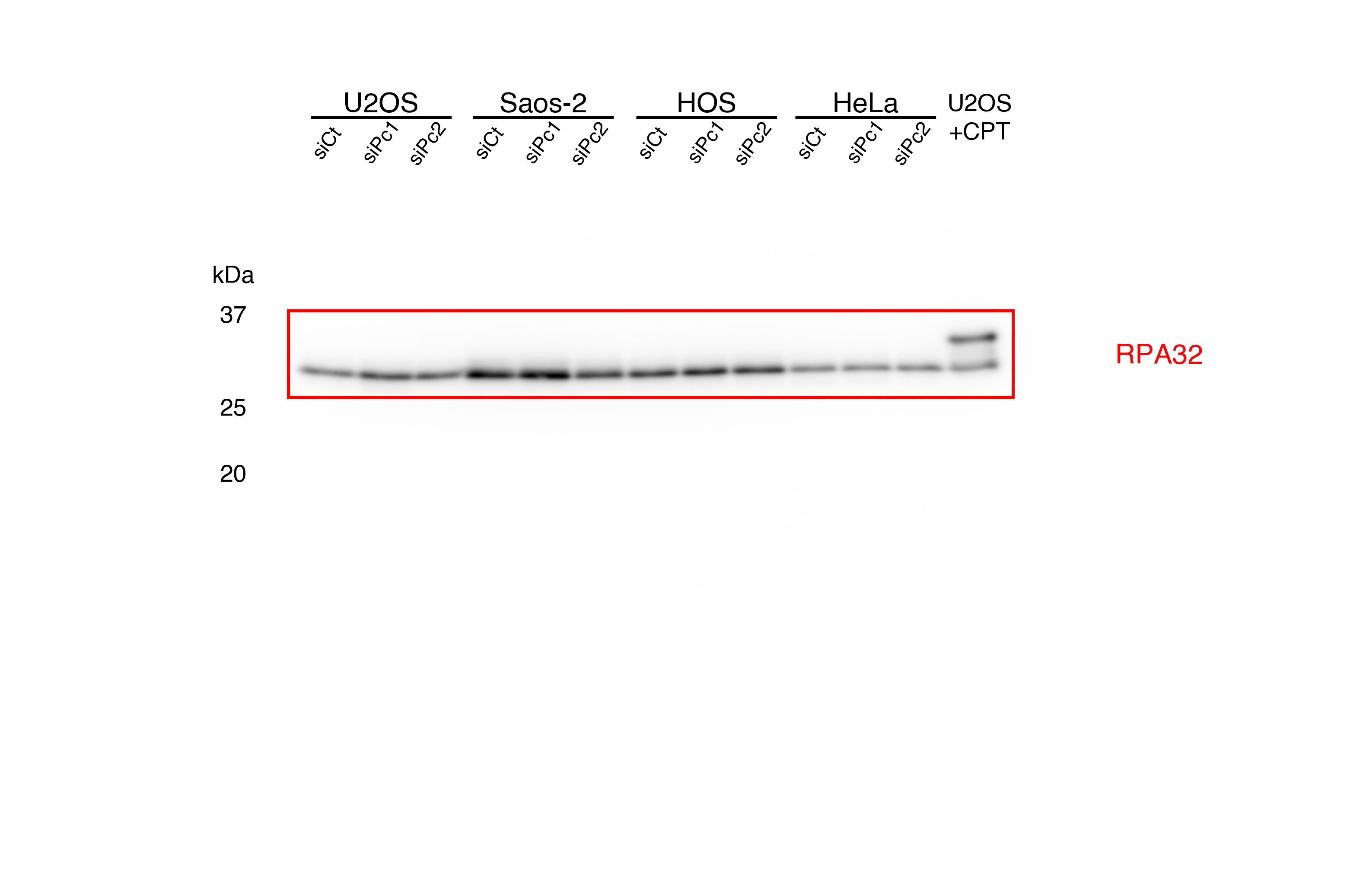

Supplement: Supplementary file 3 — Source data Fig. 2 [file 44319_2024_295_MOESM3_ESM.zip › Figure 2/2E/WesternBlot-RPA32.tif]

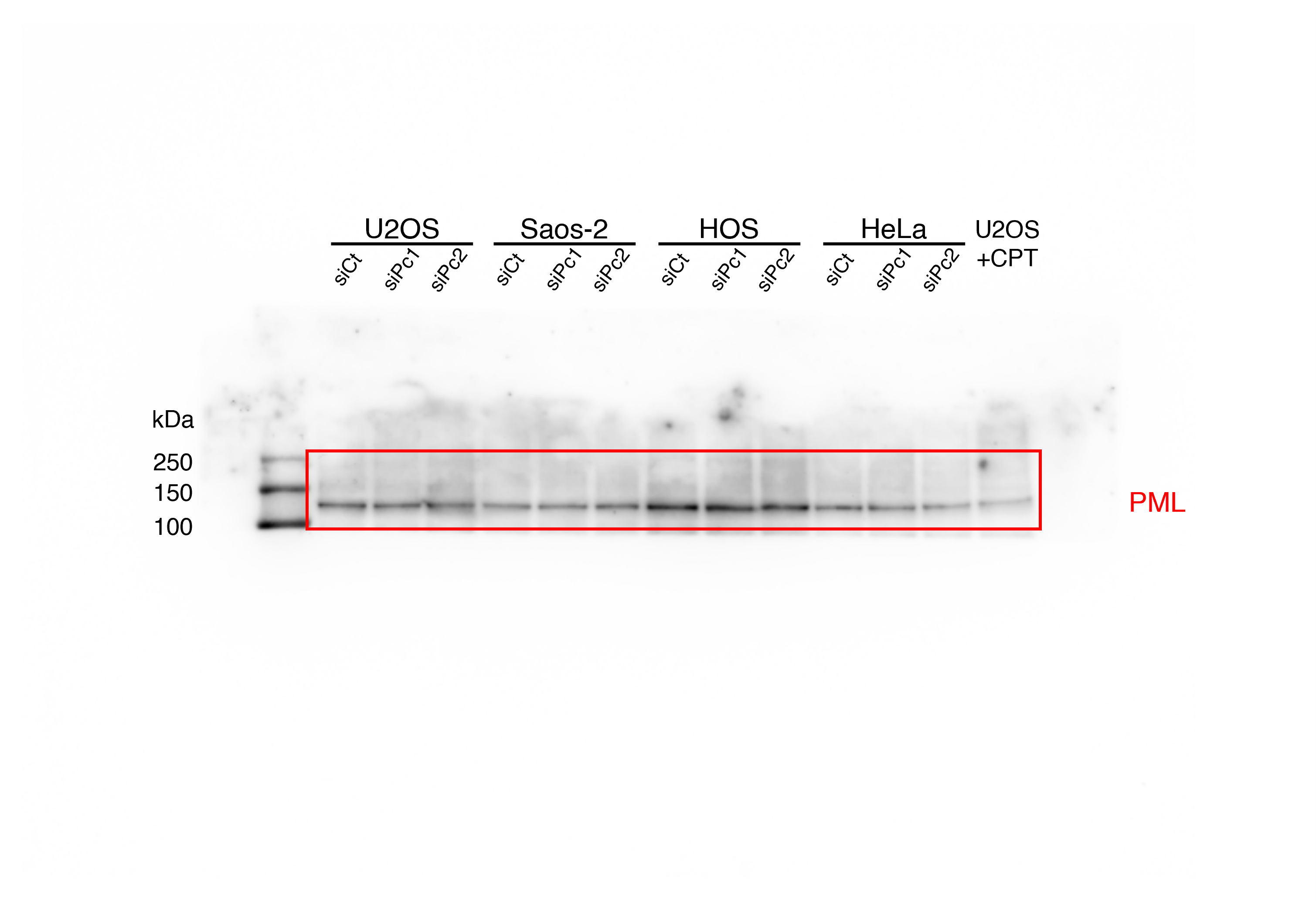

Supplement: Supplementary file 3 — Source data Fig. 2 [file 44319_2024_295_MOESM3_ESM.zip › Figure 2/2E/WesternBlot-PML.tif]

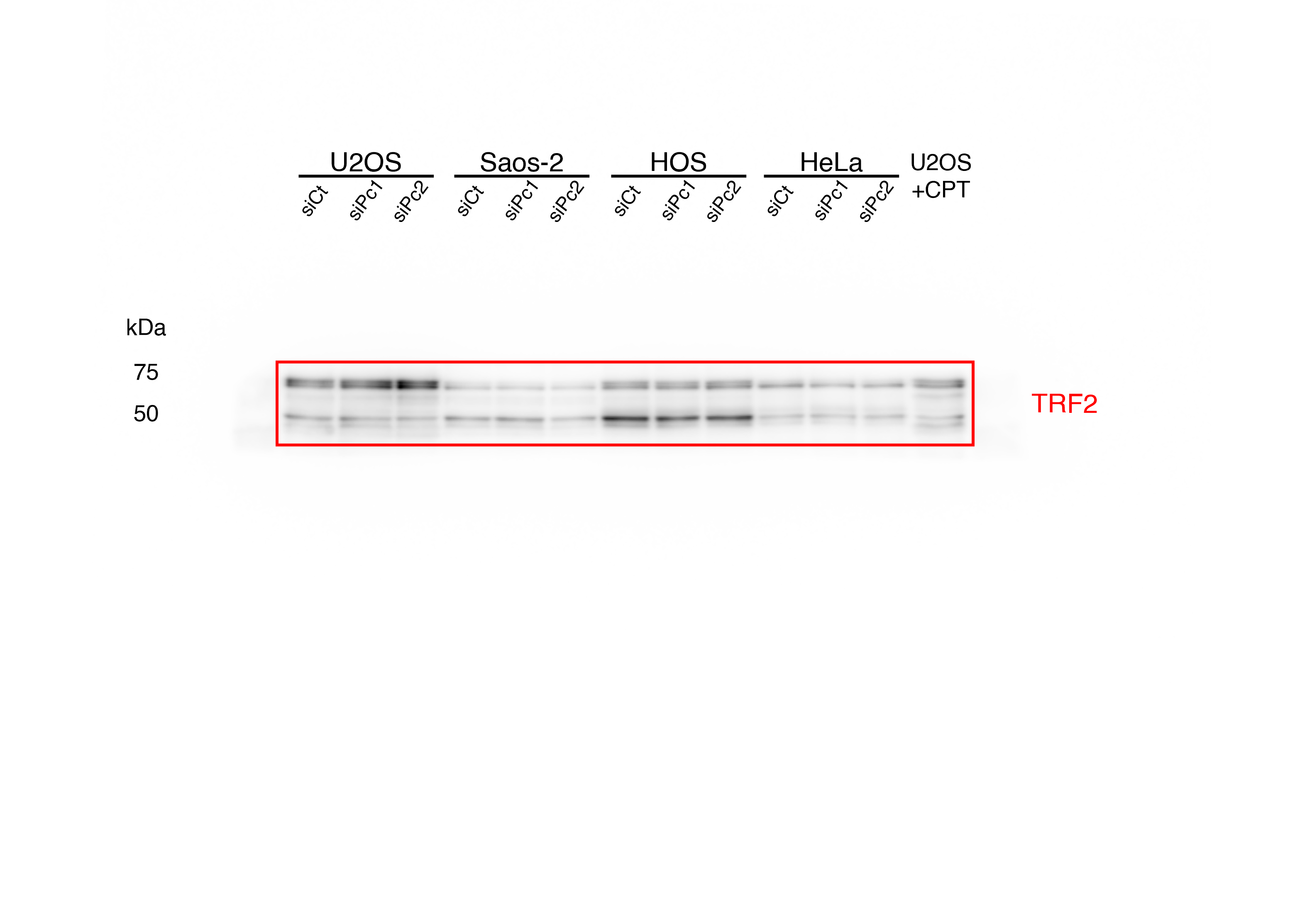

Supplement: Supplementary file 3 — Source data Fig. 2 [file 44319_2024_295_MOESM3_ESM.zip › Figure 2/2E/WesternBlot-TRF2.tif]

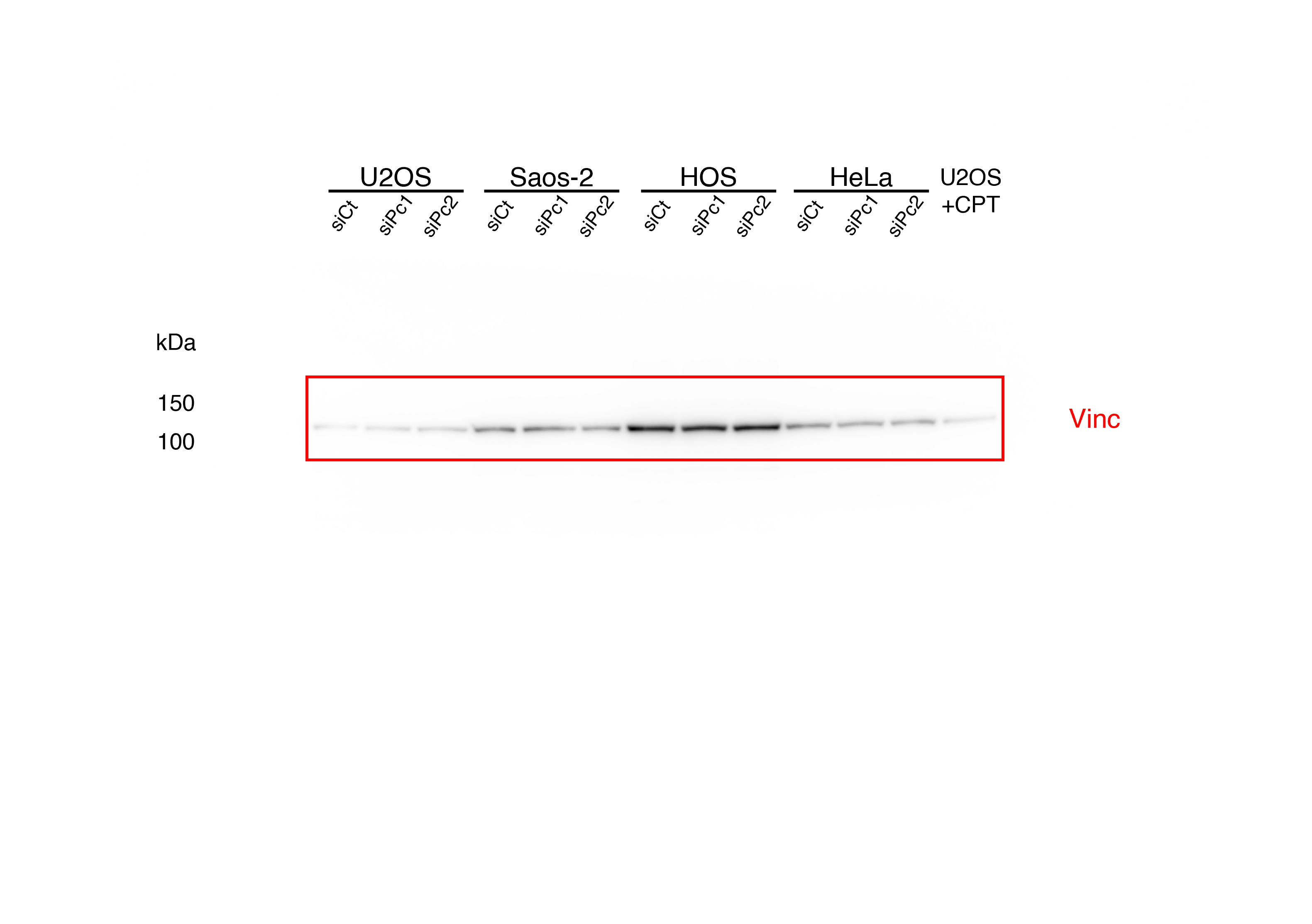

Supplement: Supplementary file 3 — Source data Fig. 2 [file 44319_2024_295_MOESM3_ESM.zip › Figure 2/2E/WesternBlot-Vinculin.tif]

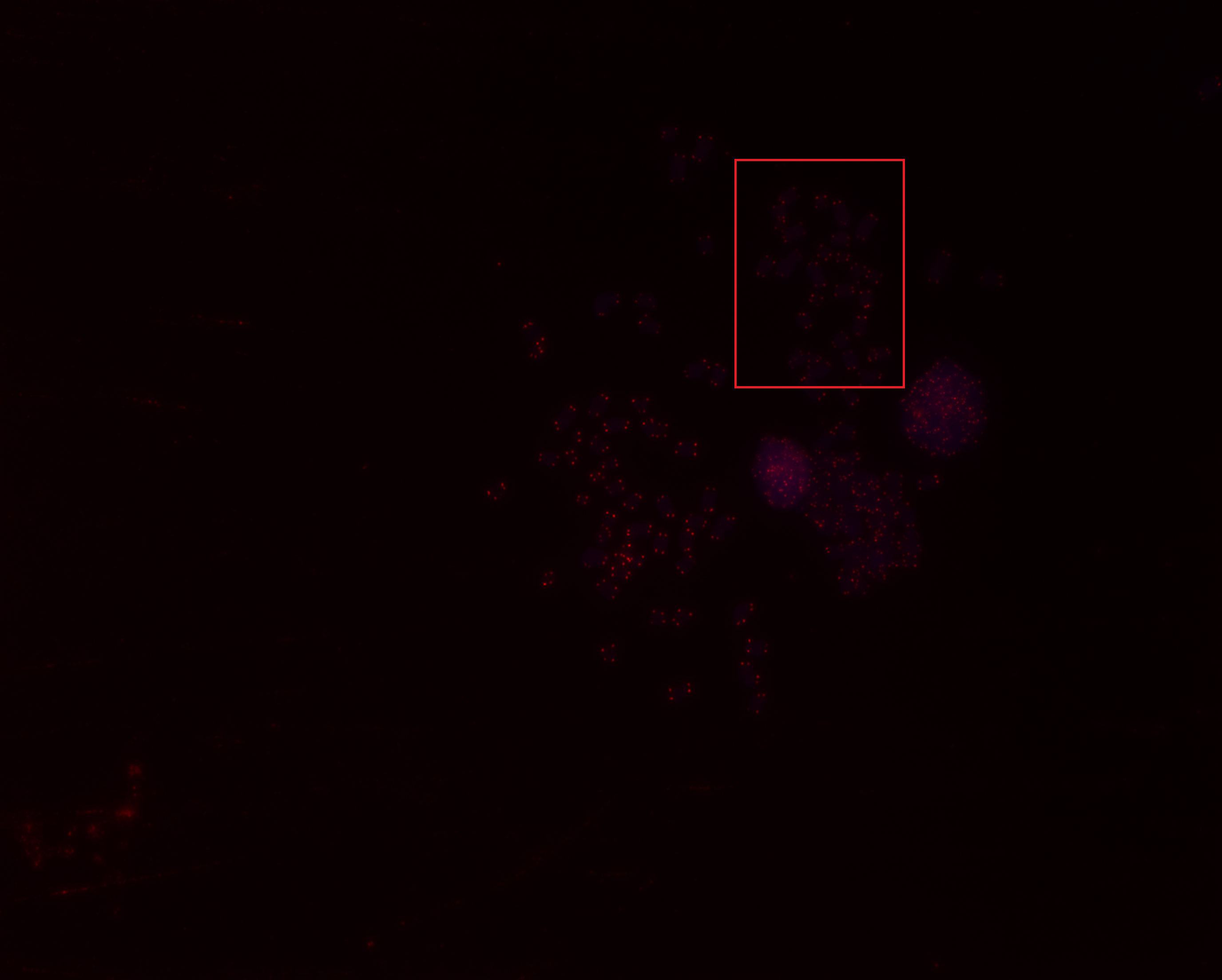

Supplement: Supplementary file 3 — Source data Fig. 2 [file 44319_2024_295_MOESM3_ESM.zip › Figure 2/2B/telomere fragility - representative image-selected area - HOS siPc1.tif]

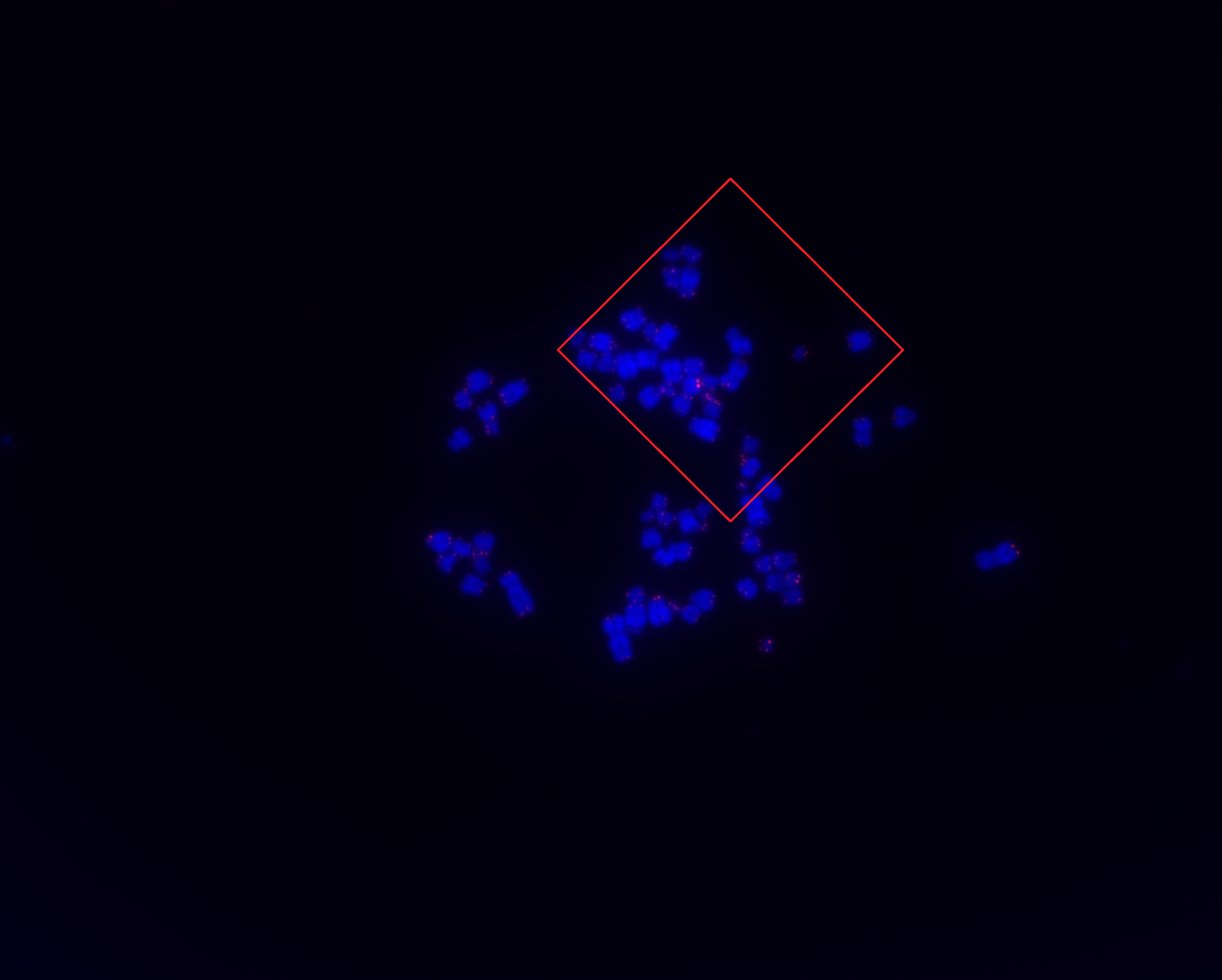

Supplement: Supplementary file 3 — Source data Fig. 2 [file 44319_2024_295_MOESM3_ESM.zip › Figure 2/2B/telomere fragility - representative image-selected area - U2OS siPc1.tif]

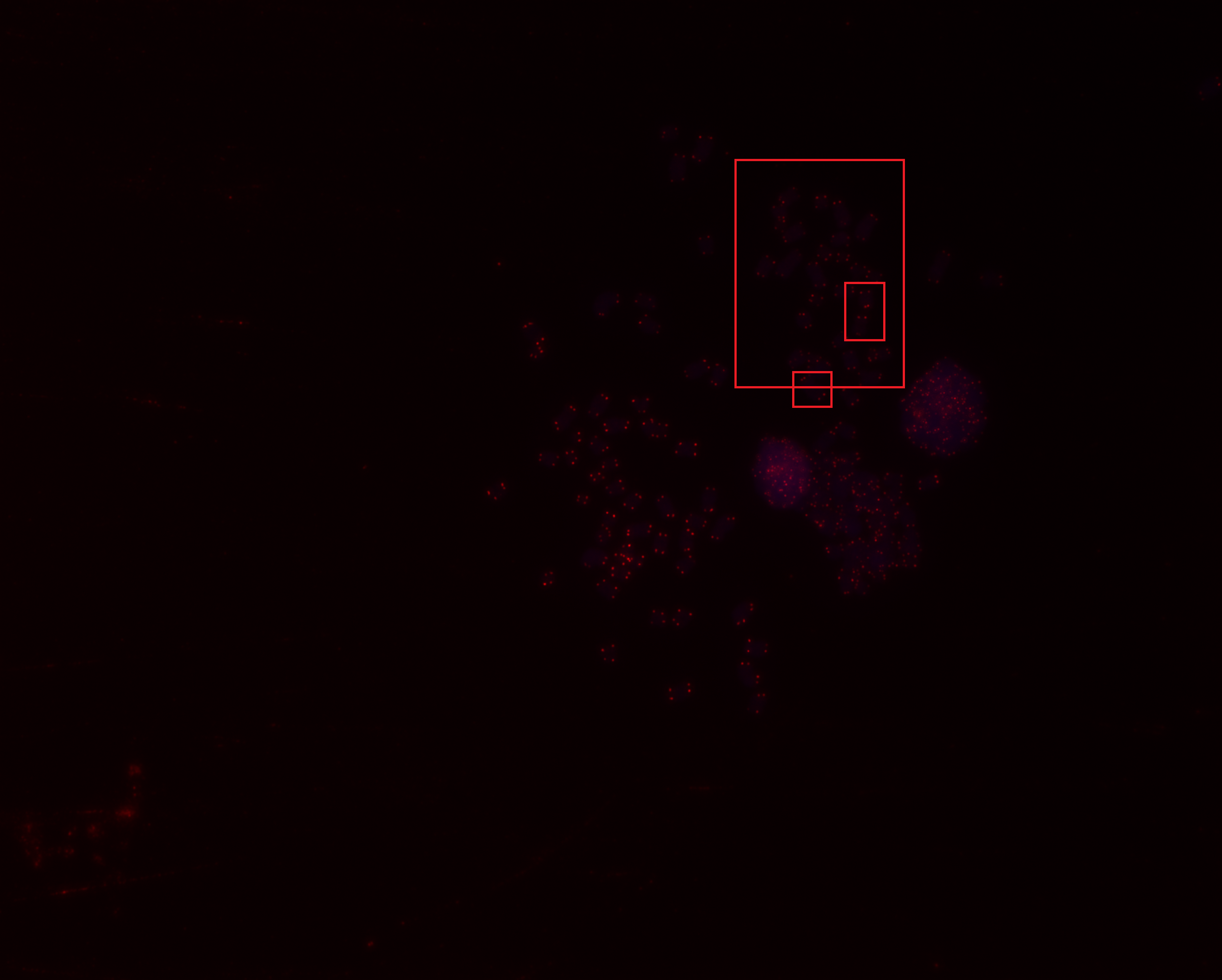

Supplement: Supplementary file 3 — Source data Fig. 2 [file 44319_2024_295_MOESM3_ESM.zip › Figure 2/2B/telomere fragility - representative image-selected areas - HOS siPc1.tif]

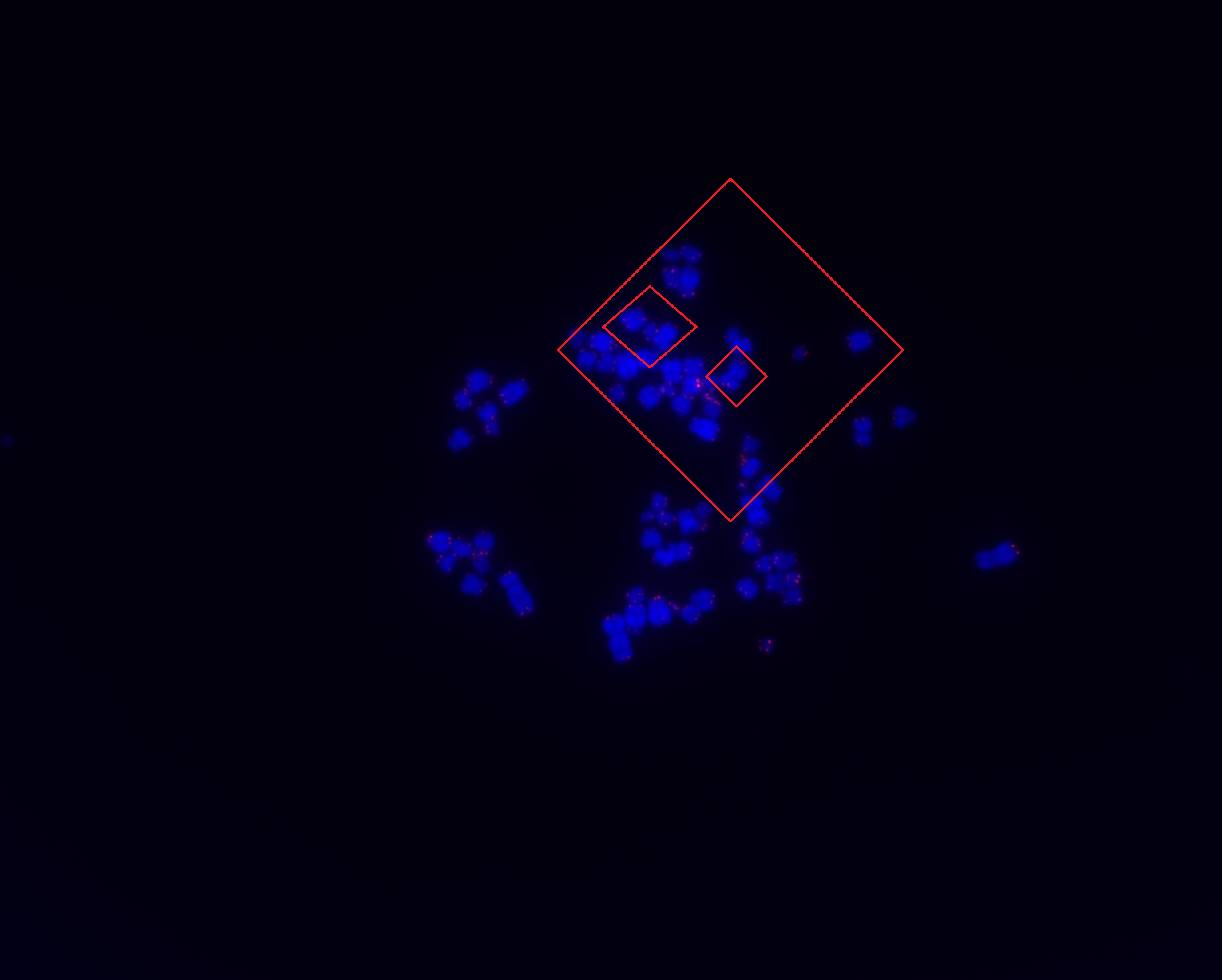

Supplement: Supplementary file 3 — Source data Fig. 2 [file 44319_2024_295_MOESM3_ESM.zip › Figure 2/2B/telomere fragility - representative image-selected areas - U2OS siPc1.tif]

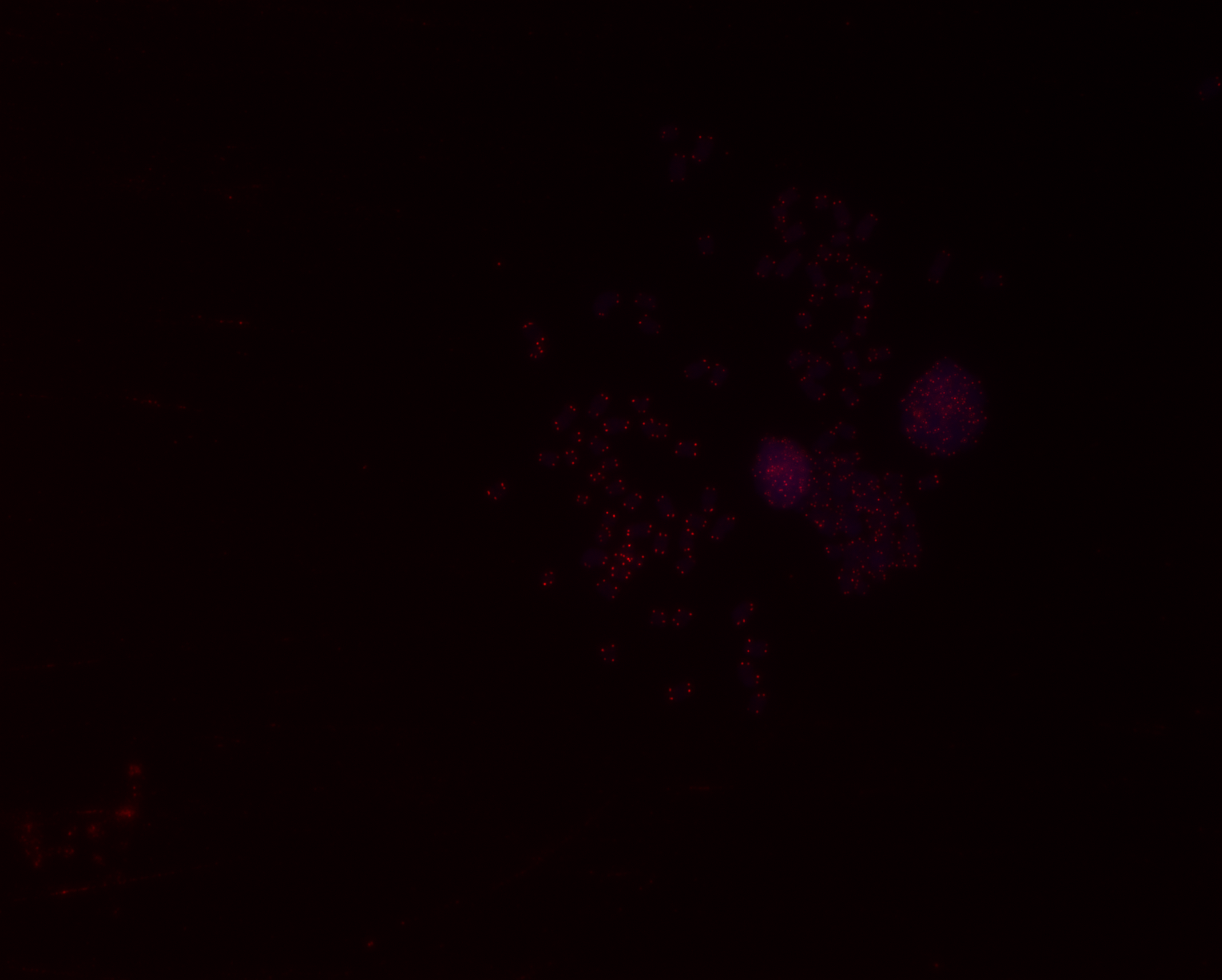

Supplement: Supplementary file 3 — Source data Fig. 2 [file 44319_2024_295_MOESM3_ESM.zip › Figure 2/2B/telomere fragility - representative image- HOS siPc1.tif]

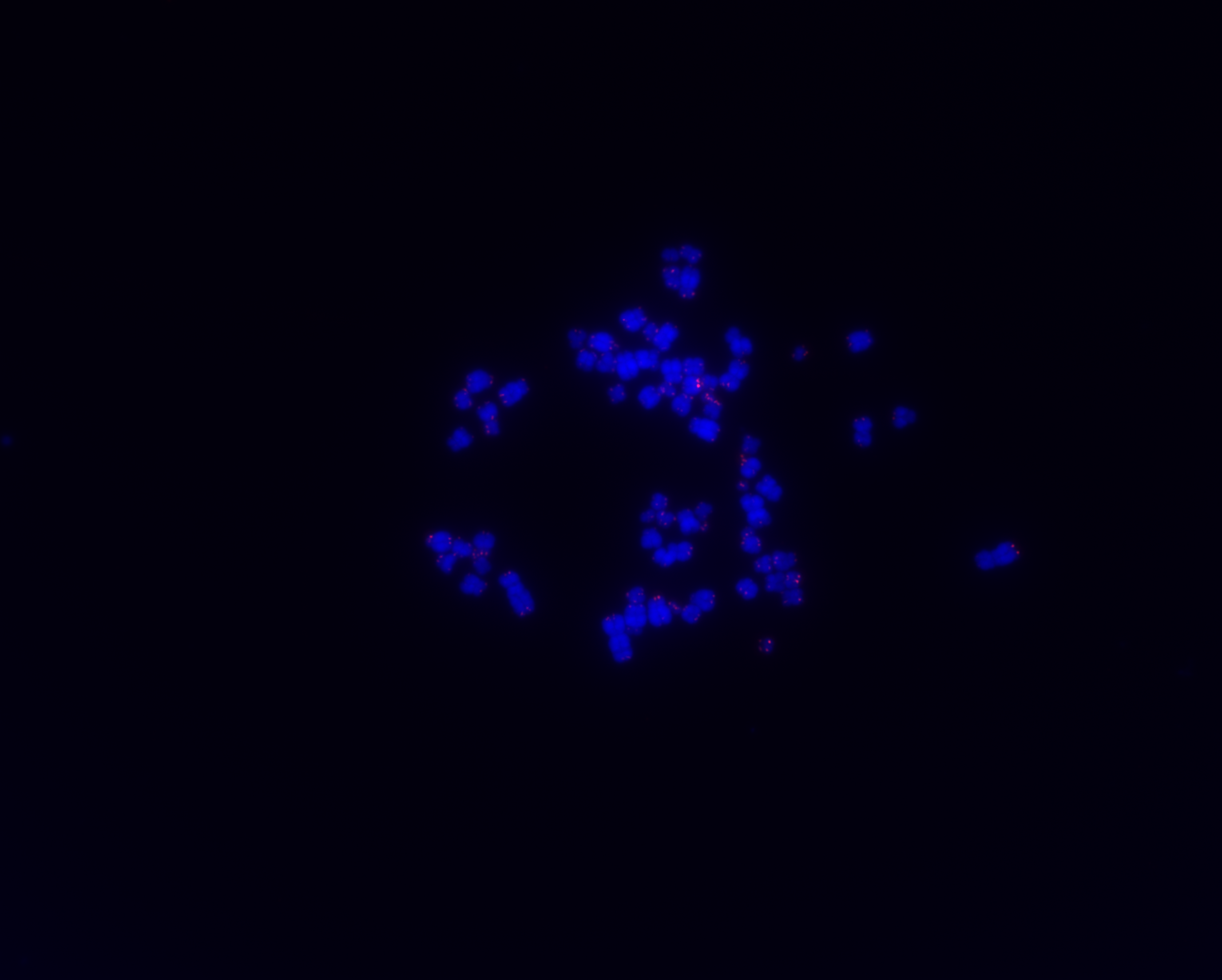

Supplement: Supplementary file 3 — Source data Fig. 2 [file 44319_2024_295_MOESM3_ESM.zip › Figure 2/2B/telomere fragility - representative image- U2OS siPc1.tif]

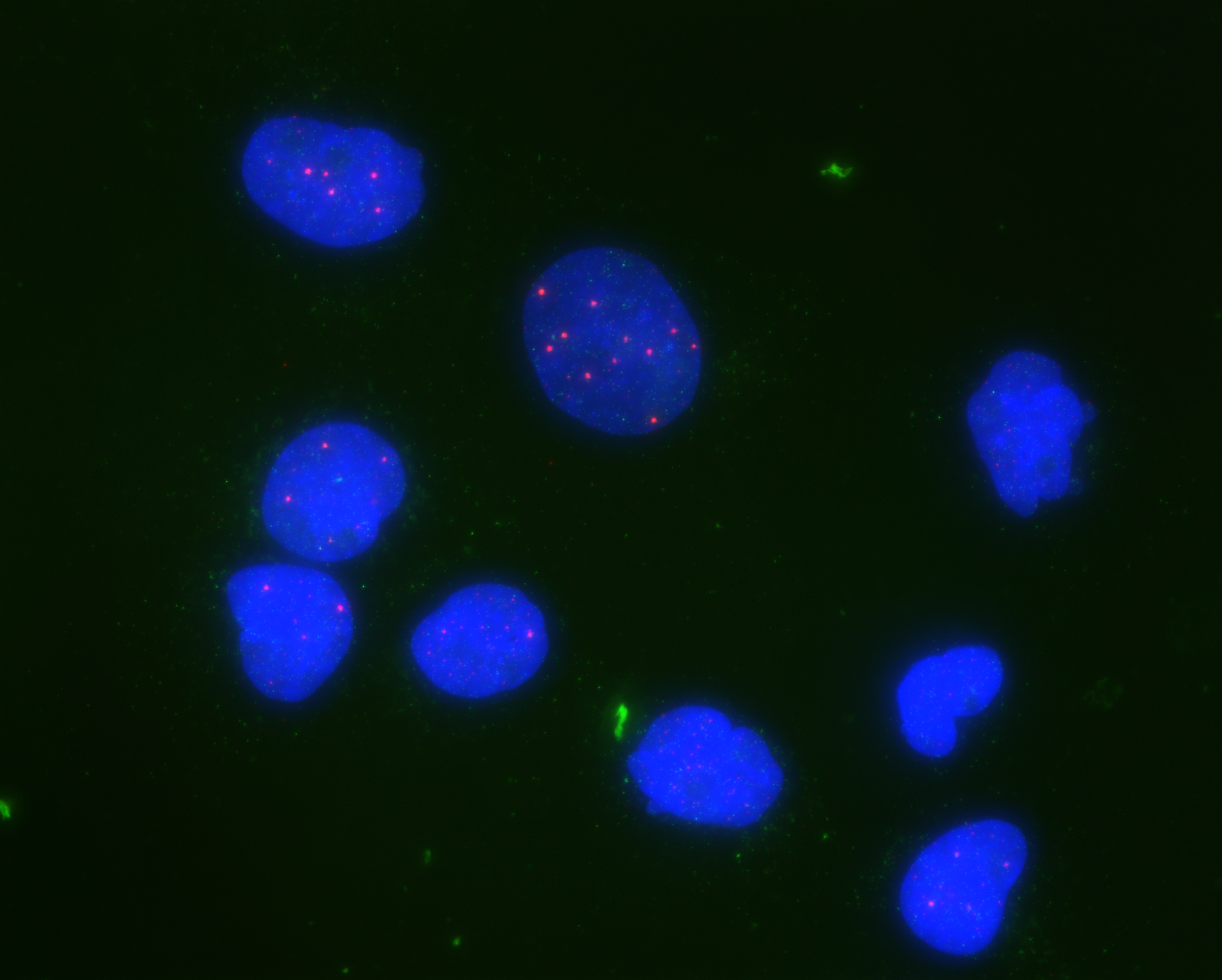

Supplement: Supplementary file 4 — Source data Fig. 3 [file 44319_2024_295_MOESM4_ESM.zip › Figure 3/3B/PC4+TRF2 image - U2OS siFM.tif]

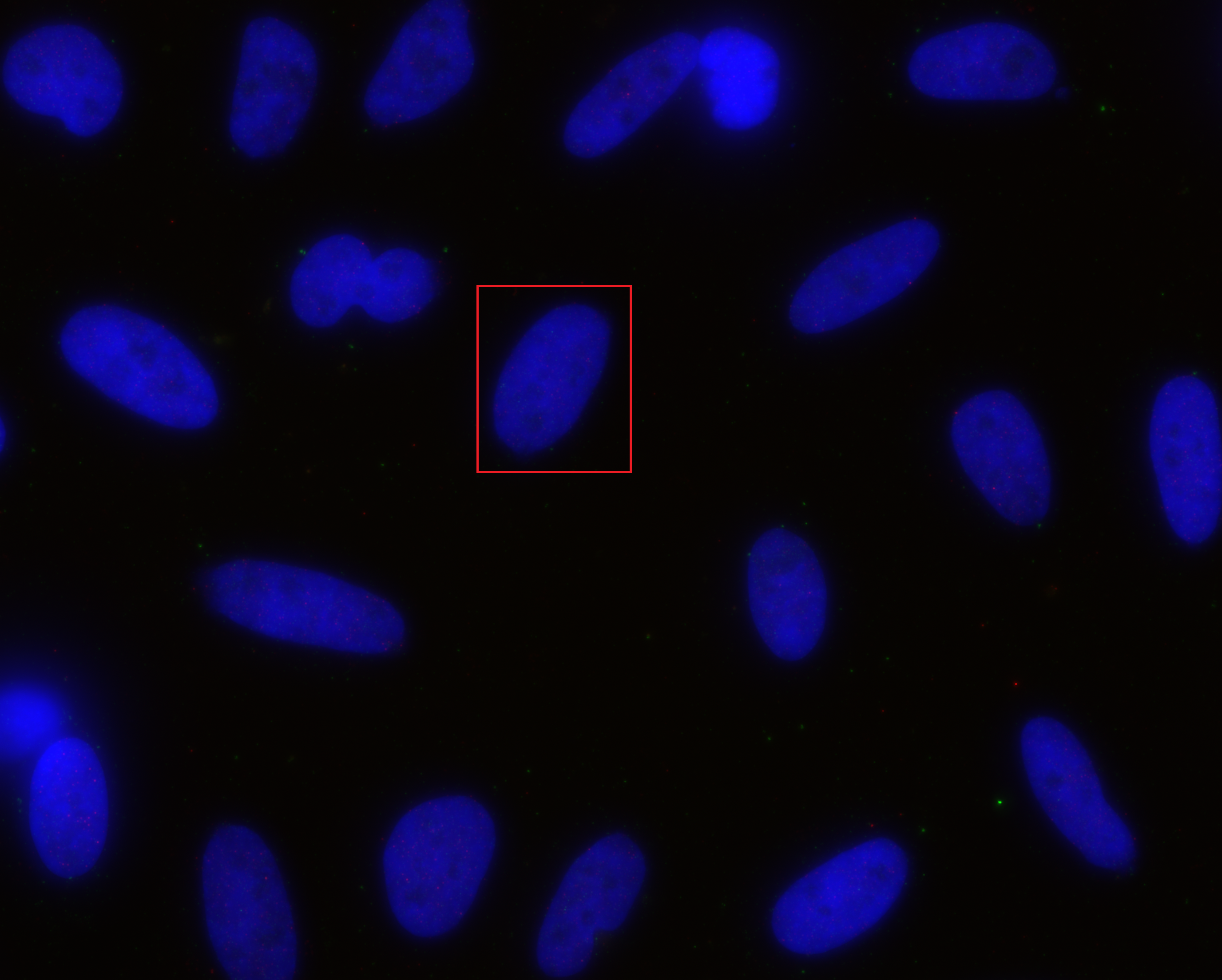

Supplement: Supplementary file 4 — Source data Fig. 3 [file 44319_2024_295_MOESM4_ESM.zip › Figure 3/3B/PC4+TRF2 image - representative nucleus - HOS siFM.tif]

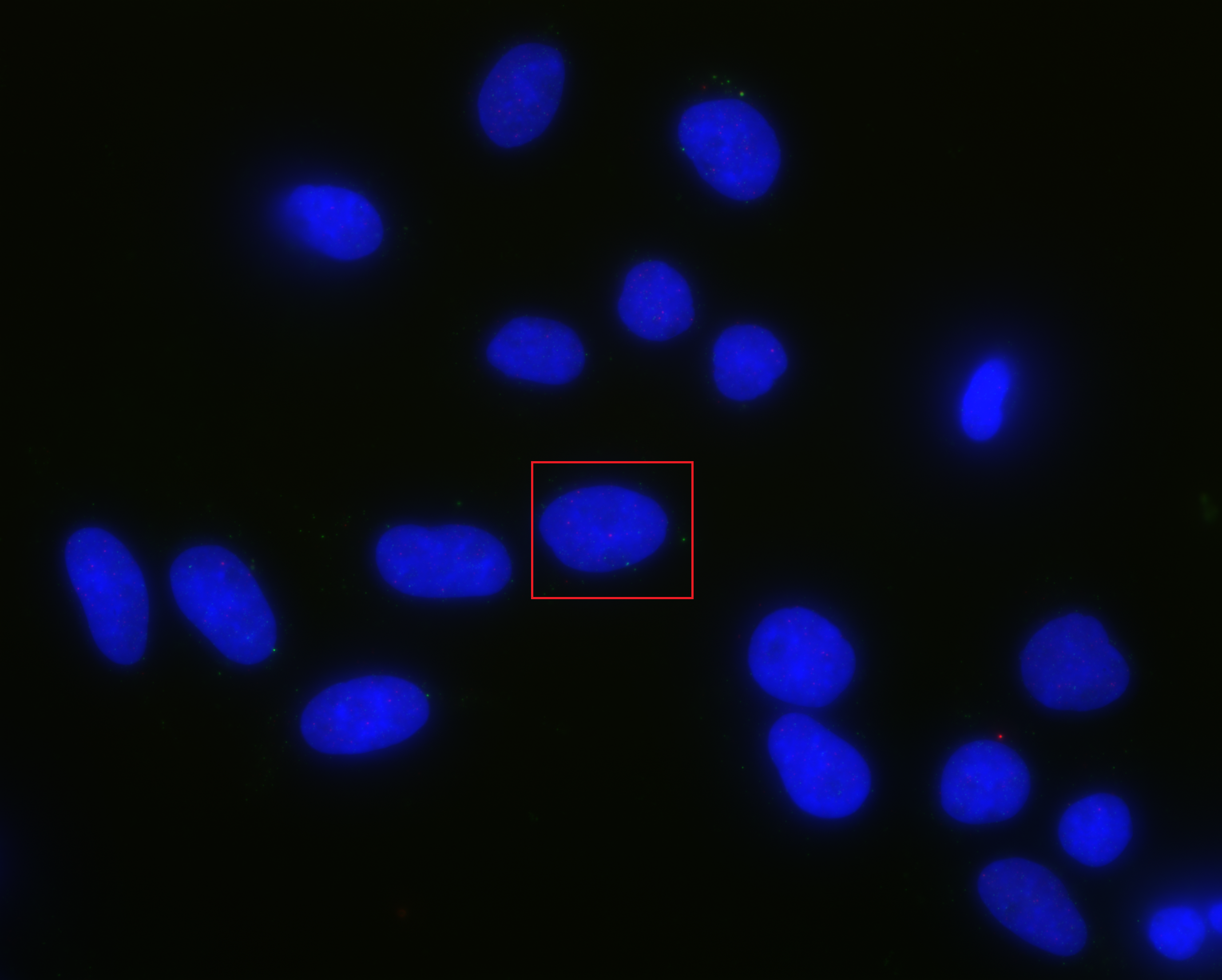

Supplement: Supplementary file 4 — Source data Fig. 3 [file 44319_2024_295_MOESM4_ESM.zip › Figure 3/3B/PC4+TRF2 image - representative nucleus - Saos2 siCt.tif]

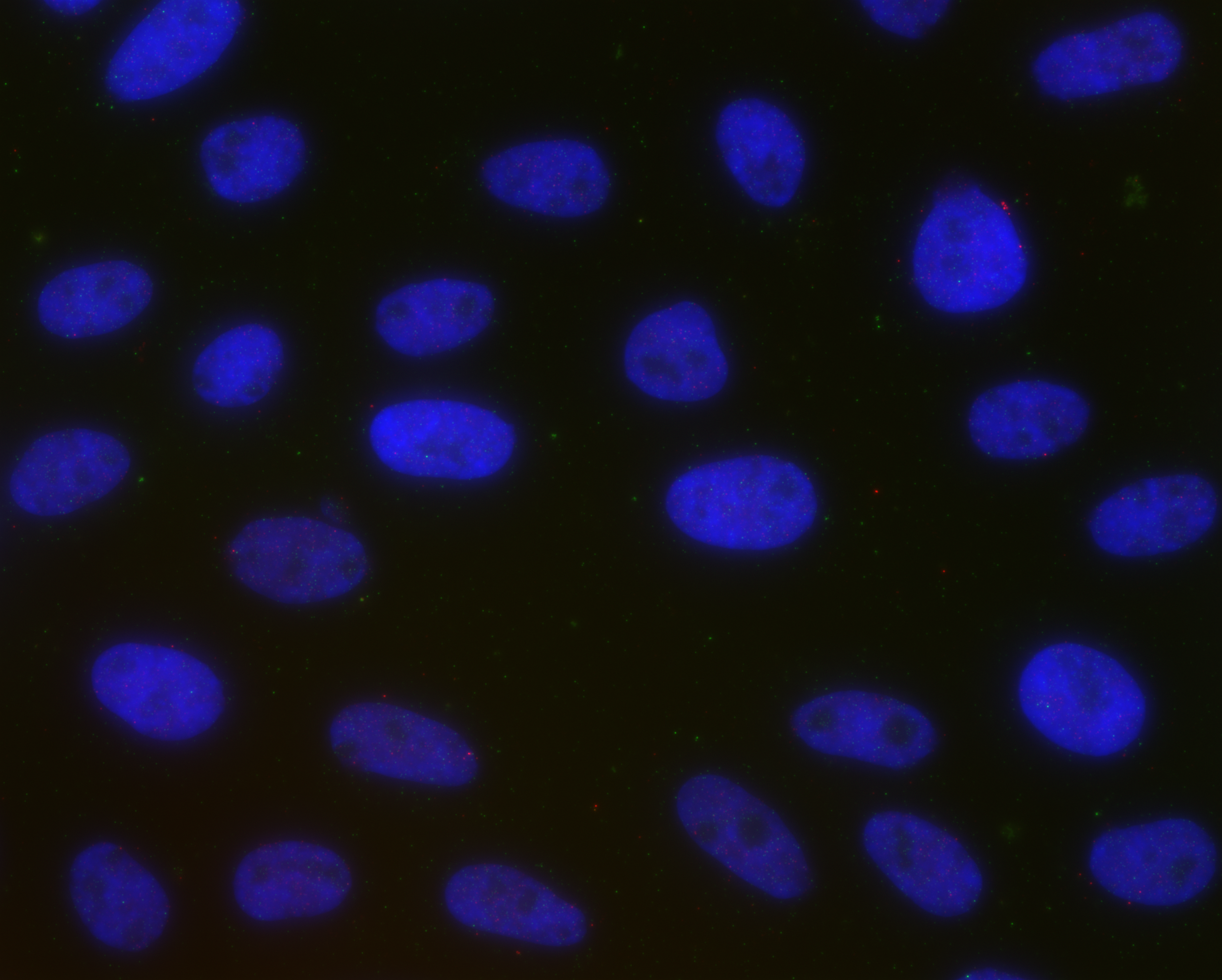

Supplement: Supplementary file 4 — Source data Fig. 3 [file 44319_2024_295_MOESM4_ESM.zip › Figure 3/3B/PC4+TRF2 image - HOS siCt.tif]

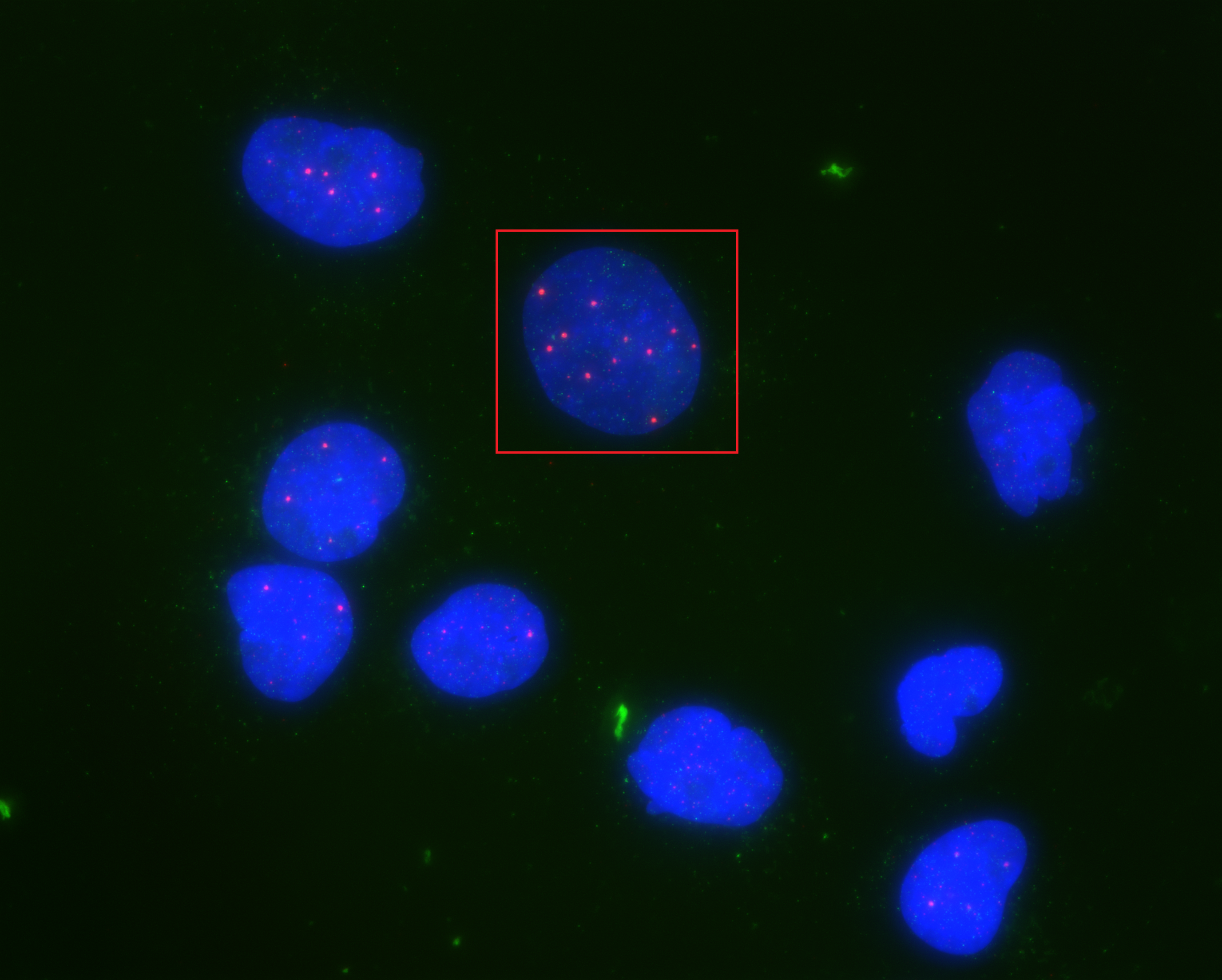

Supplement: Supplementary file 4 — Source data Fig. 3 [file 44319_2024_295_MOESM4_ESM.zip › Figure 3/3B/PC4+TRF2 image - representative nucleus - U2OS siFM.tif]

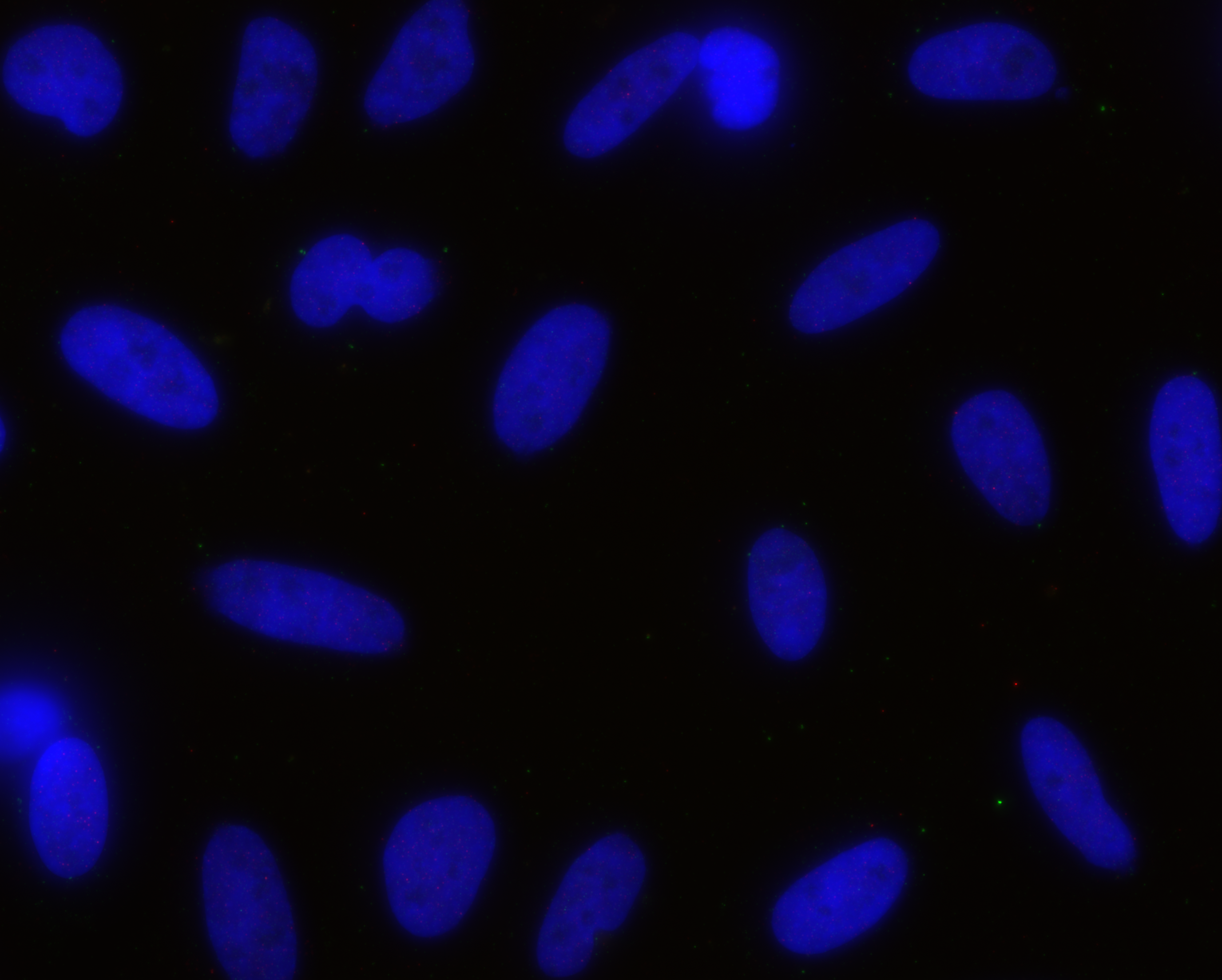

Supplement: Supplementary file 4 — Source data Fig. 3 [file 44319_2024_295_MOESM4_ESM.zip › Figure 3/3B/PC4+TRF2 image - HOS siFM.tif]

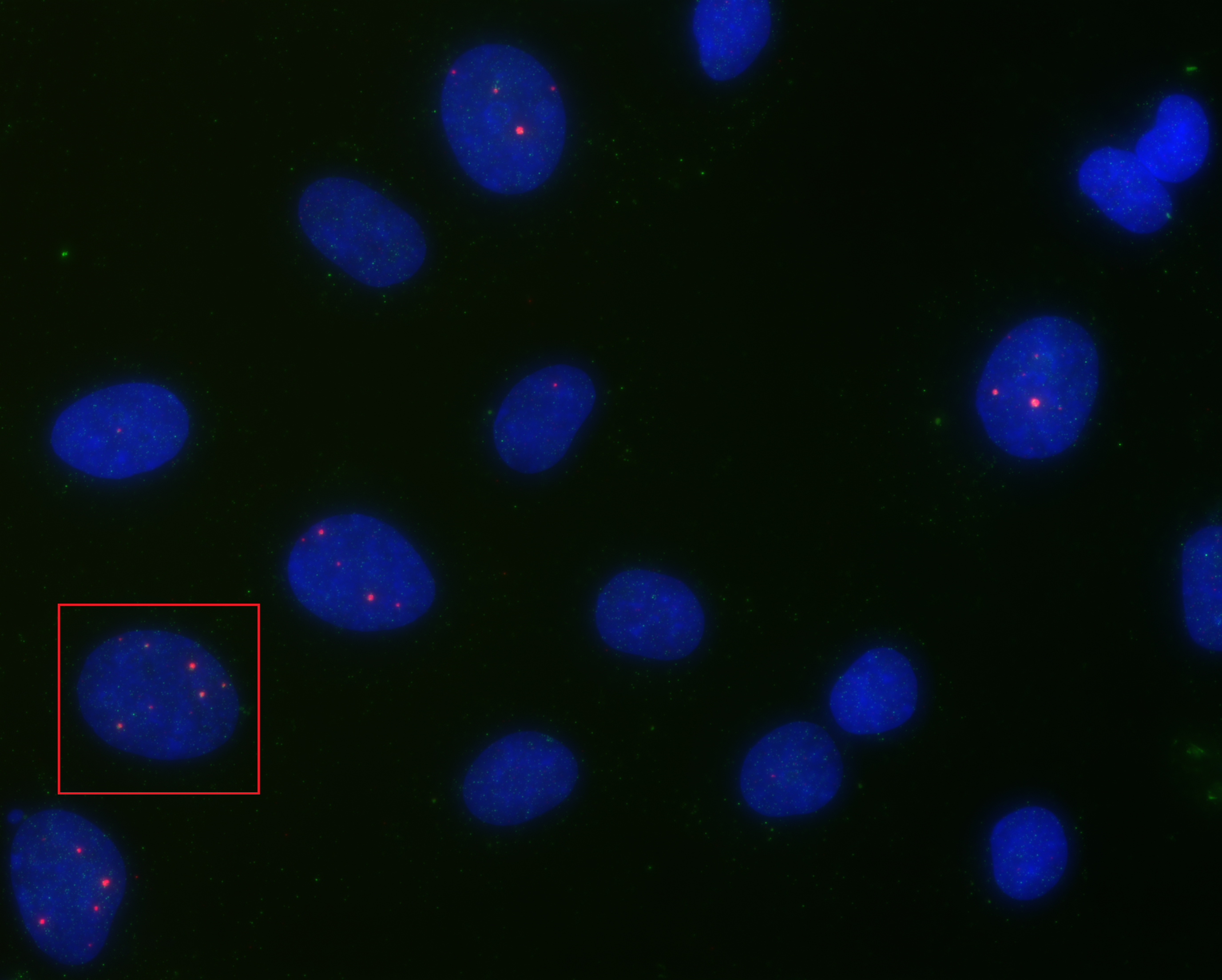

Supplement: Supplementary file 4 — Source data Fig. 3 [file 44319_2024_295_MOESM4_ESM.zip › Figure 3/3B/PC4+TRF2 image - representative nucleus - Saos2 siFM.tif]

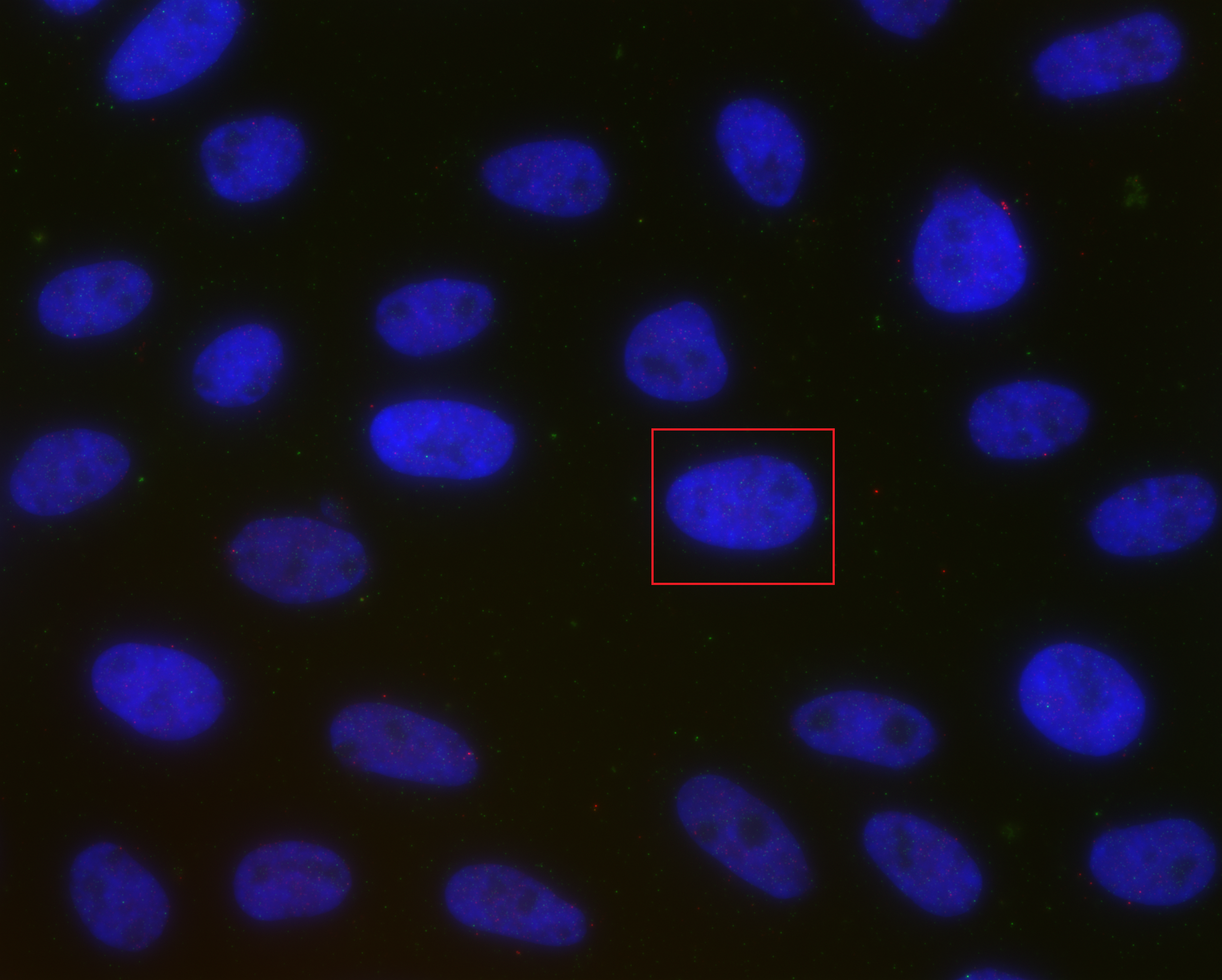

Supplement: Supplementary file 4 — Source data Fig. 3 [file 44319_2024_295_MOESM4_ESM.zip › Figure 3/3B/PC4+TRF2 image - representative nucleus - HOS siCt.tif]

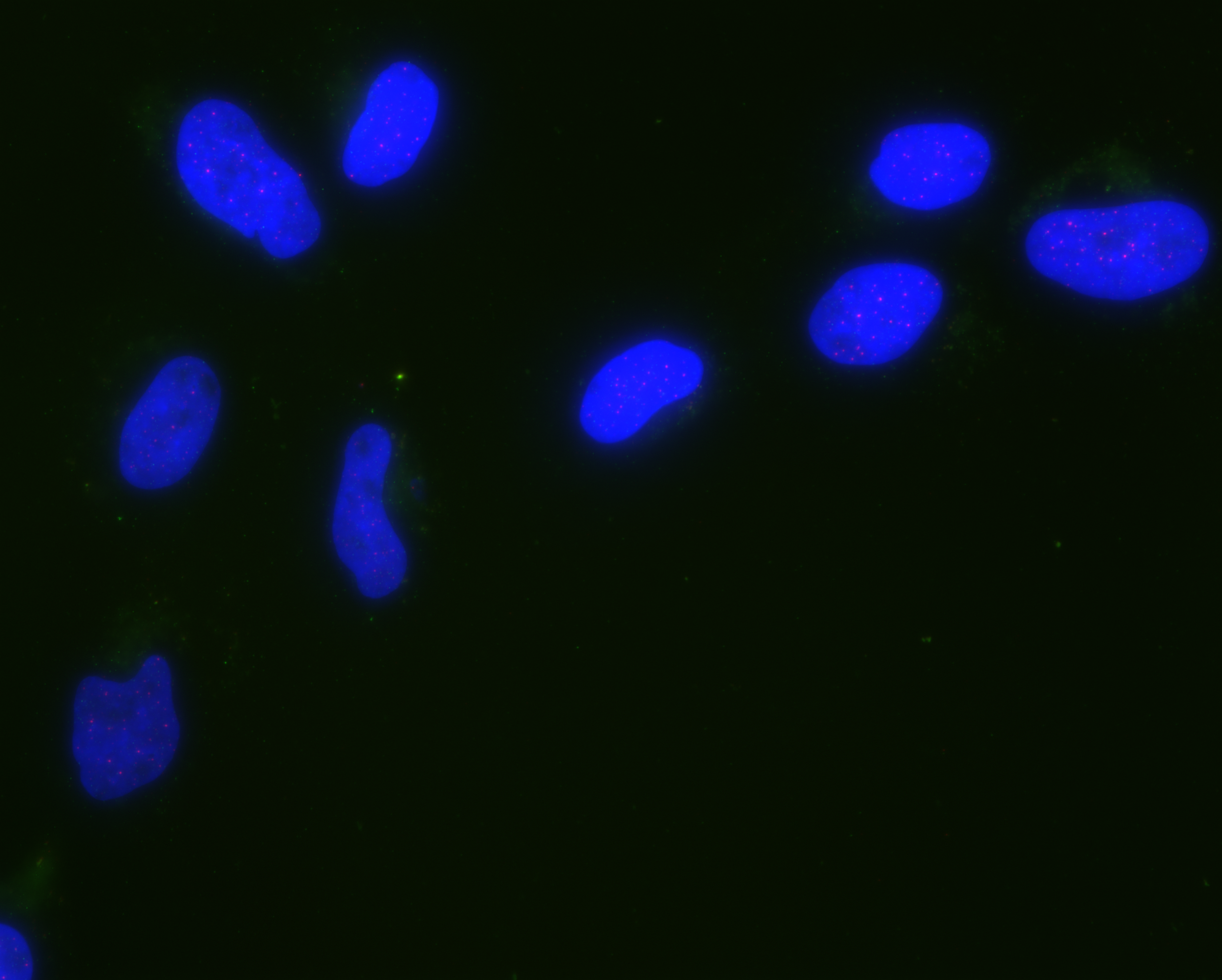

Supplement: Supplementary file 4 — Source data Fig. 3 [file 44319_2024_295_MOESM4_ESM.zip › Figure 3/3B/PC4+TRF2 image - U2OS siCt.tif]

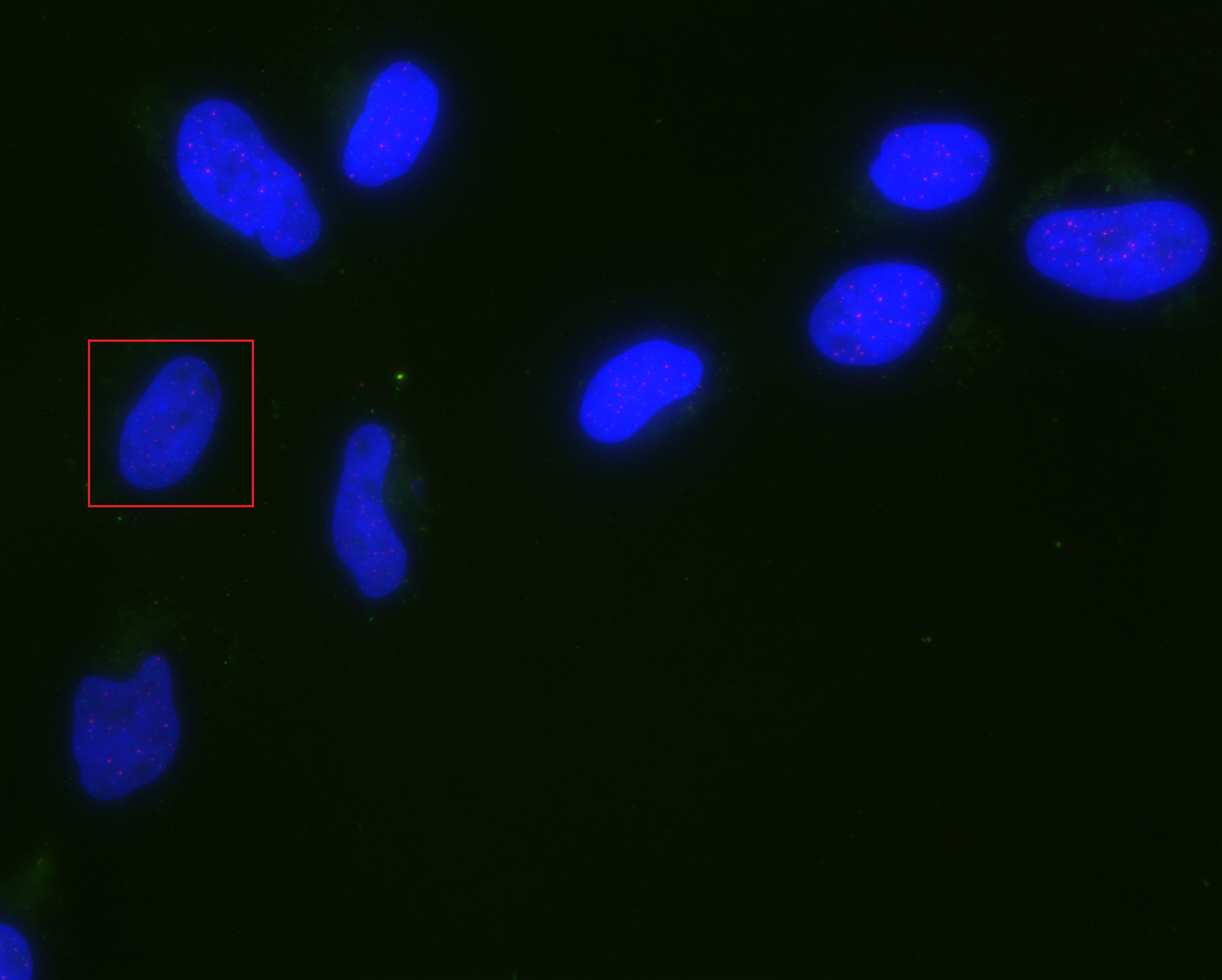

Supplement: Supplementary file 4 — Source data Fig. 3 [file 44319_2024_295_MOESM4_ESM.zip › Figure 3/3B/PC4+TRF2 image - representative nucleus - U2OS siCt.tif]

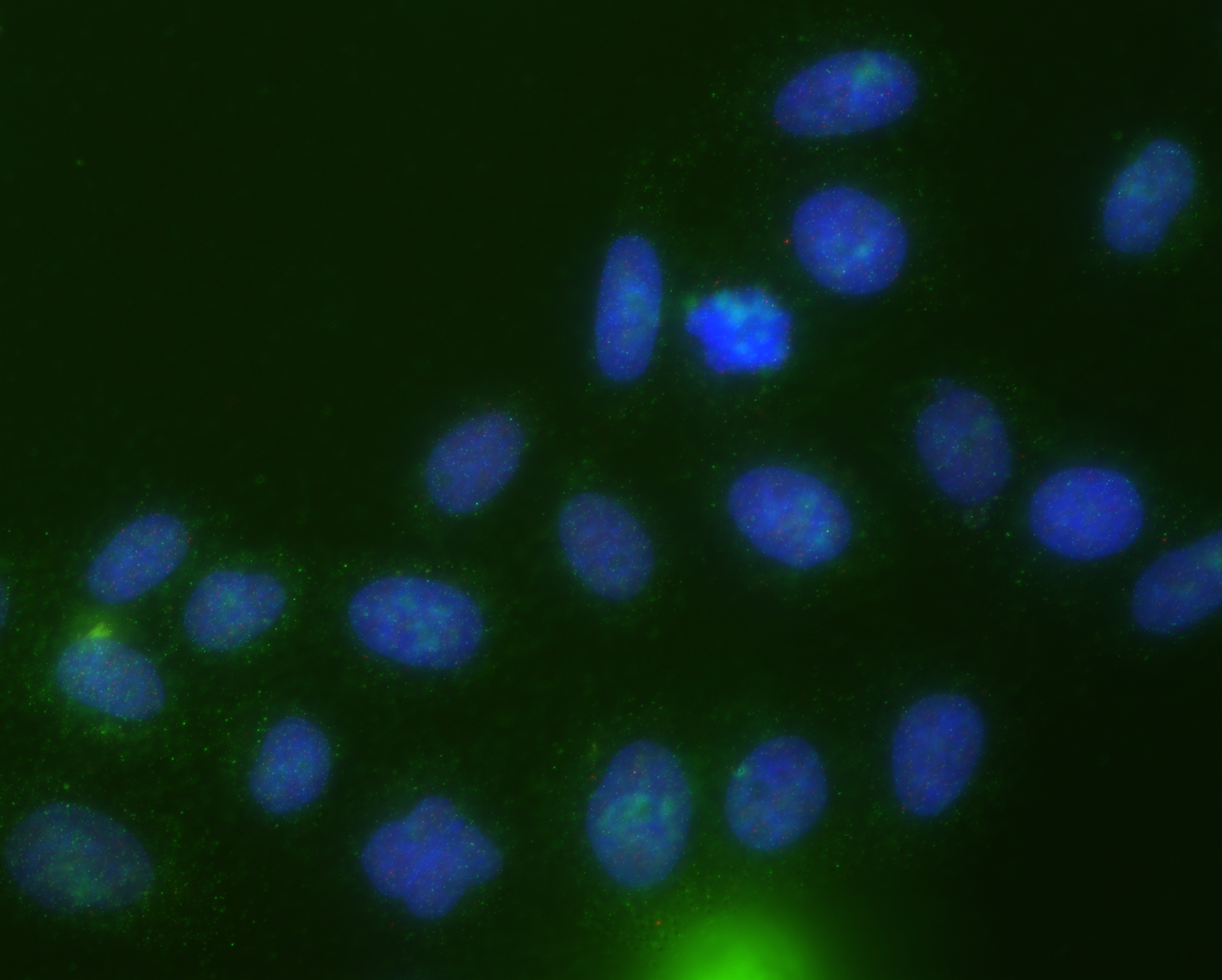

Supplement: Supplementary file 4 — Source data Fig. 3 [file 44319_2024_295_MOESM4_ESM.zip › Figure 3/3B/PC4+TRF2 image - HeLa siCt.tif]

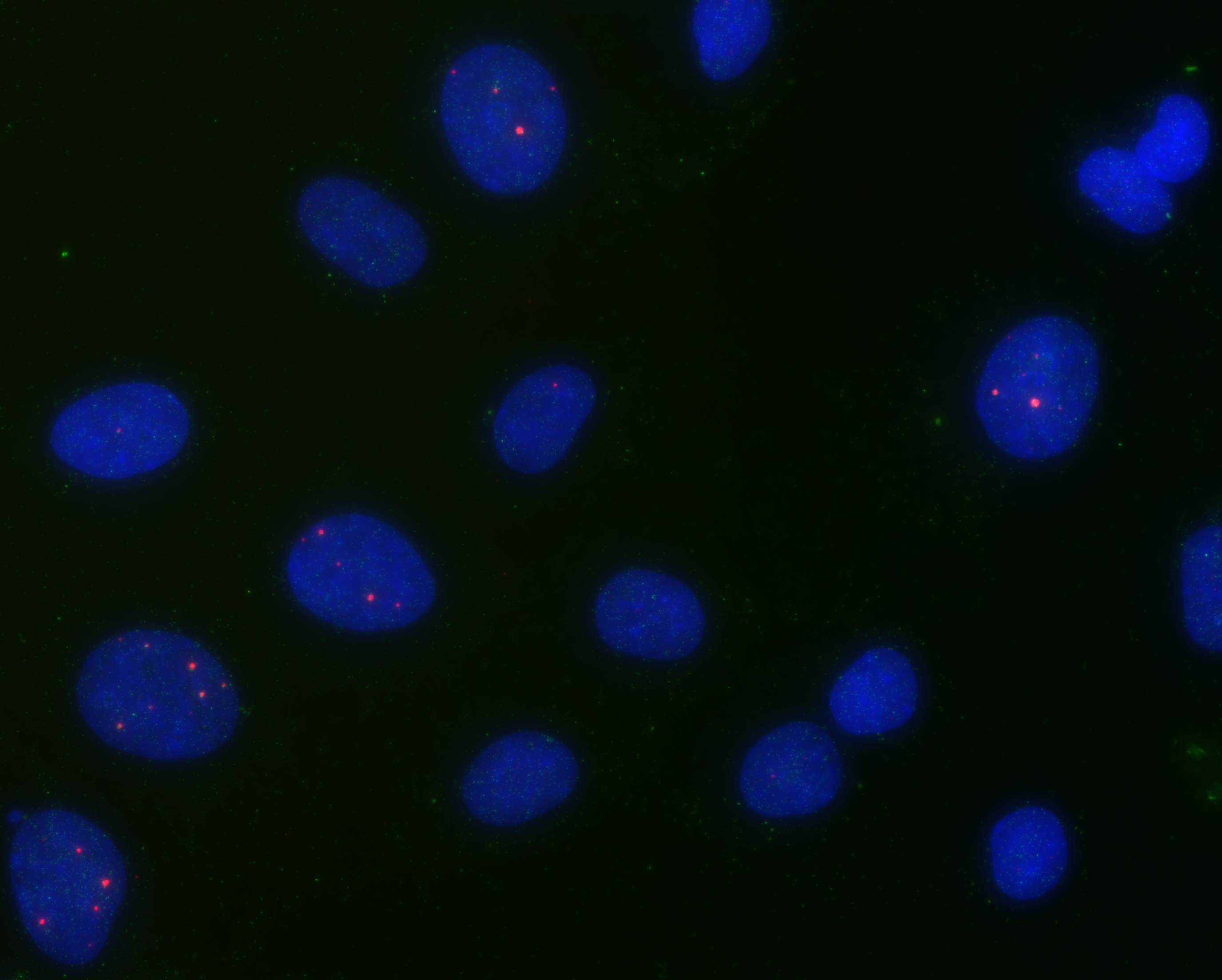

Supplement: Supplementary file 4 — Source data Fig. 3 [file 44319_2024_295_MOESM4_ESM.zip › Figure 3/3B/PC4+TRF2 image - Saos2 siFM.tif]

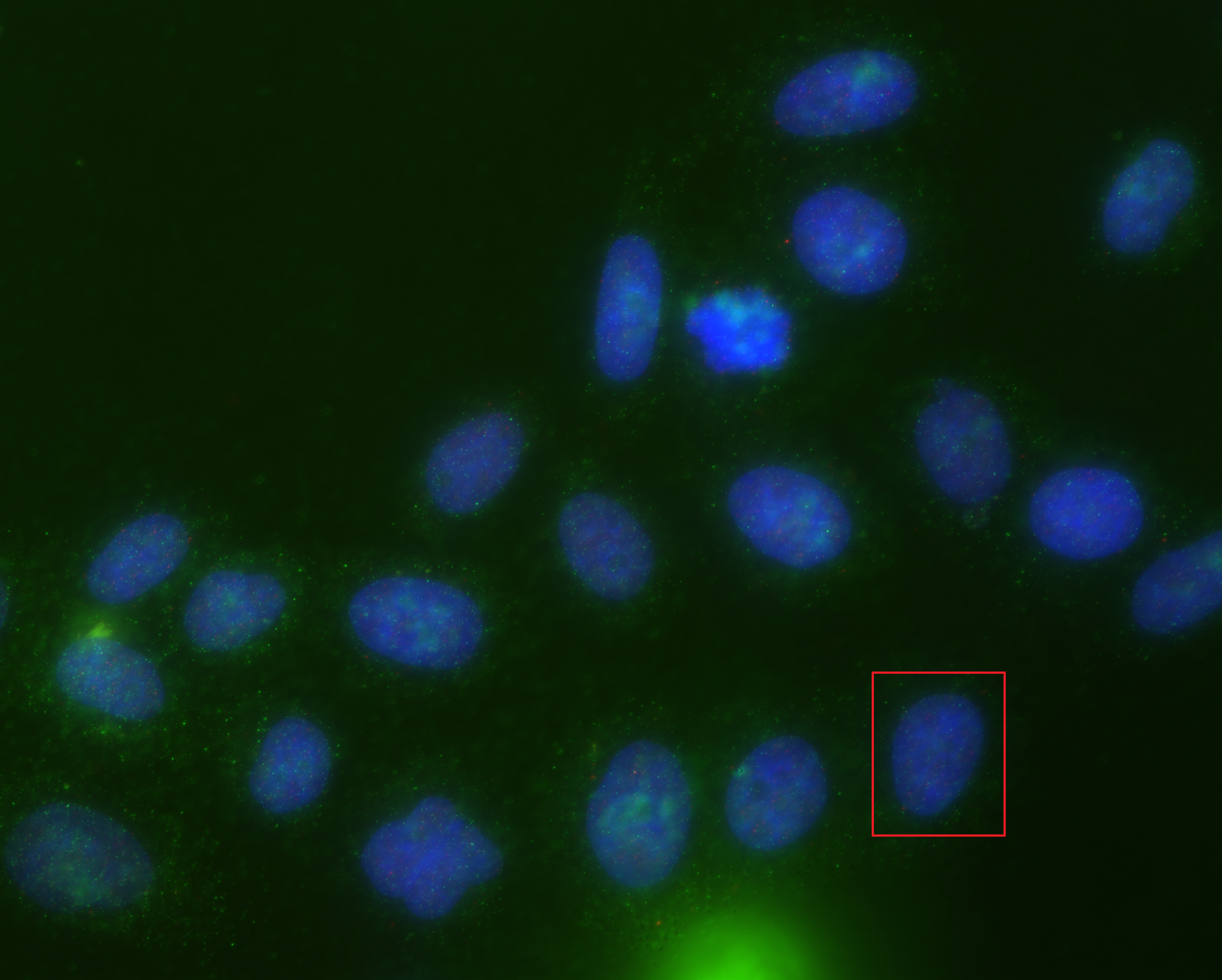

Supplement: Supplementary file 4 — Source data Fig. 3 [file 44319_2024_295_MOESM4_ESM.zip › Figure 3/3B/PC4+TRF2 image - representative nucleus - HeLa siCt.tif]

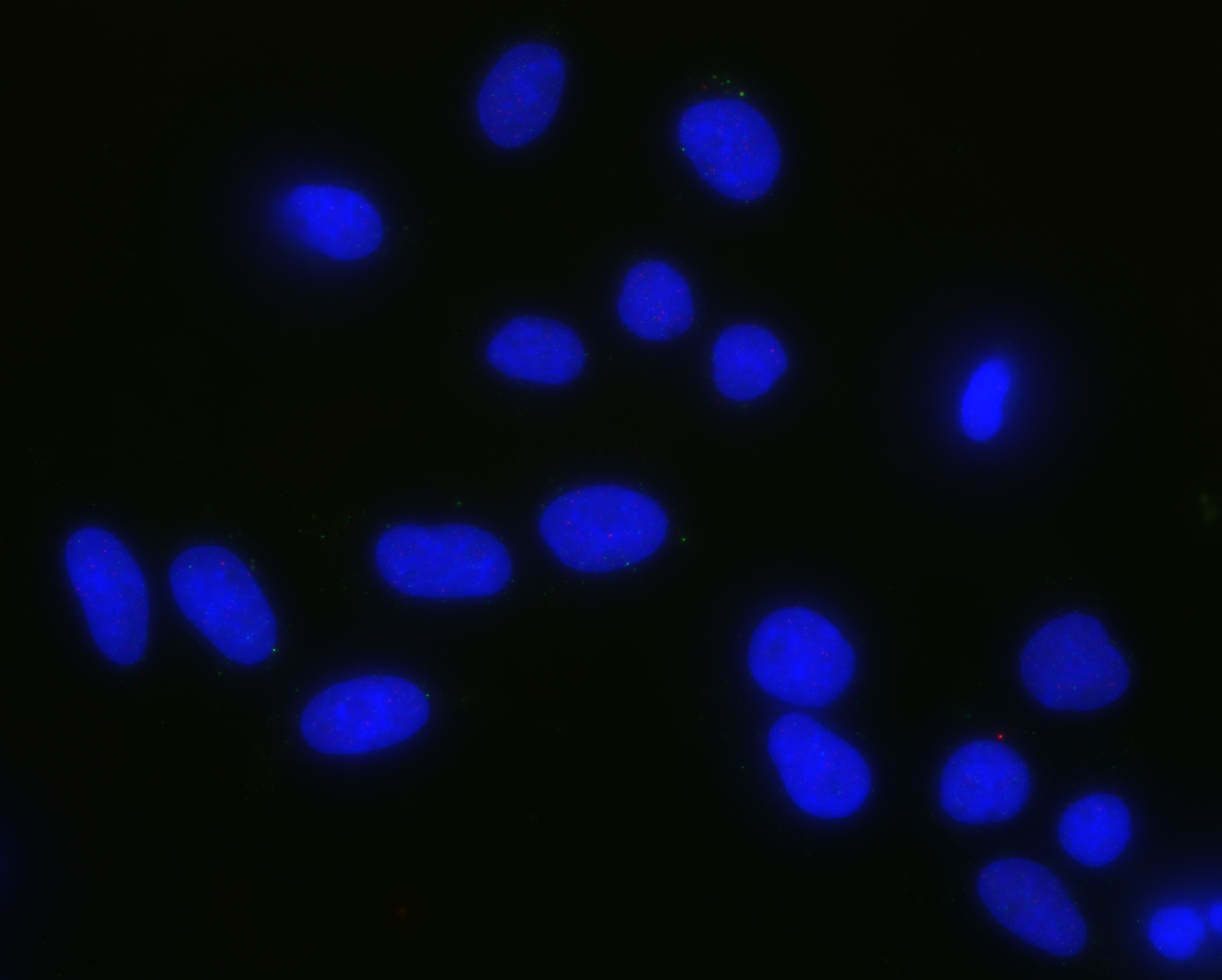

Supplement: Supplementary file 4 — Source data Fig. 3 [file 44319_2024_295_MOESM4_ESM.zip › Figure 3/3B/PC4+TRF2 image - Saos2 siCt.tif]

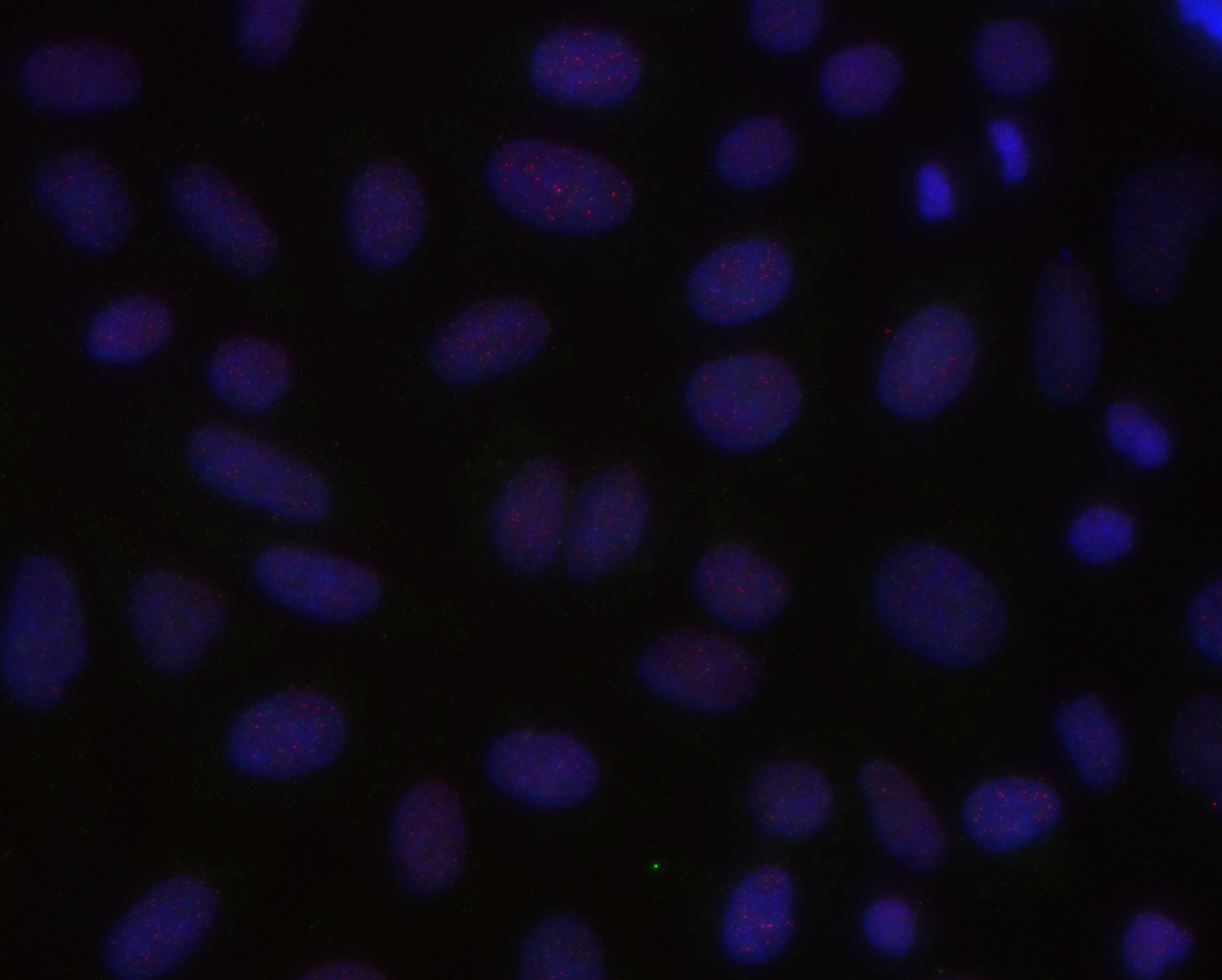

Supplement: Supplementary file 4 — Source data Fig. 3 [file 44319_2024_295_MOESM4_ESM.zip › Figure 3/3B/PC4+TRF2 image - HeLa siFM.tif]

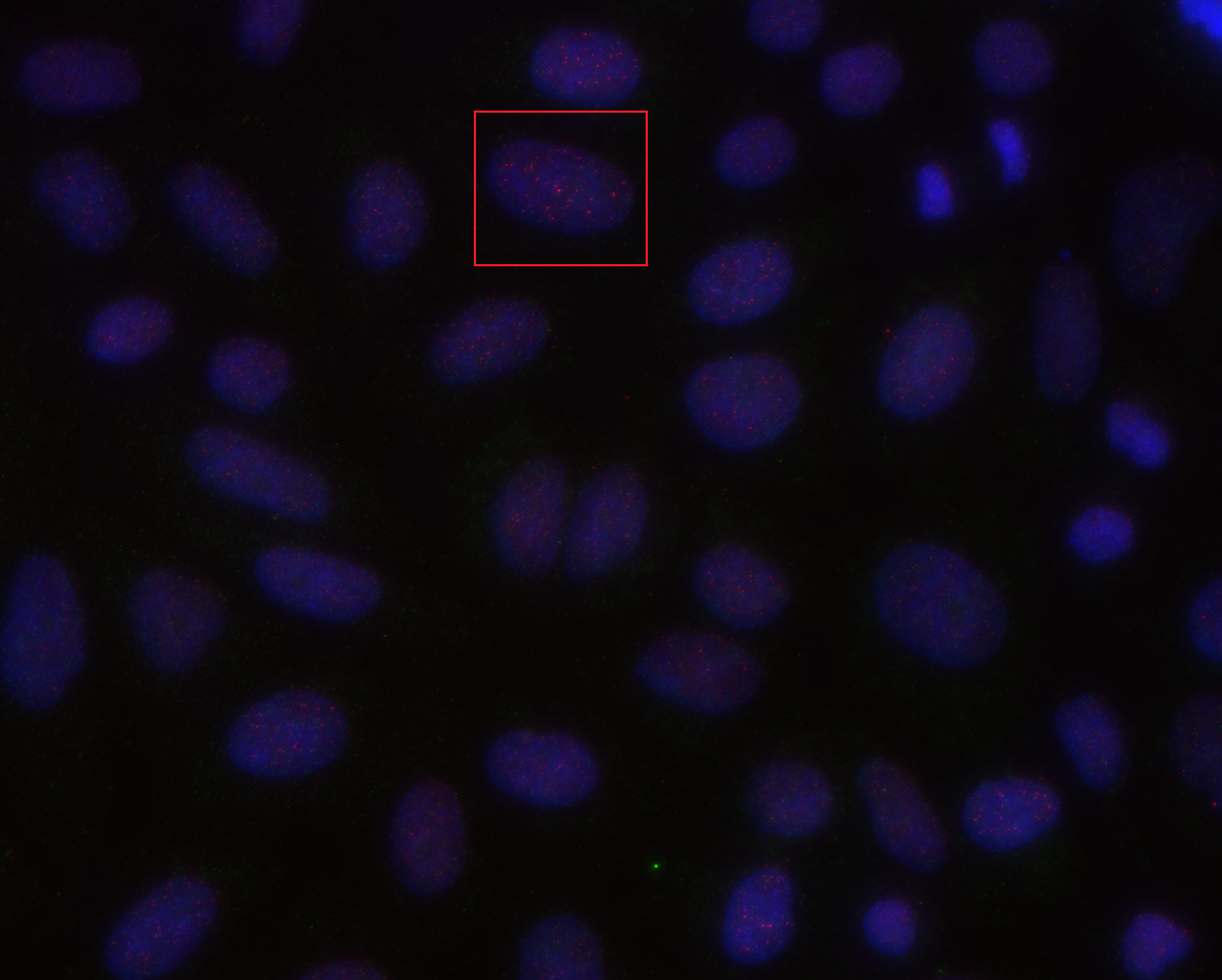

Supplement: Supplementary file 4 — Source data Fig. 3 [file 44319_2024_295_MOESM4_ESM.zip › Figure 3/3B/PC4+TRF2 image - representative nucleus - HeLa siFM.tif]

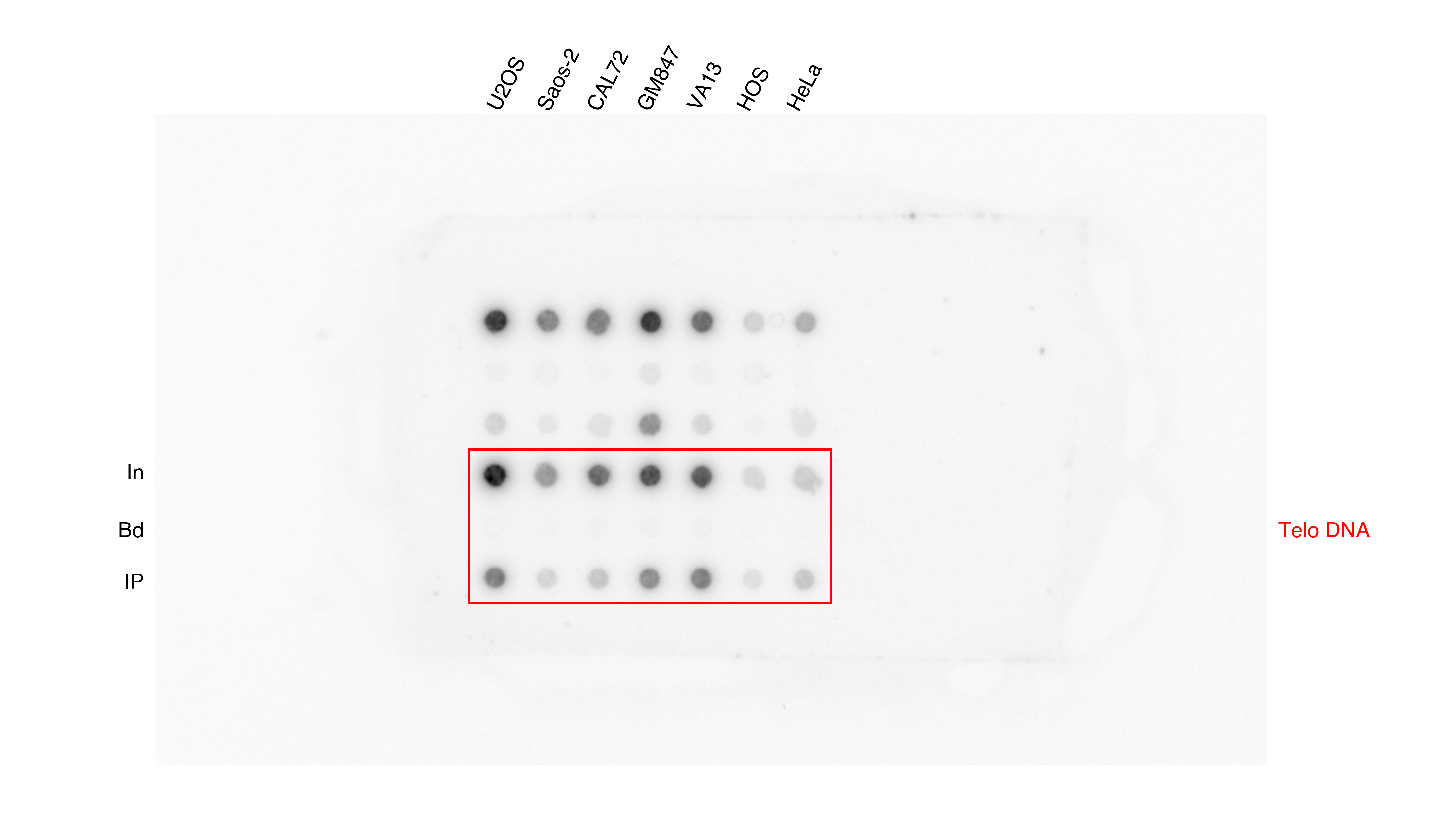

Supplement: Supplementary file 4 — Source data Fig. 3 [file 44319_2024_295_MOESM4_ESM.zip › Figure 3/3A/DotBlot-TeloDNA.tif]

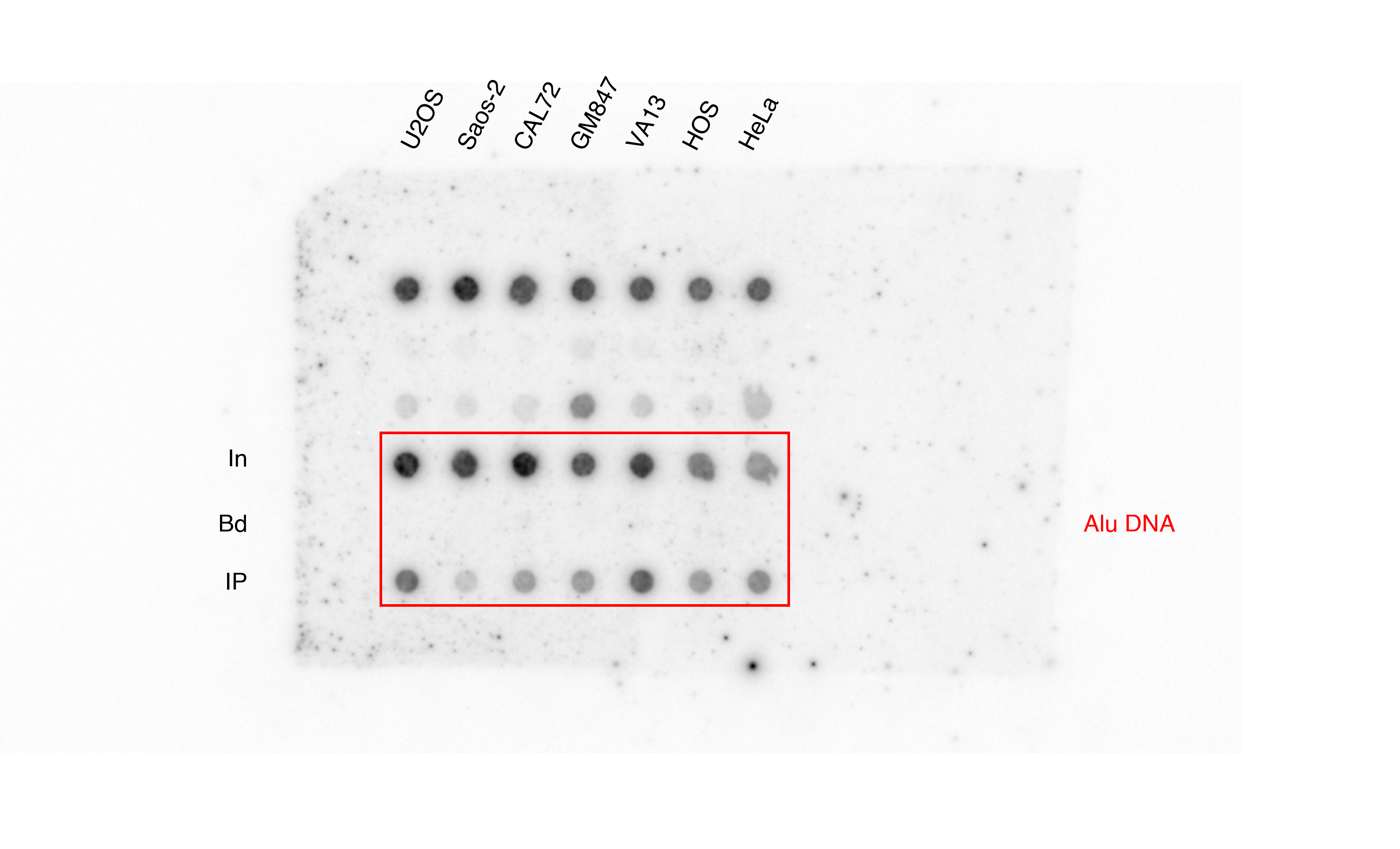

Supplement: Supplementary file 4 — Source data Fig. 3 [file 44319_2024_295_MOESM4_ESM.zip › Figure 3/3A/DotBlot-AluDNA.tif]

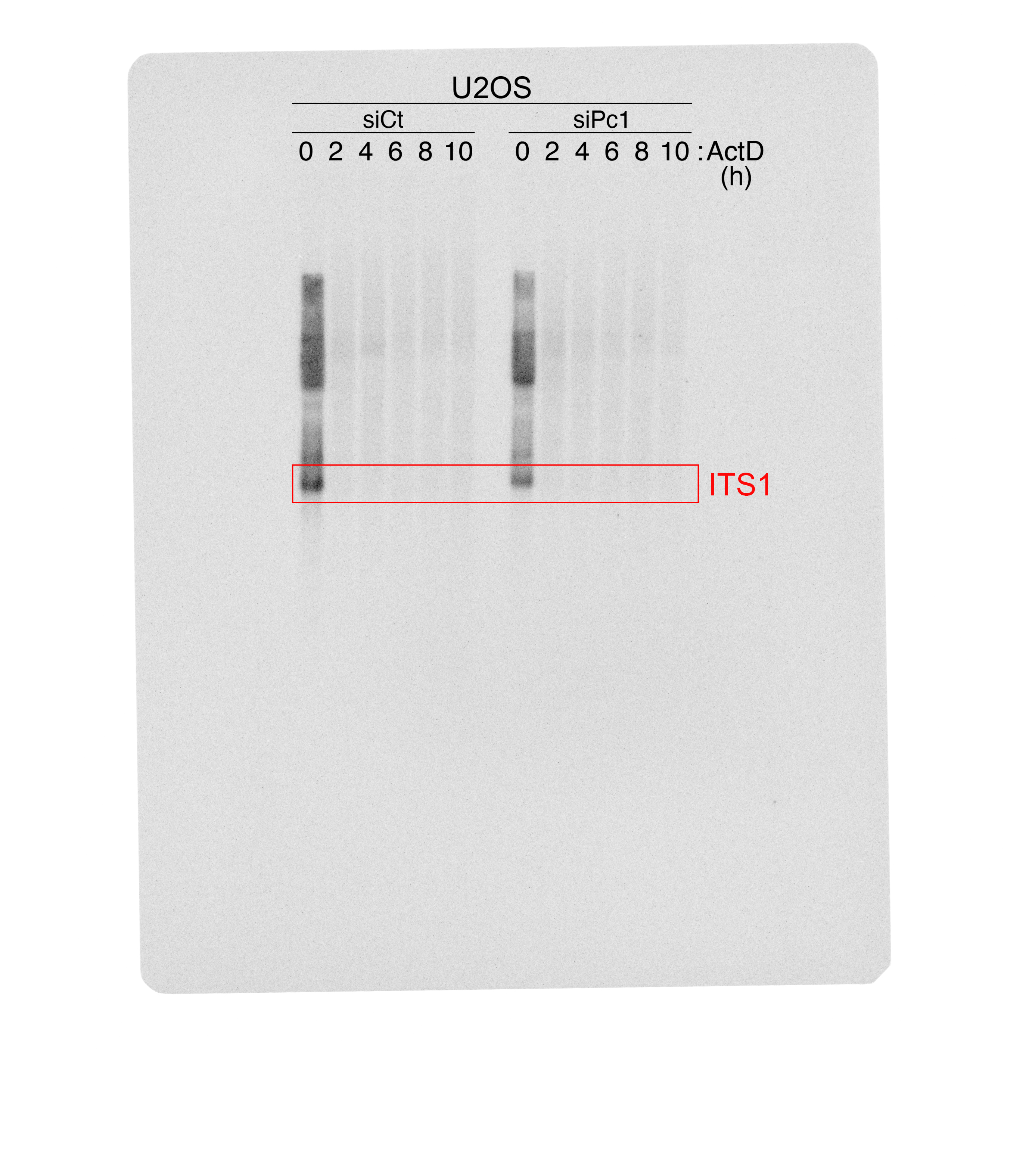

Supplement: Supplementary file 5 — Source data Fig. 4 [file 44319_2024_295_MOESM5_ESM.zip › Figure 4/4B/NorthernBlot-ITS1.tif]

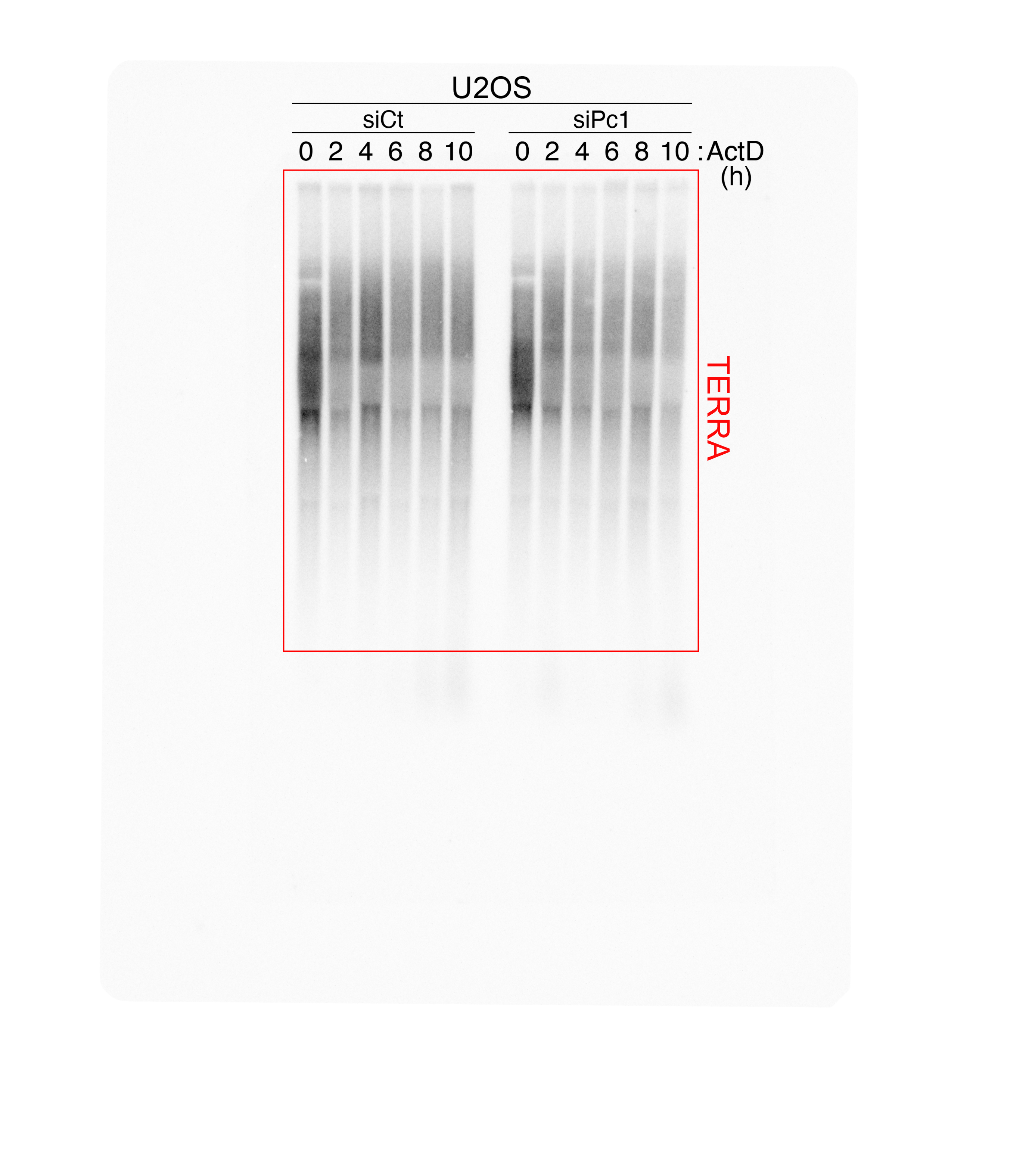

Supplement: Supplementary file 5 — Source data Fig. 4 [file 44319_2024_295_MOESM5_ESM.zip › Figure 4/4B/NorthernBlot-TERRA.tif]

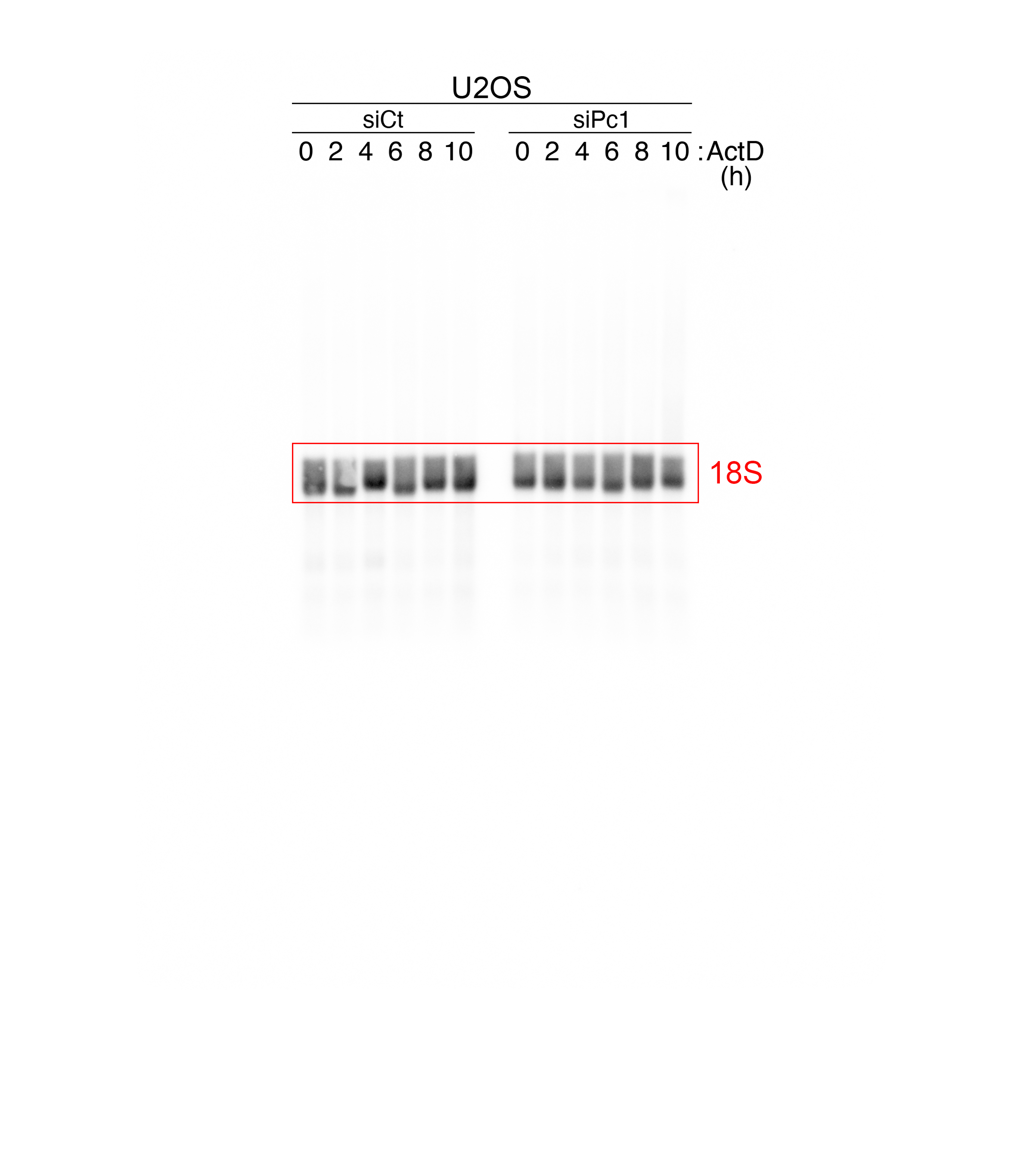

Supplement: Supplementary file 5 — Source data Fig. 4 [file 44319_2024_295_MOESM5_ESM.zip › Figure 4/4B/NorthernBlot-18S.tif]

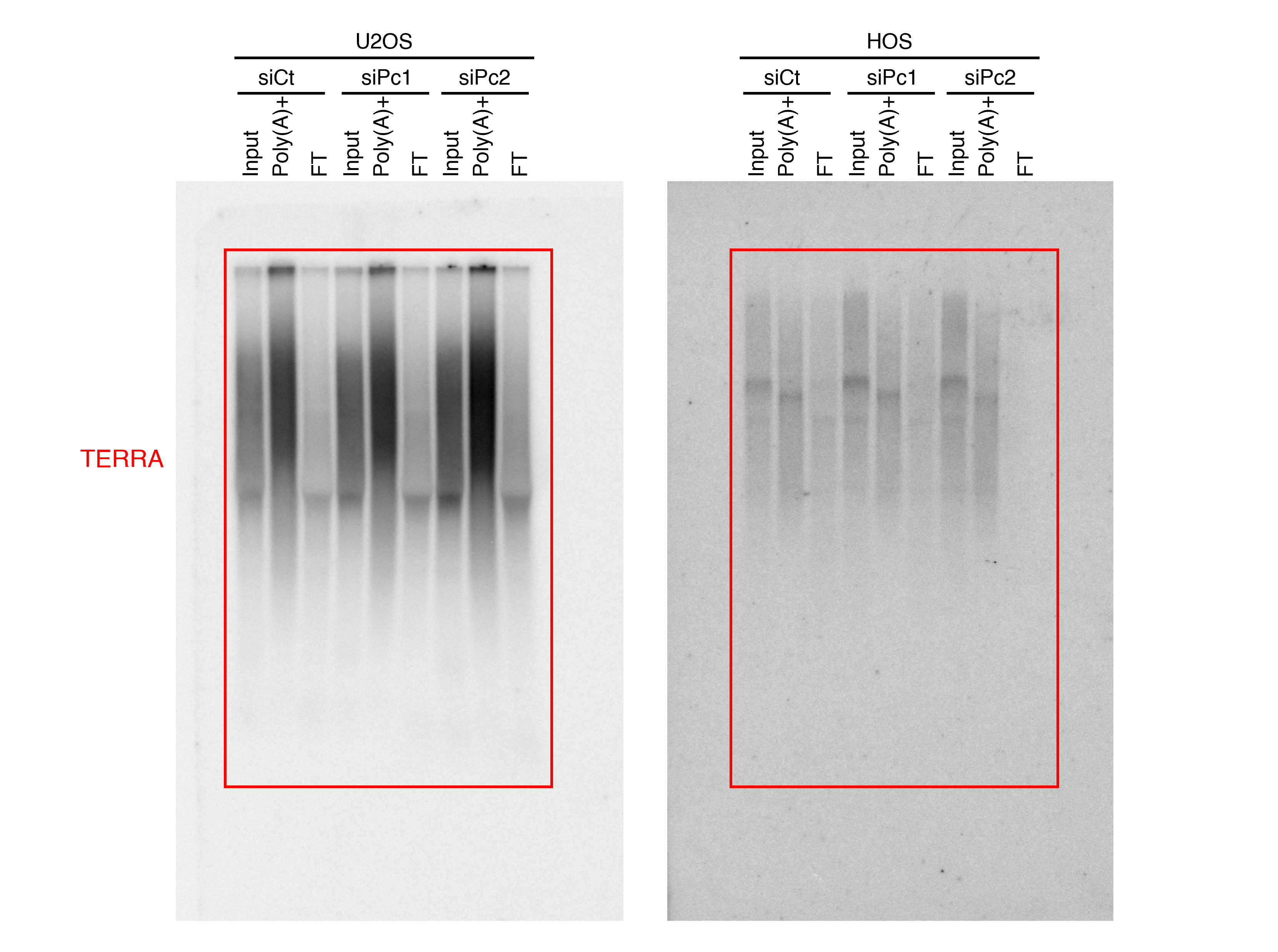

Supplement: Supplementary file 5 — Source data Fig. 4 [file 44319_2024_295_MOESM5_ESM.zip › Figure 4/4C/NorthernBlot-TERRA.tif]

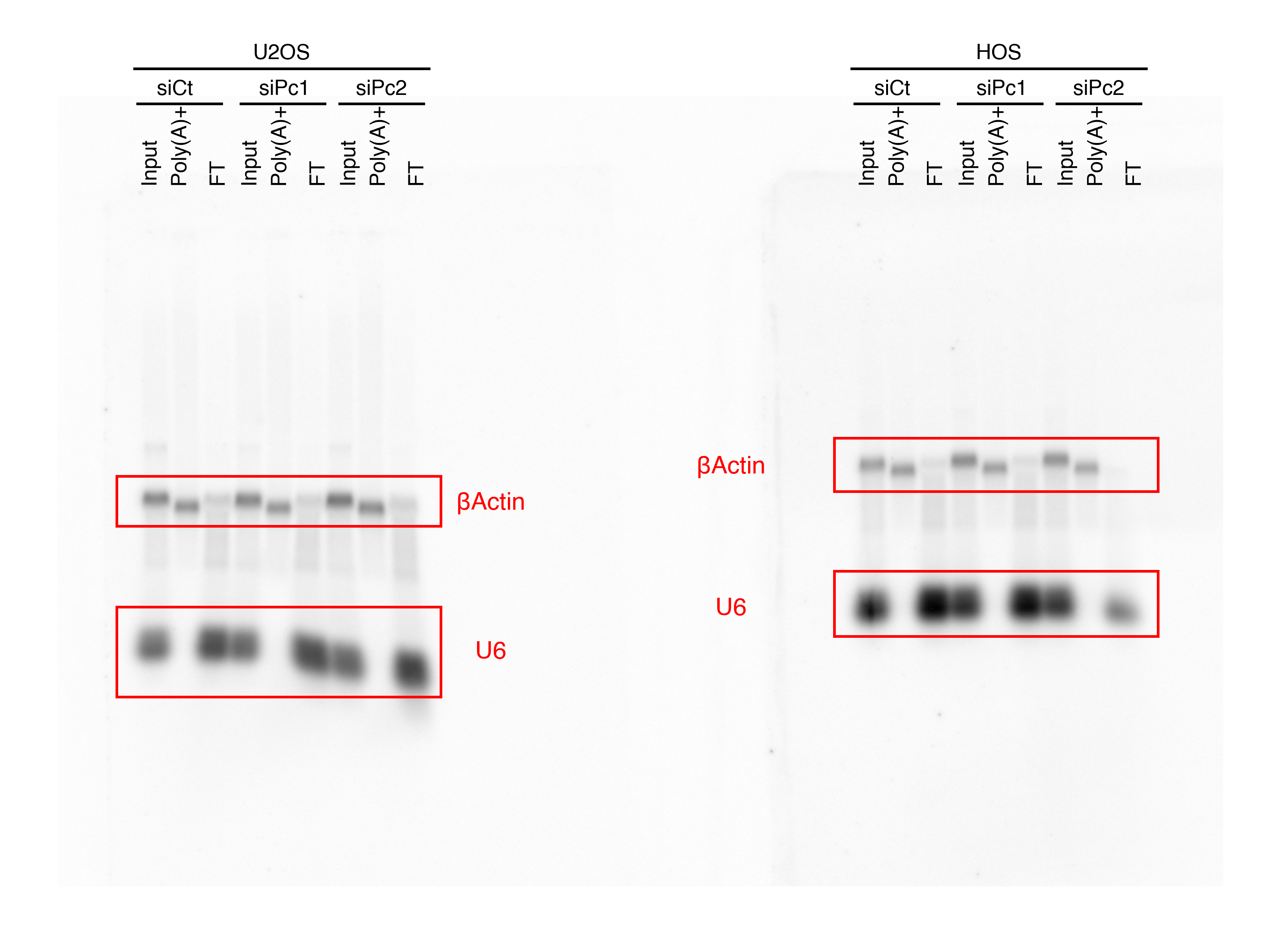

Supplement: Supplementary file 5 — Source data Fig. 4 [file 44319_2024_295_MOESM5_ESM.zip › Figure 4/4C/NorthernBlot-BetaActin-U6.tif]

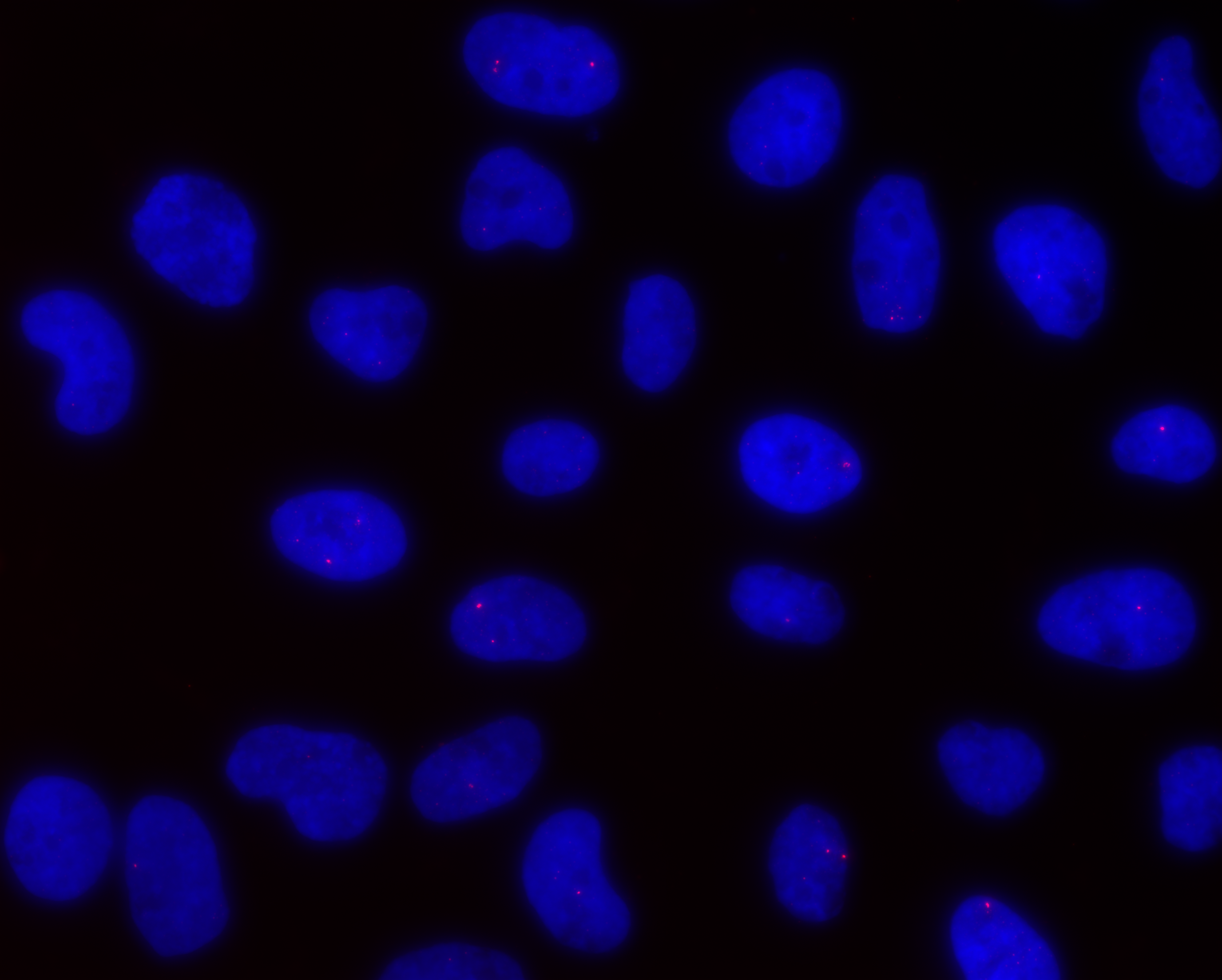

Supplement: Supplementary file 5 — Source data Fig. 4 [file 44319_2024_295_MOESM5_ESM.zip › Figure 4/4D/RNA FISH image - U2OS siPc1.tif]

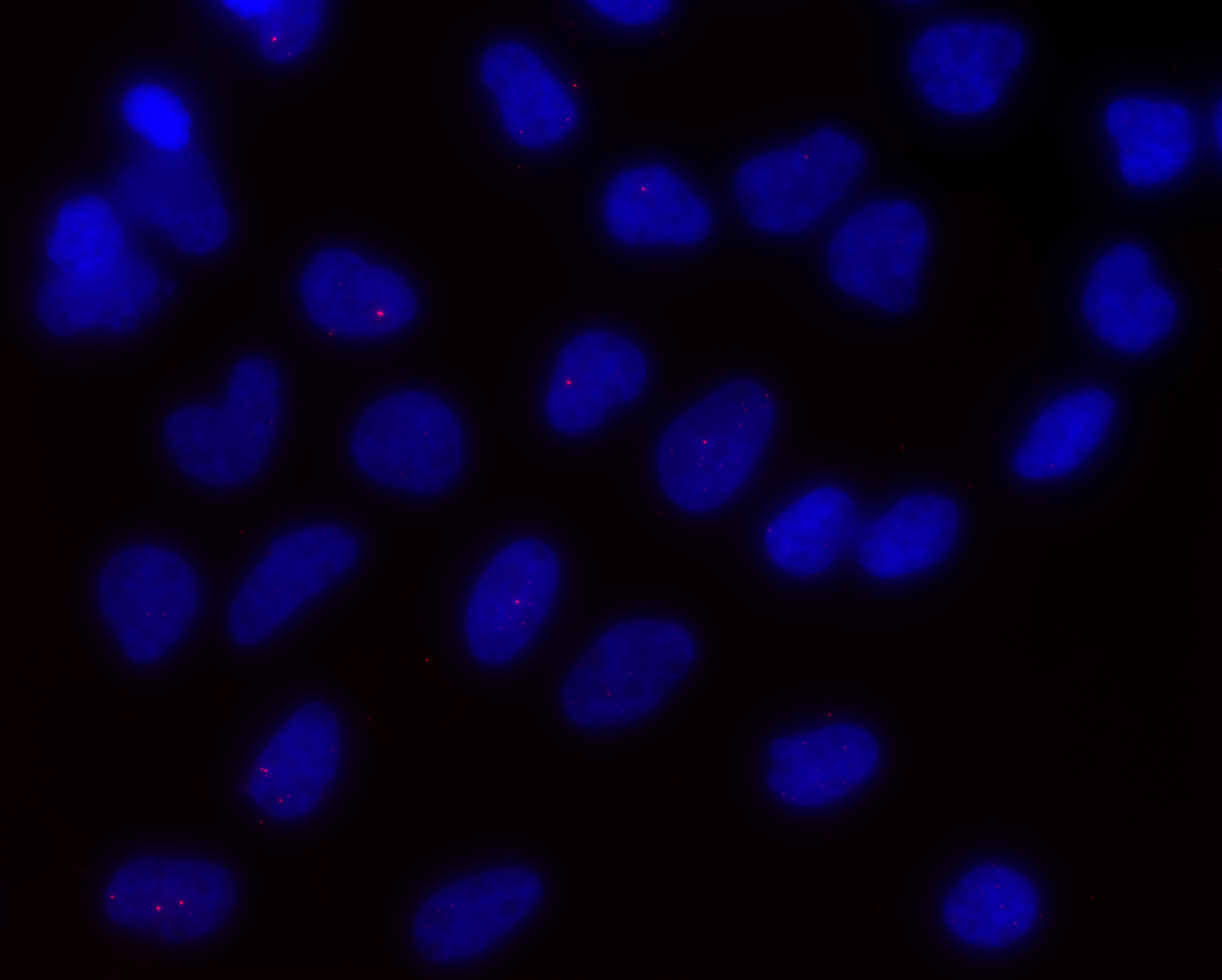

Supplement: Supplementary file 5 — Source data Fig. 4 [file 44319_2024_295_MOESM5_ESM.zip › Figure 4/4D/RNA FISH image - U2OS siCtr.tif]

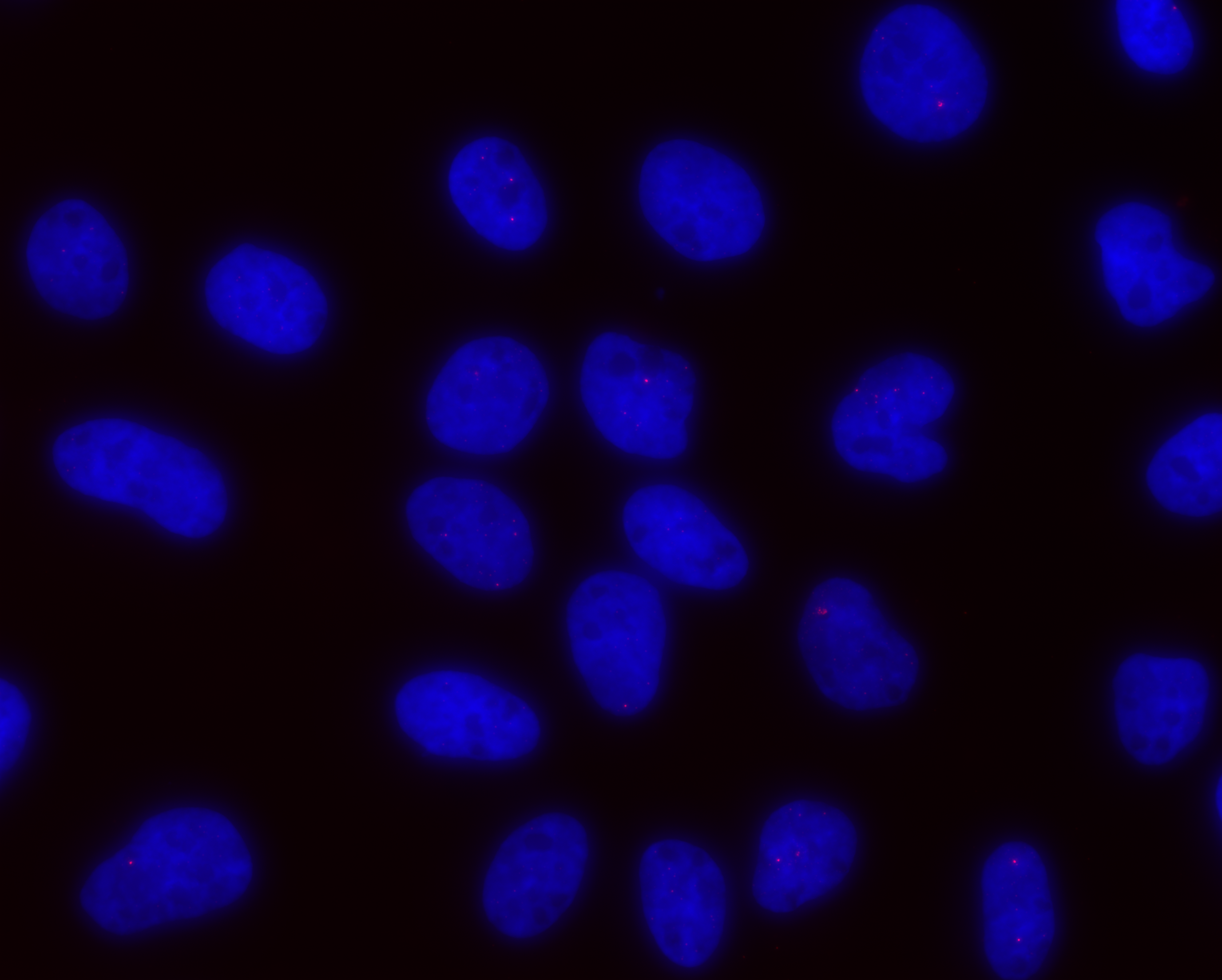

Supplement: Supplementary file 5 — Source data Fig. 4 [file 44319_2024_295_MOESM5_ESM.zip › Figure 4/4D/RNA FISH image - U2OS siPc2.tif]

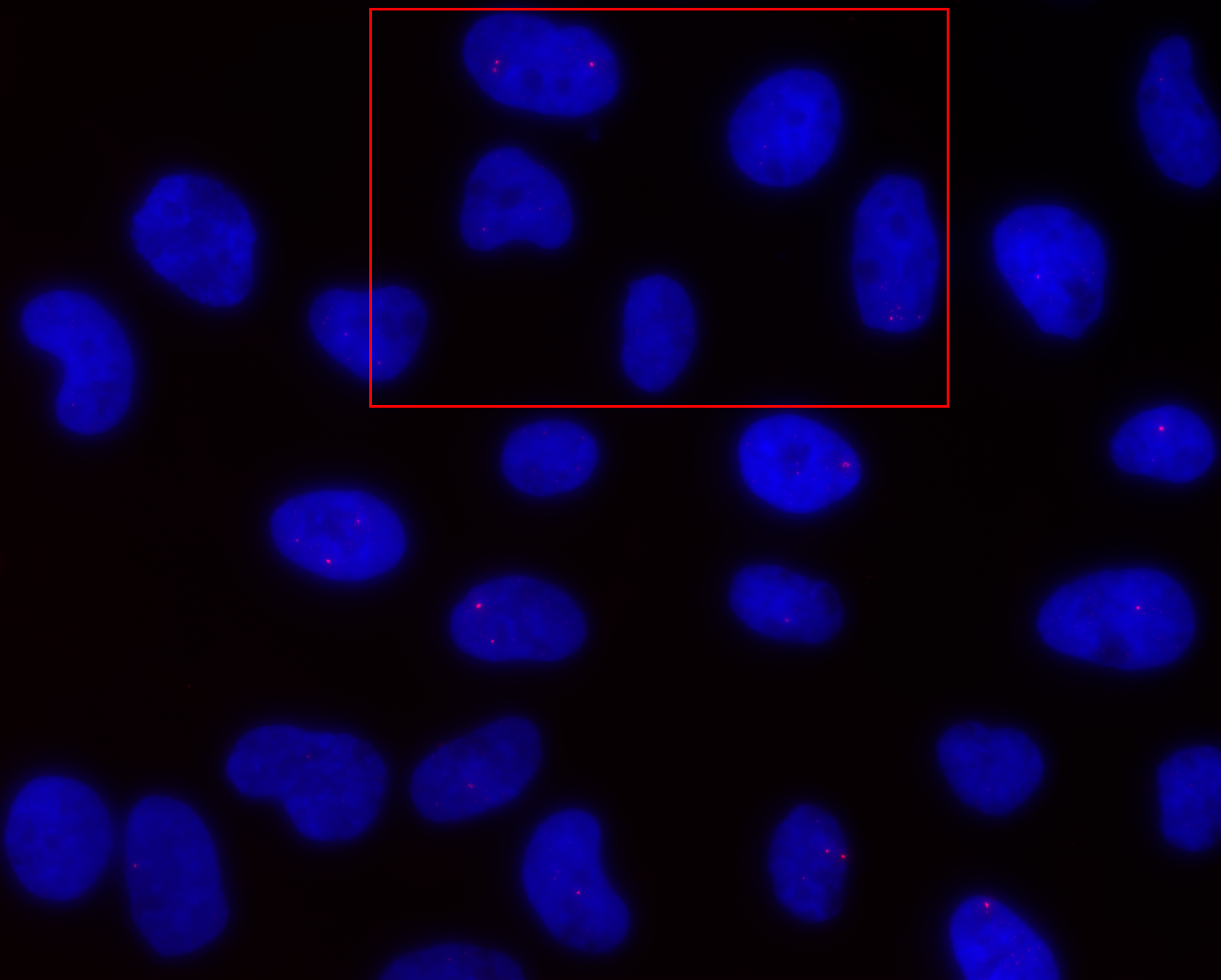

Supplement: Supplementary file 5 — Source data Fig. 4 [file 44319_2024_295_MOESM5_ESM.zip › Figure 4/4D/RNA FISH image - representative nuclei - U2OS siPc1.tif]

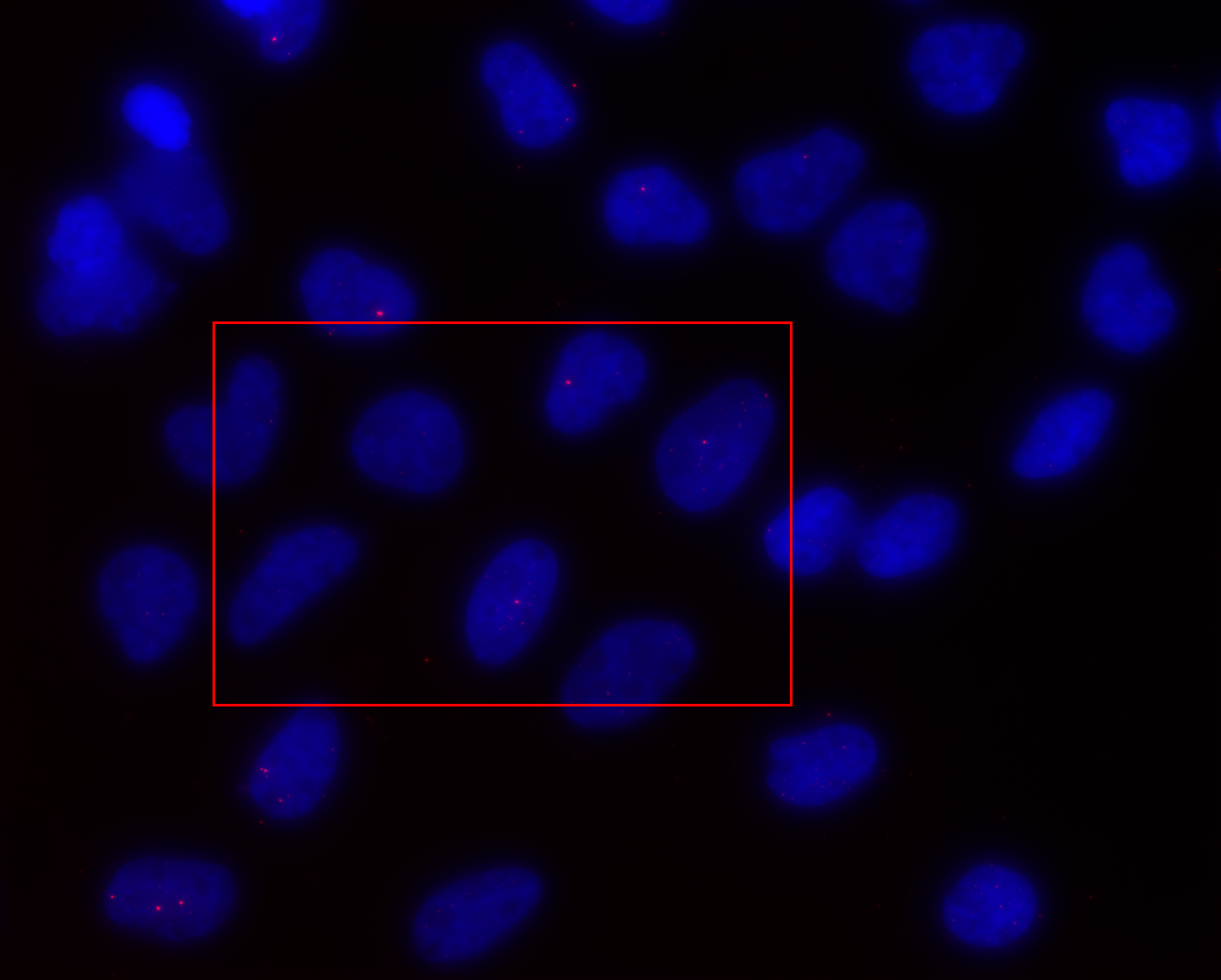

Supplement: Supplementary file 5 — Source data Fig. 4 [file 44319_2024_295_MOESM5_ESM.zip › Figure 4/4D/RNA FISH image - representative nuclei - U2OS siCtr.tif]

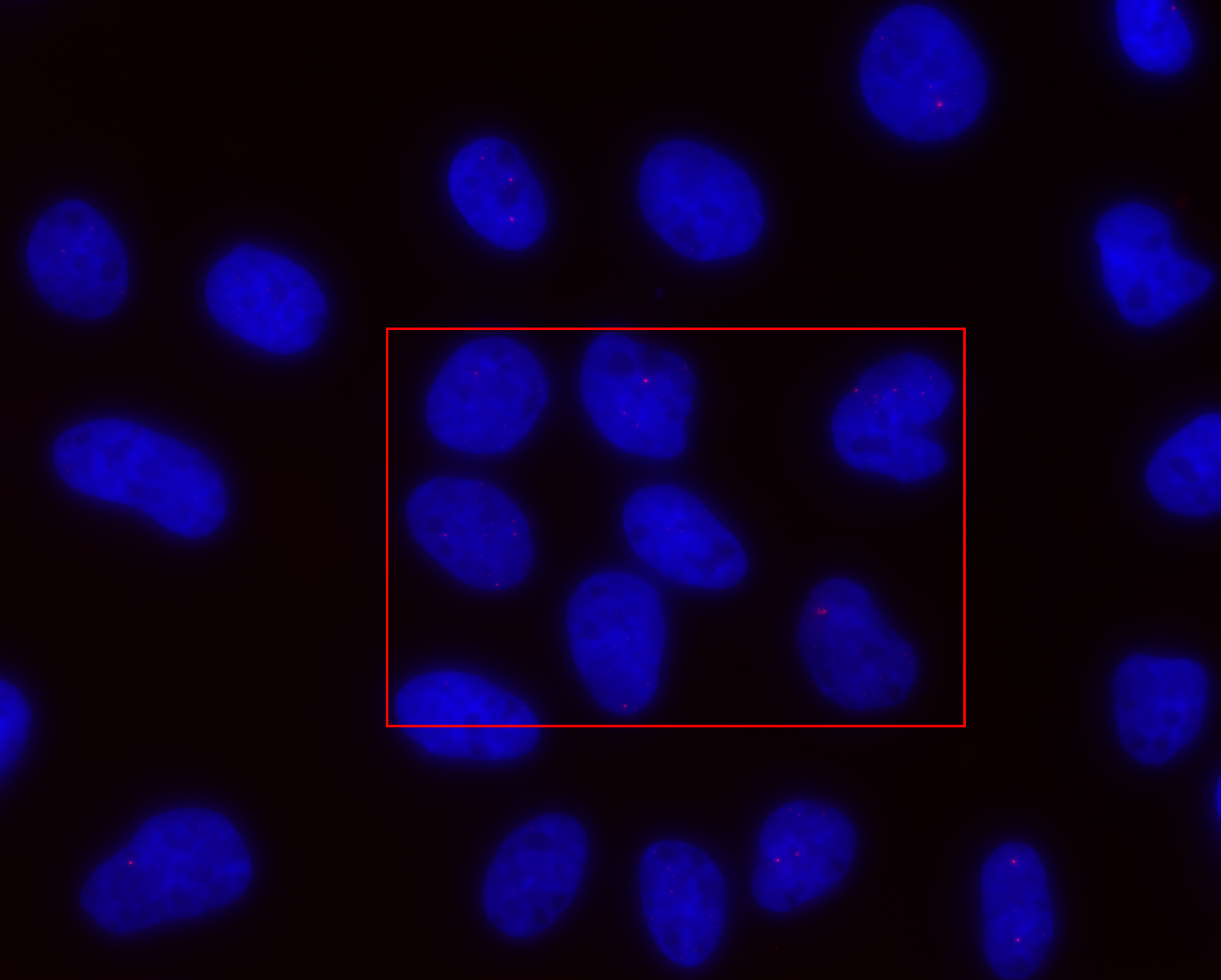

Supplement: Supplementary file 5 — Source data Fig. 4 [file 44319_2024_295_MOESM5_ESM.zip › Figure 4/4D/RNA FISH image - representative nuclei - U2OS siPc2.tif]

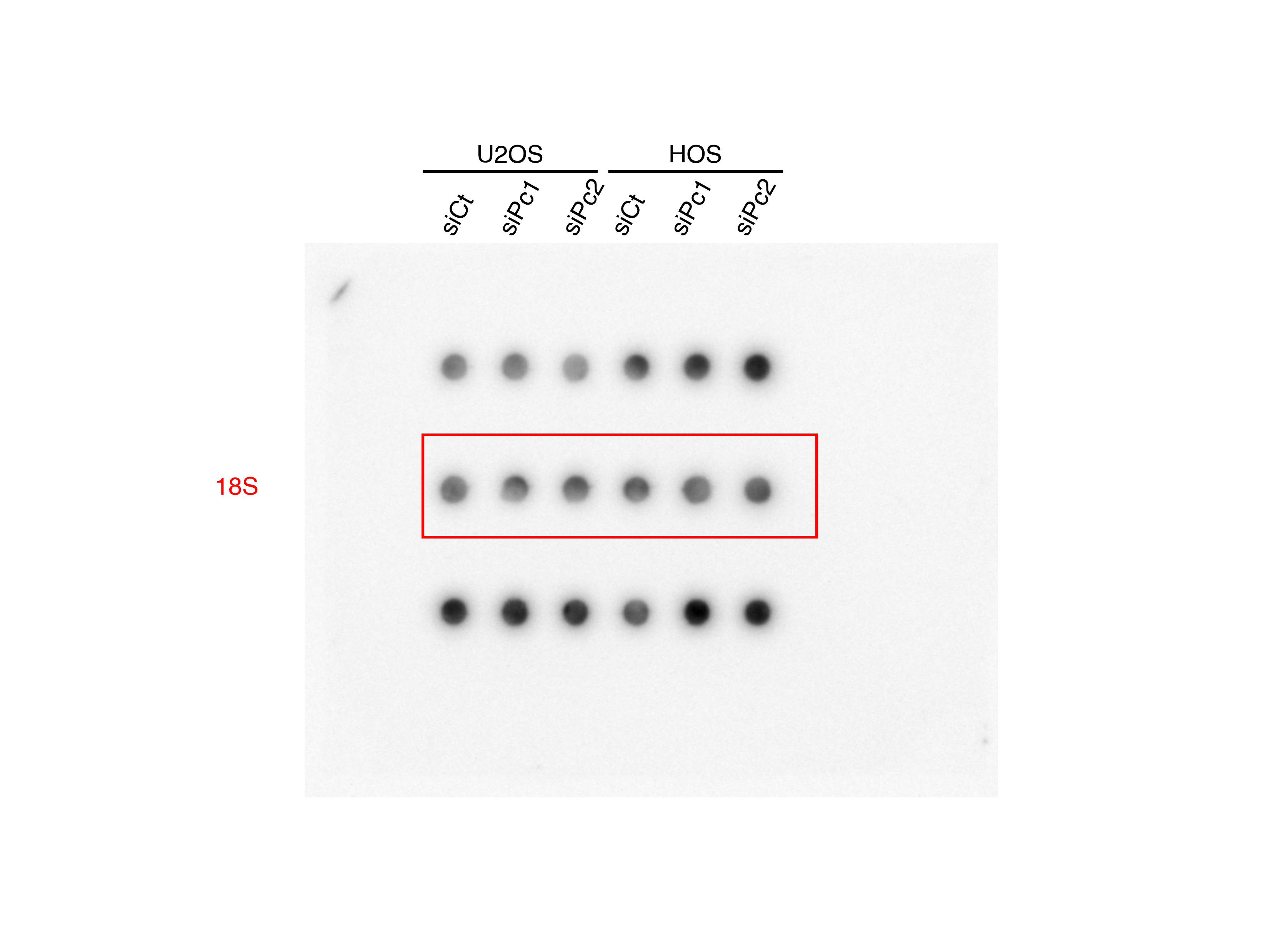

Supplement: Supplementary file 5 — Source data Fig. 4 [file 44319_2024_295_MOESM5_ESM.zip › Figure 4/4A/DotBlot-18S.tif]

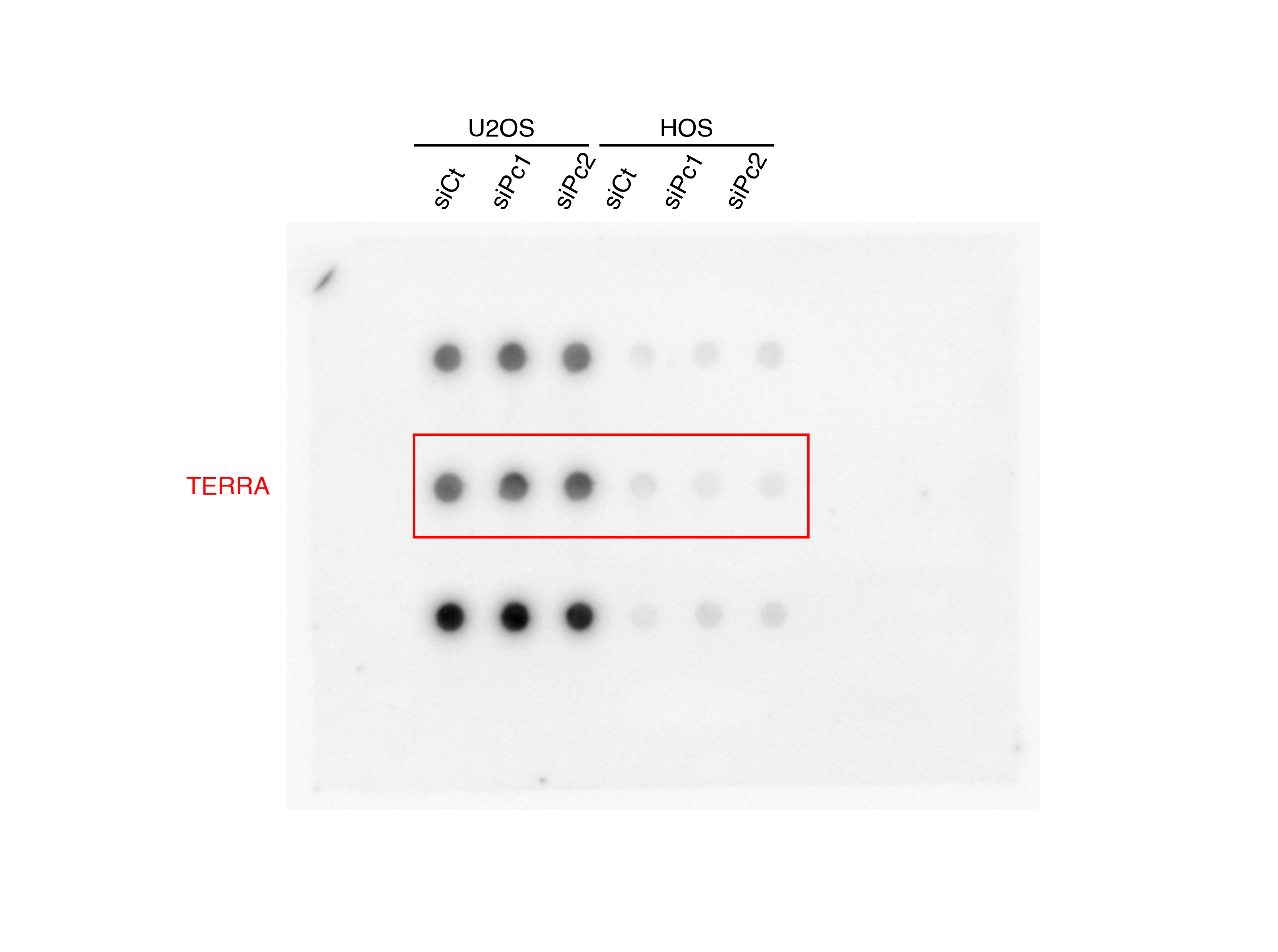

Supplement: Supplementary file 5 — Source data Fig. 4 [file 44319_2024_295_MOESM5_ESM.zip › Figure 4/4A/DotBlot-TERRA.tif]

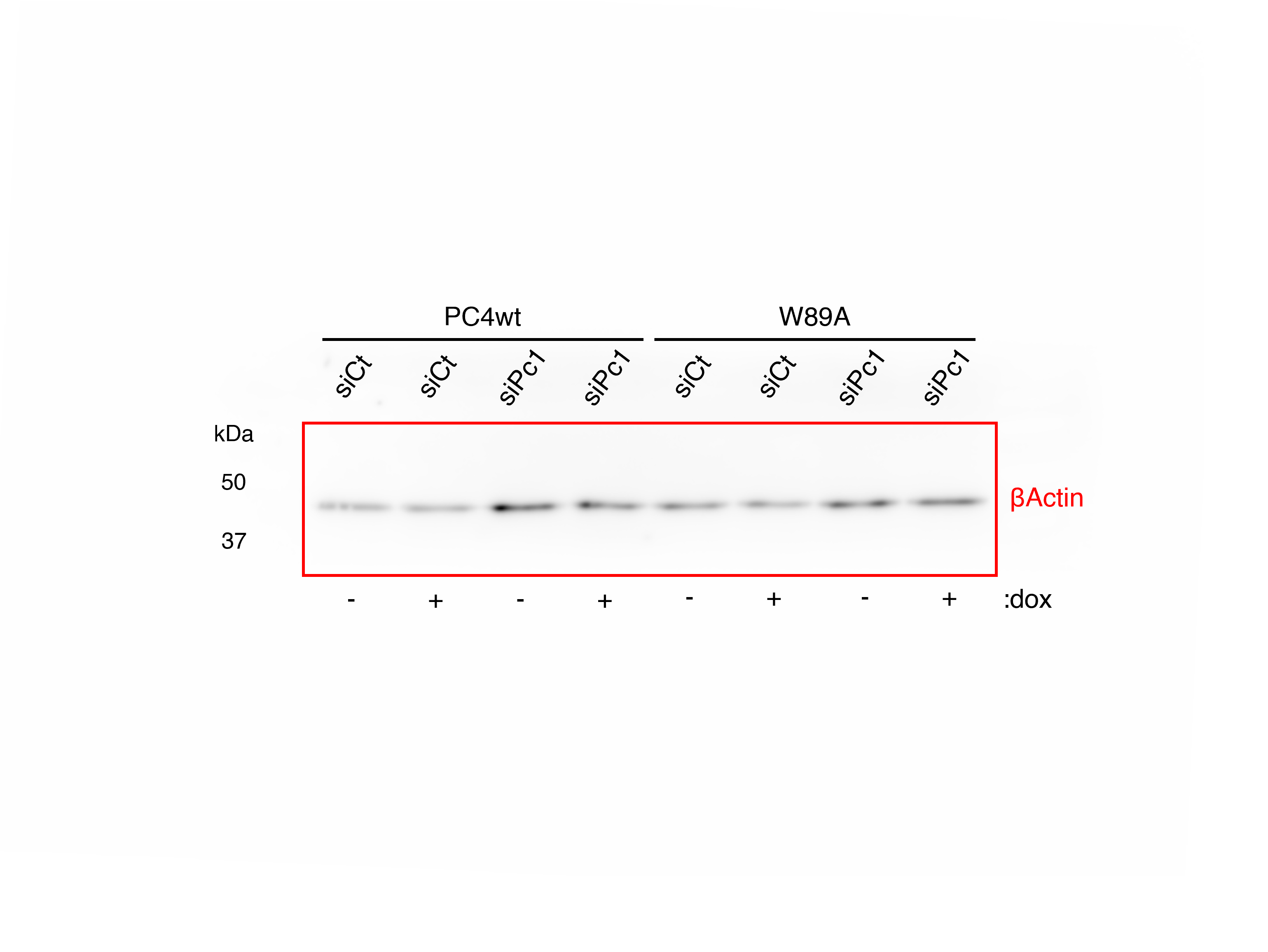

Supplement: Supplementary file 6 — Source data Fig. 5 [file 44319_2024_295_MOESM6_ESM.zip › Figure 5/5A/WesternBlot-BActin.tif]

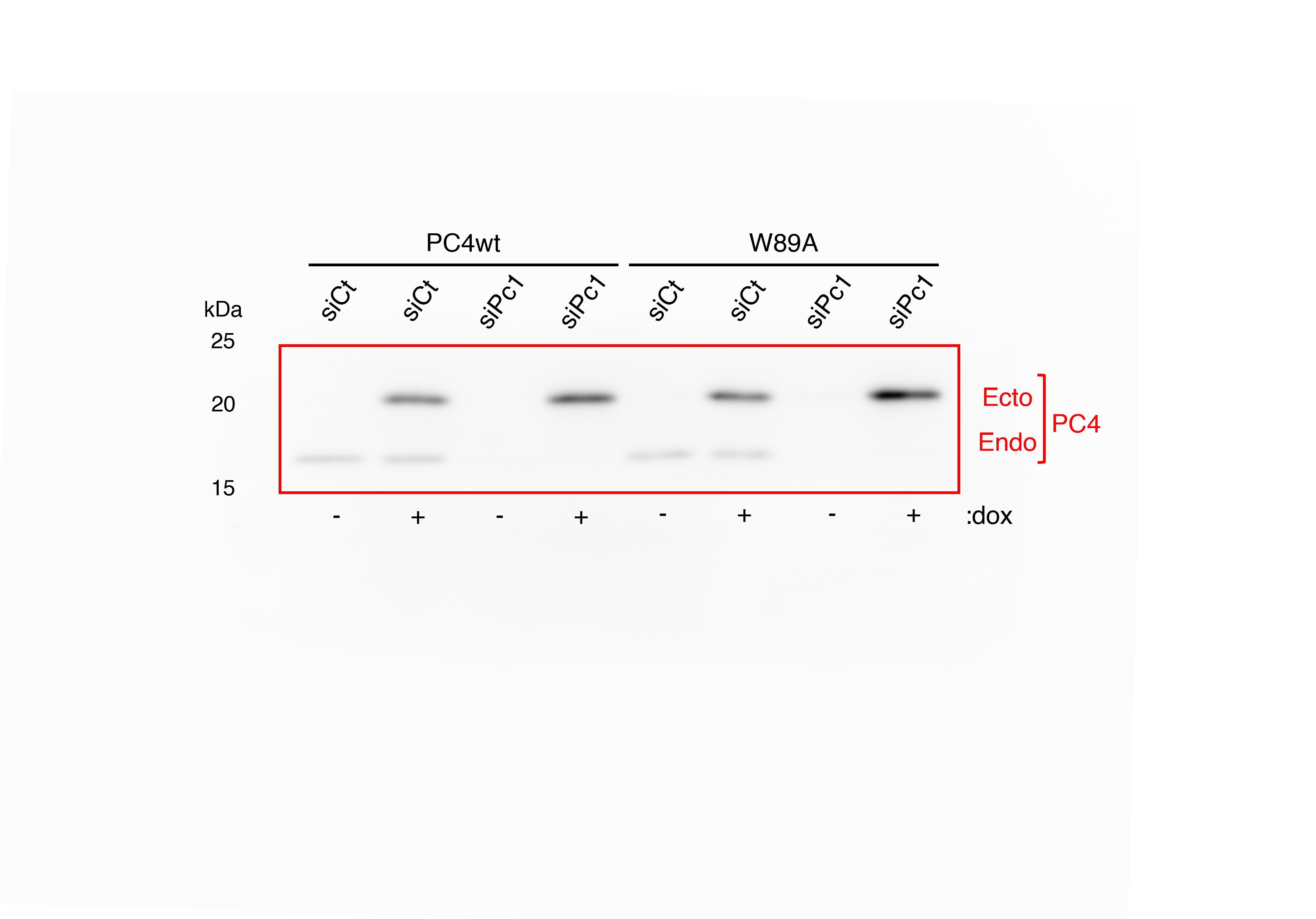

Supplement: Supplementary file 6 — Source data Fig. 5 [file 44319_2024_295_MOESM6_ESM.zip › Figure 5/5A/WesternBlot-PC4.tif]

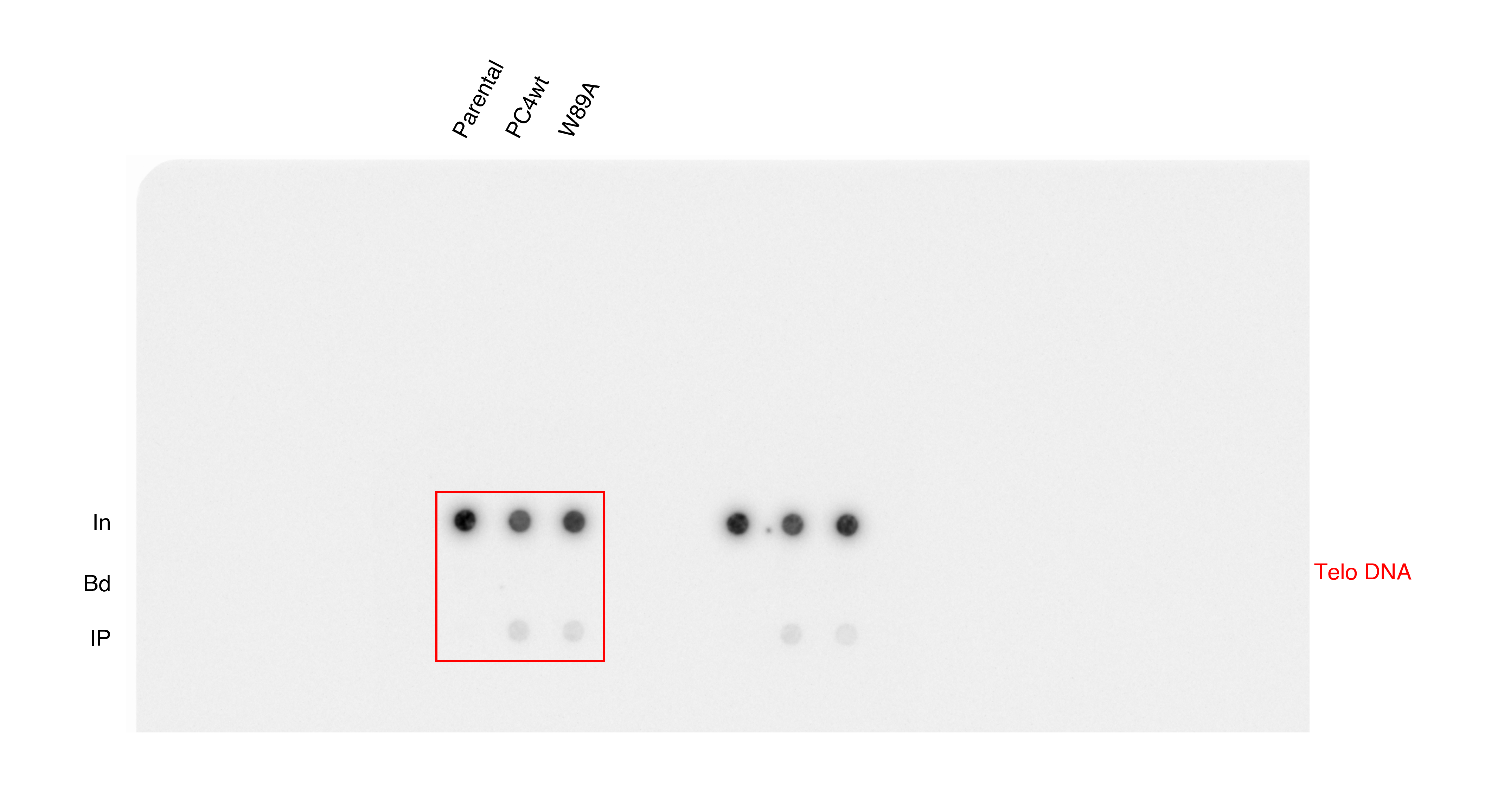

Supplement: Supplementary file 6 — Source data Fig. 5 [file 44319_2024_295_MOESM6_ESM.zip › Figure 5/5C/DotBlot-TeloDNA.tif]

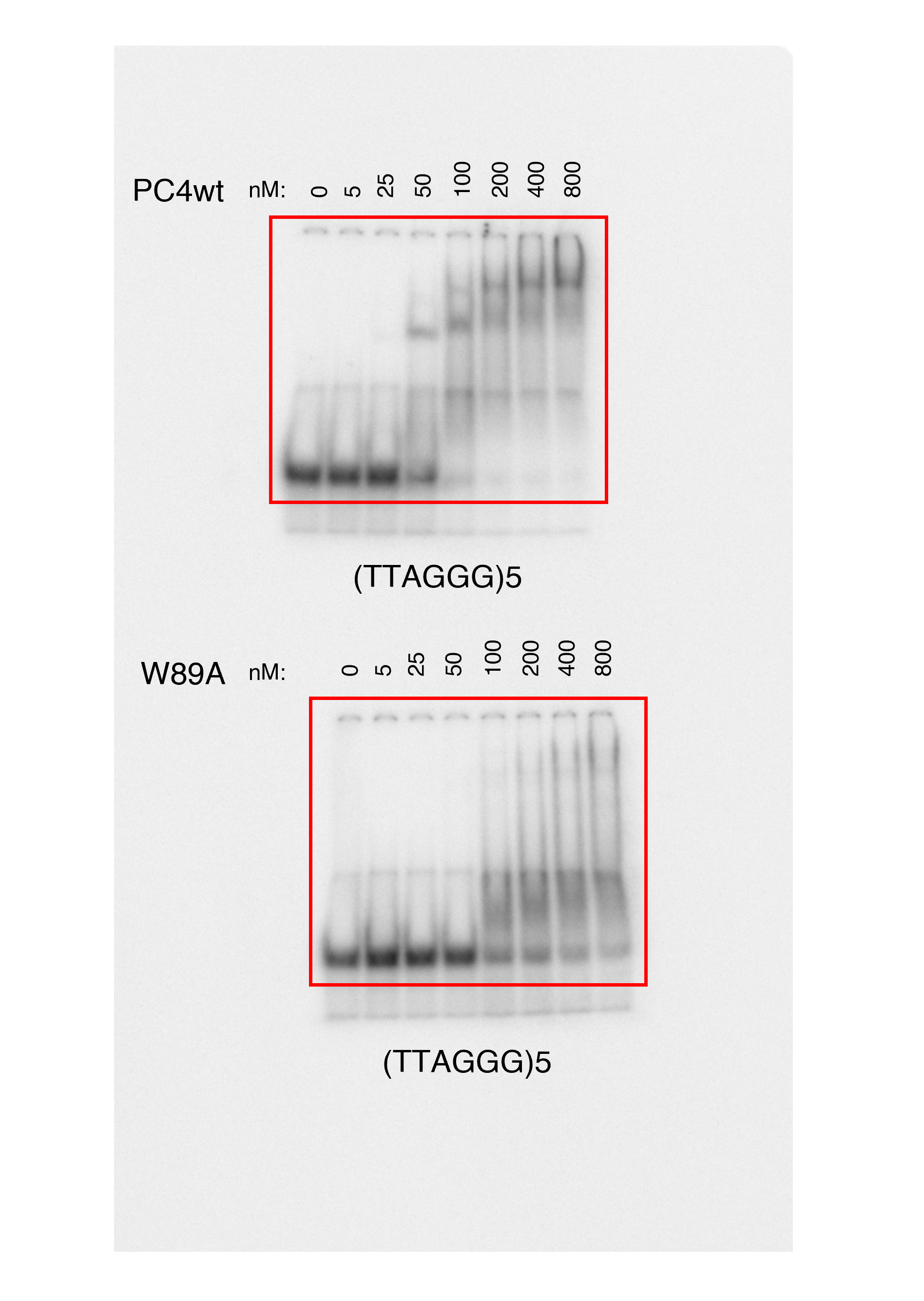

Supplement: Supplementary file 6 — Source data Fig. 5 [file 44319_2024_295_MOESM6_ESM.zip › Figure 5/5D/EMSAs-TTAGGG5.tif]

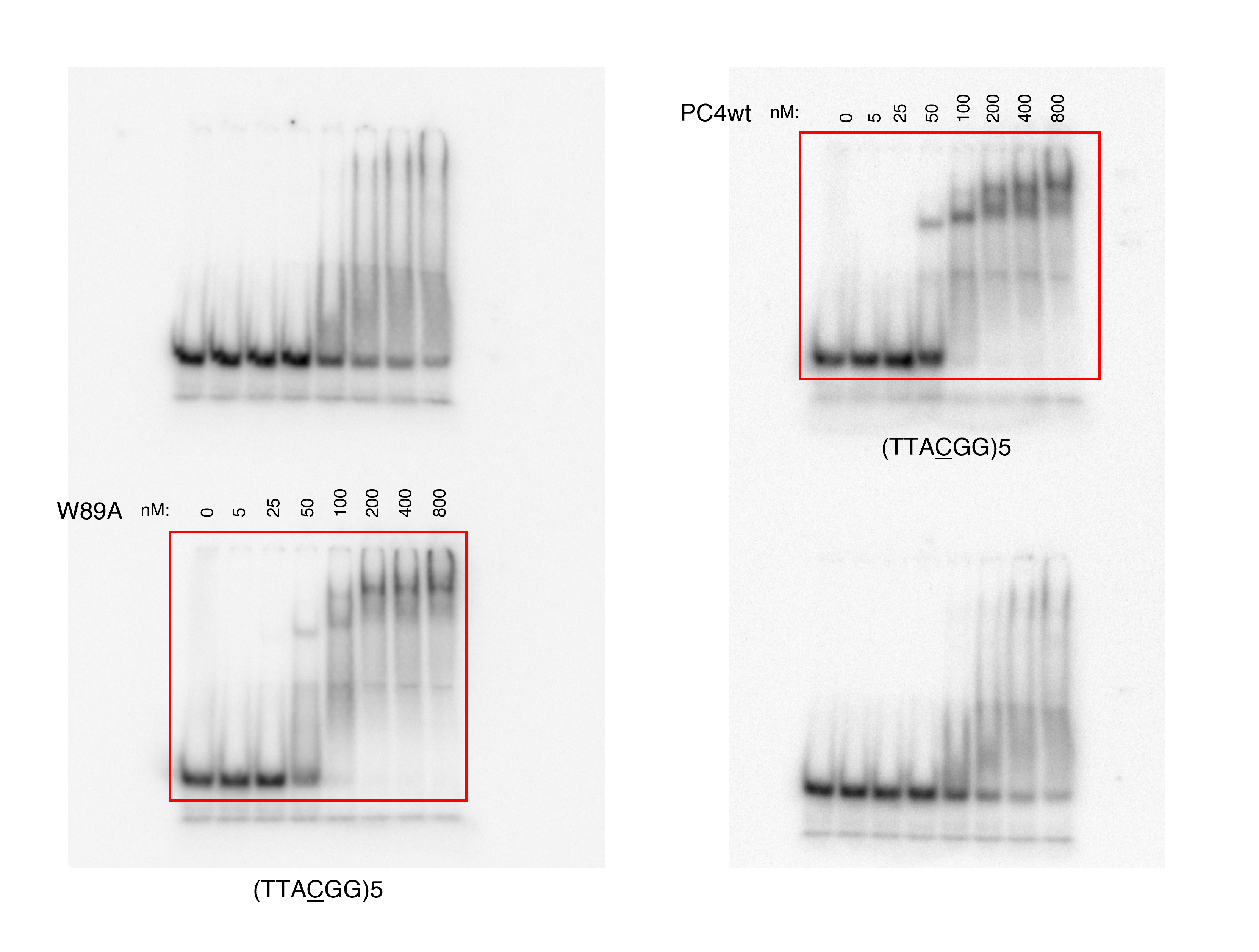

Supplement: Supplementary file 6 — Source data Fig. 5 [file 44319_2024_295_MOESM6_ESM.zip › Figure 5/5D/EMSAs-TTACGG5.tif]

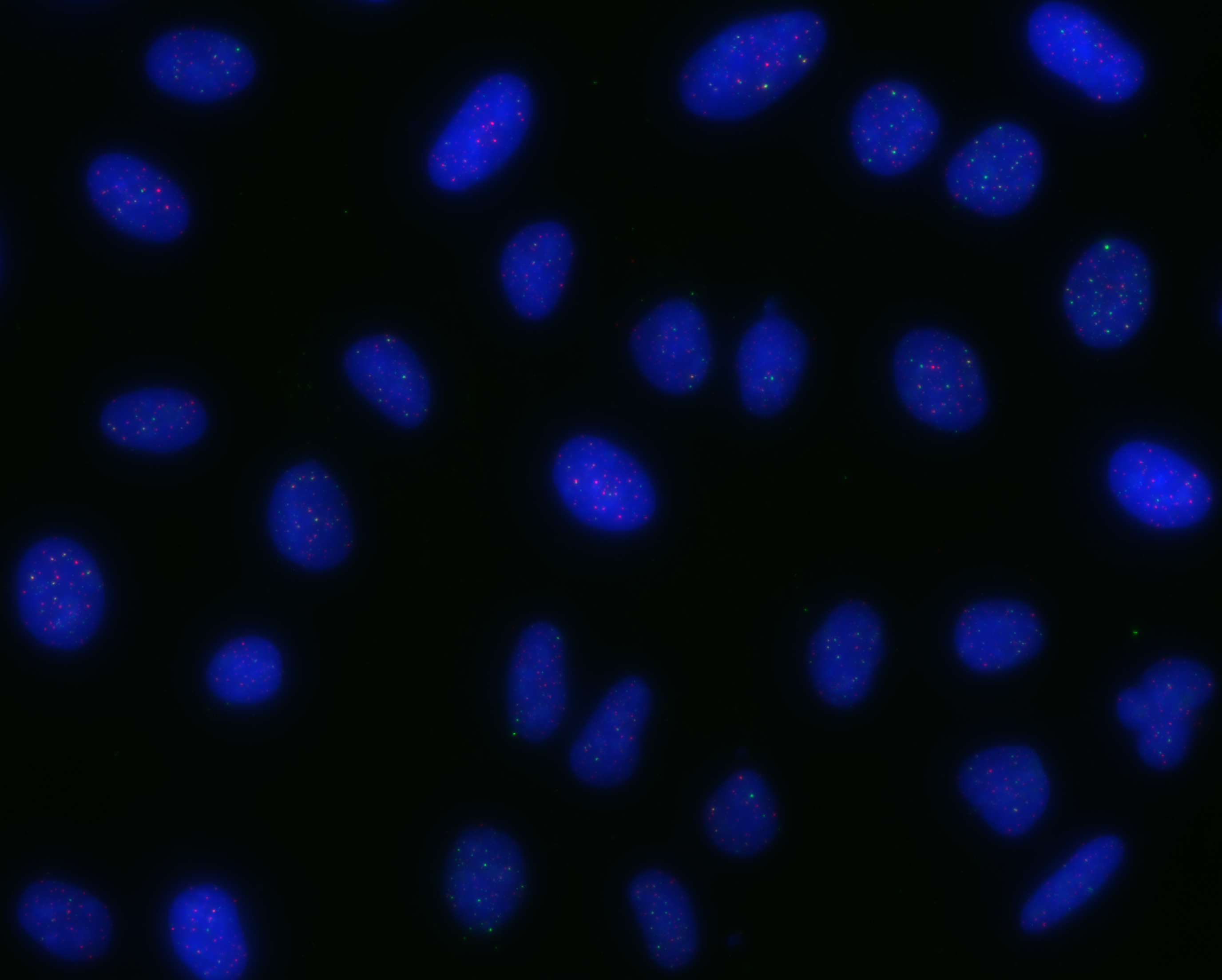

Supplement: Supplementary file 6 — Source data Fig. 5 [file 44319_2024_295_MOESM6_ESM.zip › Figure 5/5B/APBs image - W89A siCt +dox.tif]

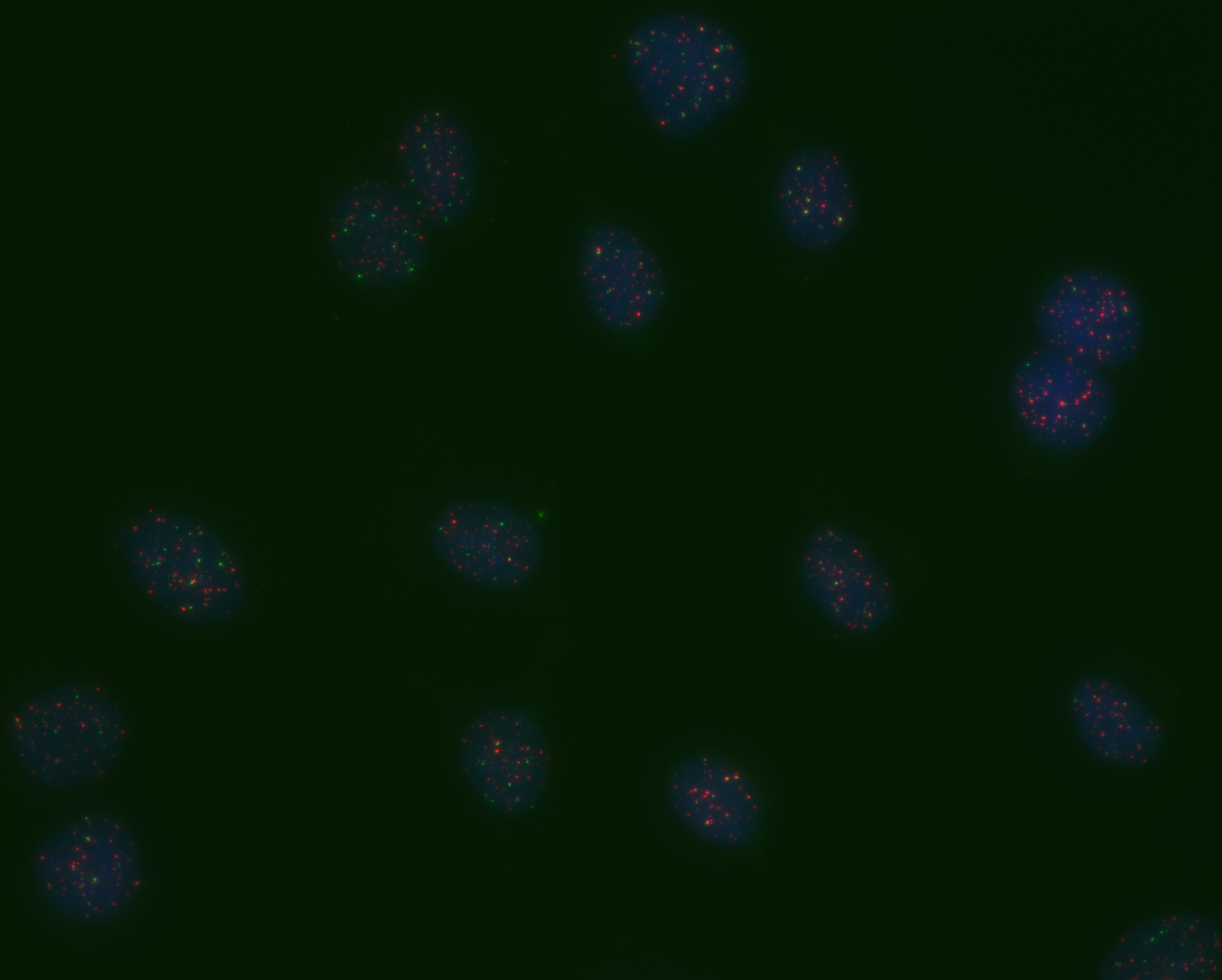

Supplement: Supplementary file 6 — Source data Fig. 5 [file 44319_2024_295_MOESM6_ESM.zip › Figure 5/5B/APBs image - PC4wt siPc1 +dox.tif]

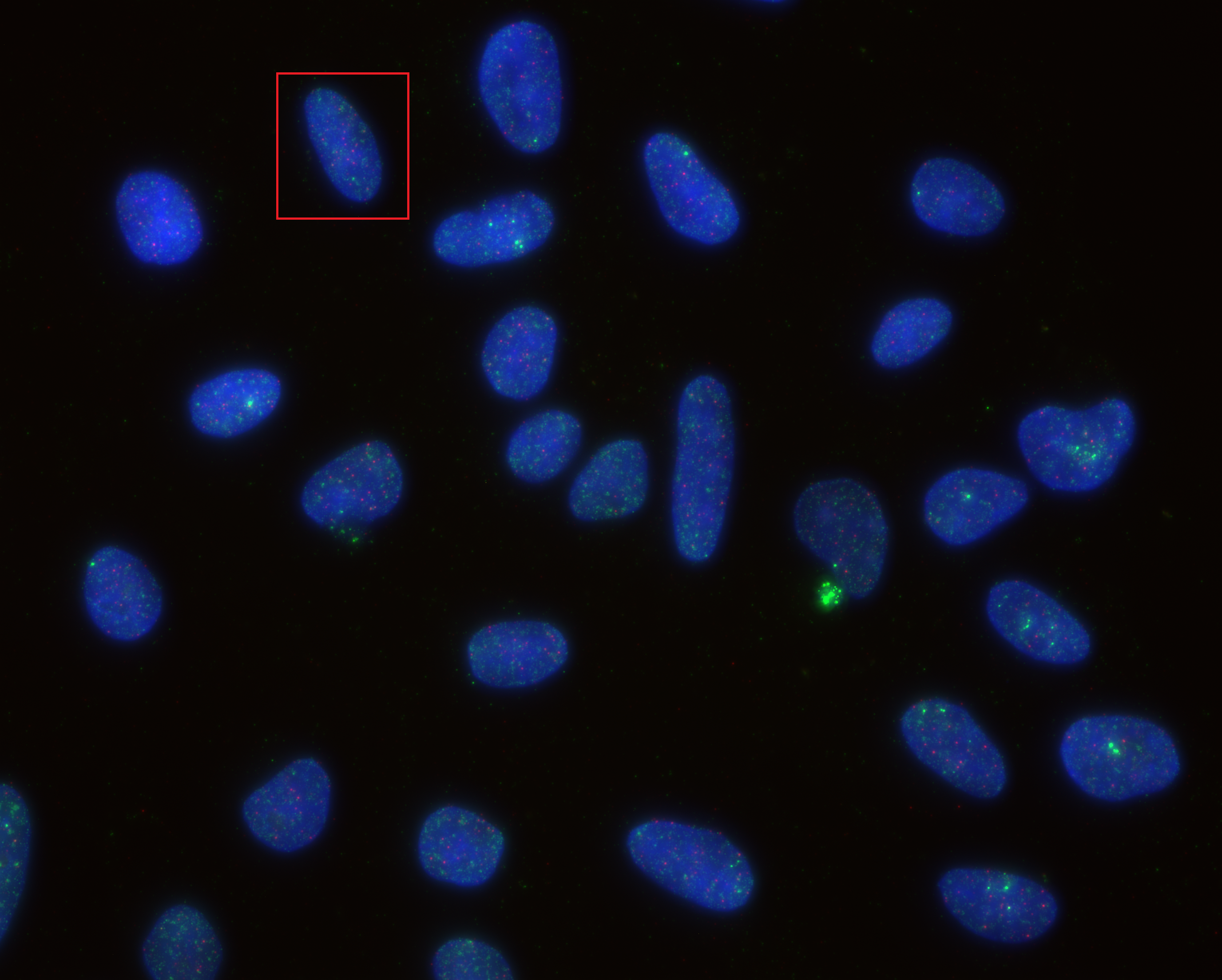

Supplement: Supplementary file 6 — Source data Fig. 5 [file 44319_2024_295_MOESM6_ESM.zip › Figure 5/5B/pS33+TRF2 image - representative nucleus - W89A siCt +dox.tif]

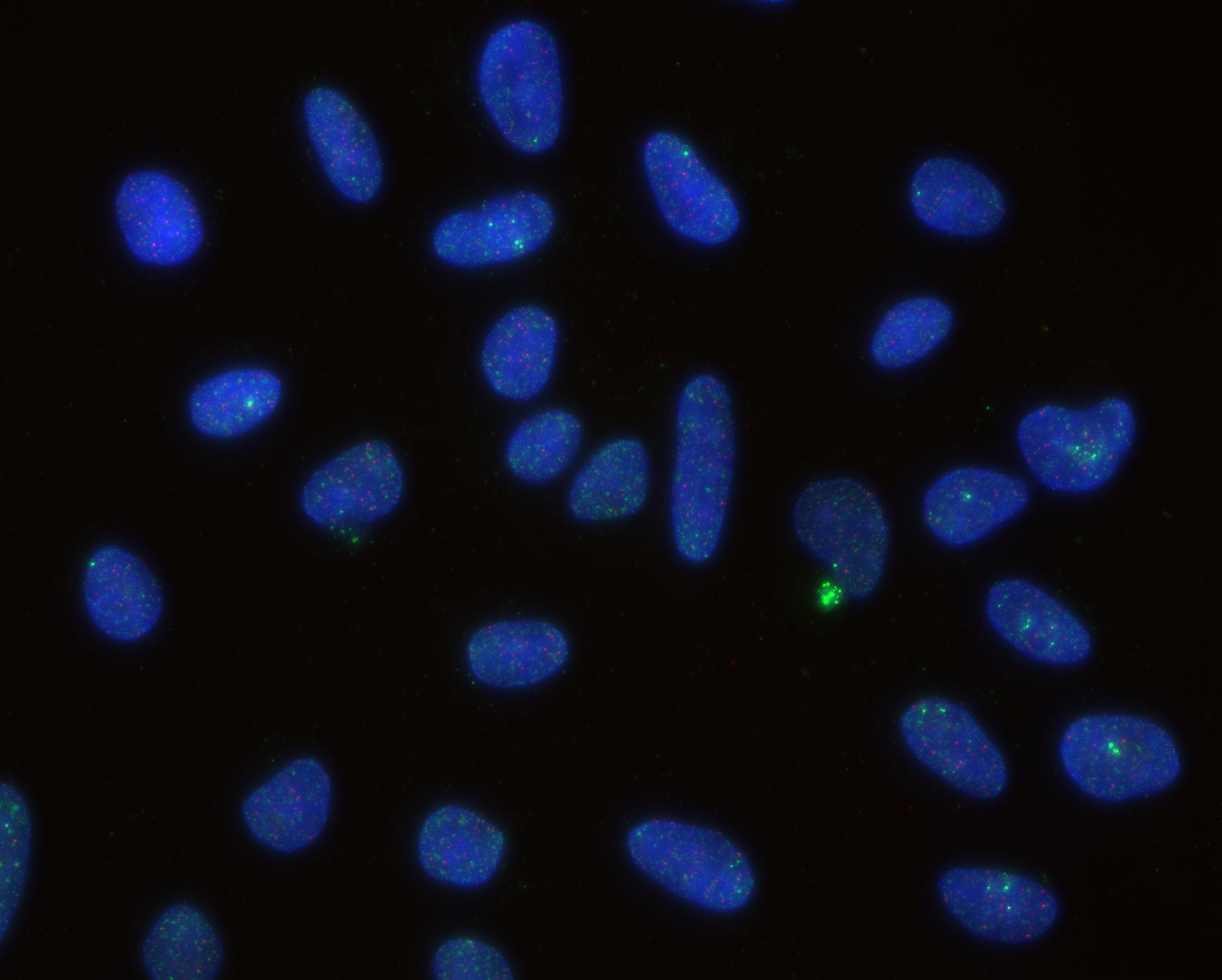

Supplement: Supplementary file 6 — Source data Fig. 5 [file 44319_2024_295_MOESM6_ESM.zip › Figure 5/5B/pS33+TRF2 image - W89A siCt +dox.tif]

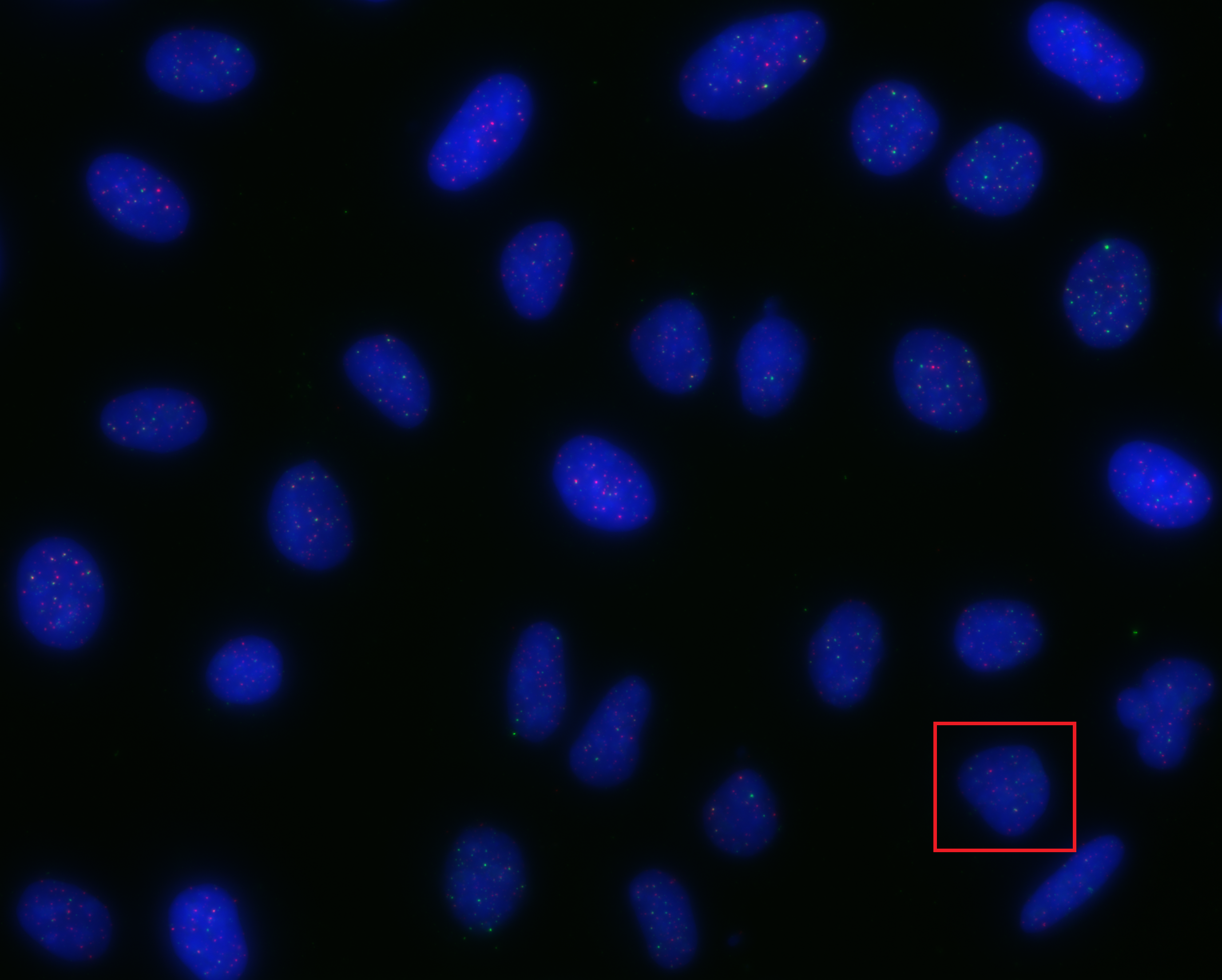

Supplement: Supplementary file 6 — Source data Fig. 5 [file 44319_2024_295_MOESM6_ESM.zip › Figure 5/5B/APBs image - representative nucleus - W89A siCt +dox.tif]

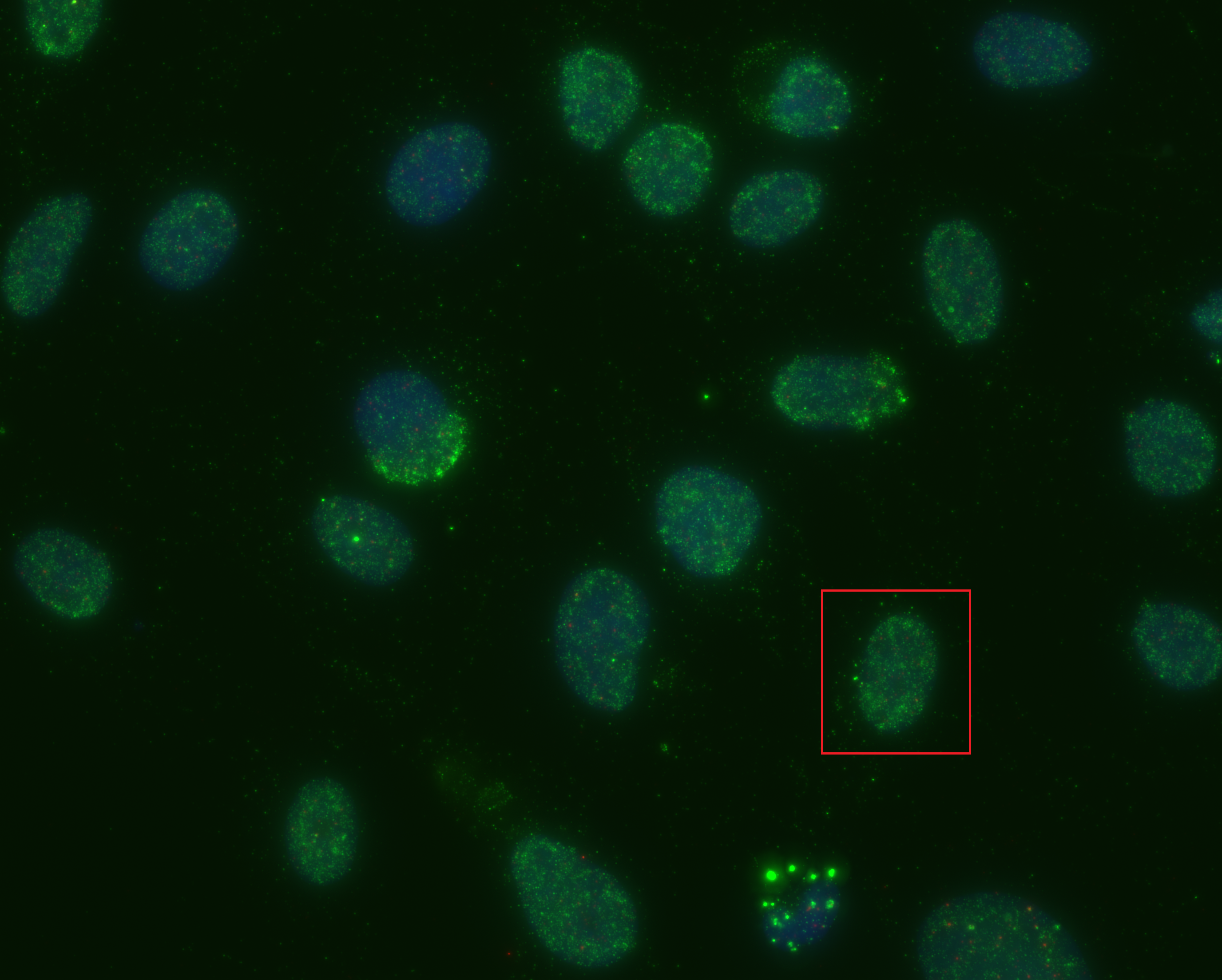

Supplement: Supplementary file 6 — Source data Fig. 5 [file 44319_2024_295_MOESM6_ESM.zip › Figure 5/5B/pS33+TRF2 image - representative nucleus - W89A siPc1 +dox.tif]

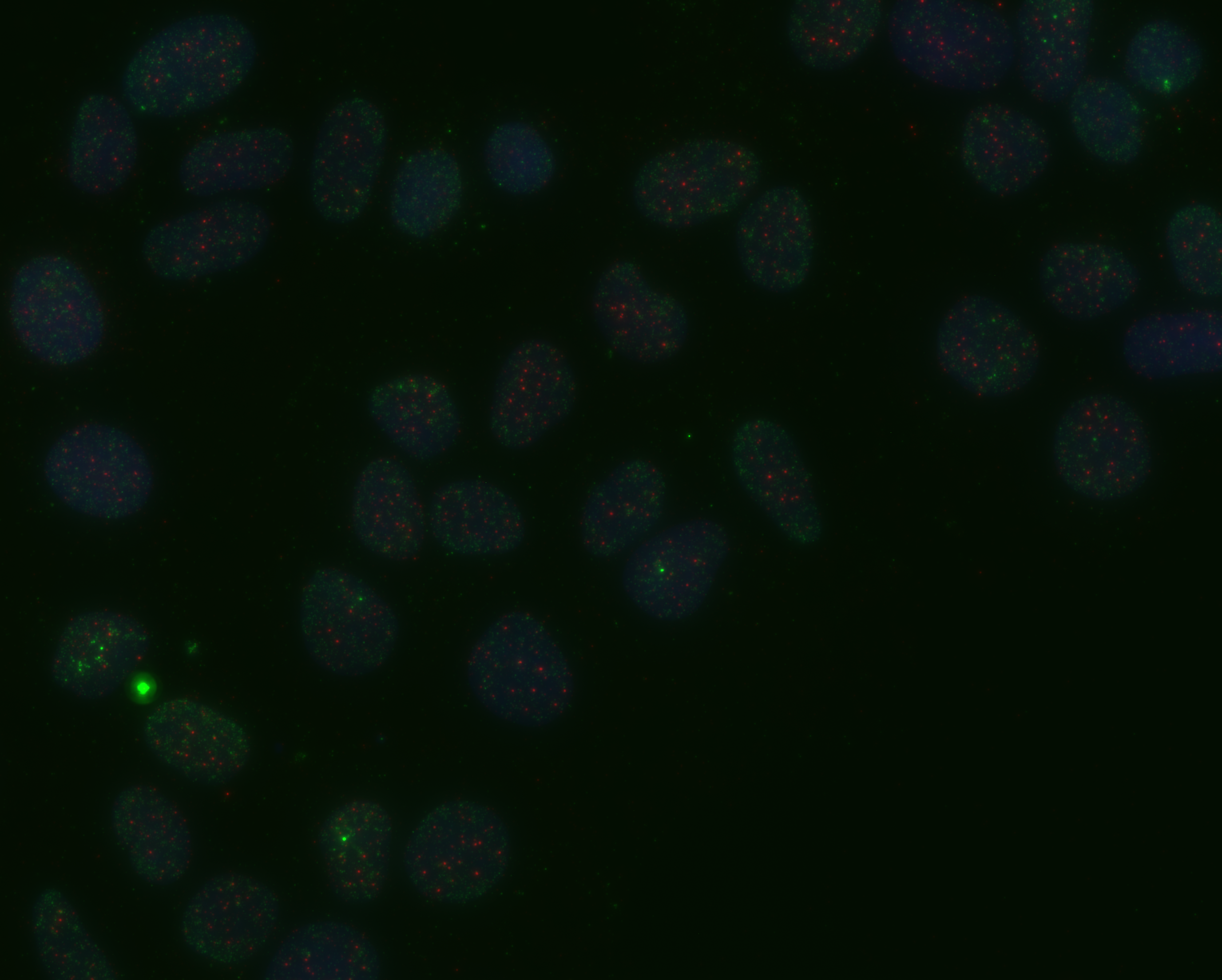

Supplement: Supplementary file 6 — Source data Fig. 5 [file 44319_2024_295_MOESM6_ESM.zip › Figure 5/5B/pS33+TRF2 image - PC4wt siCt +dox.tif]

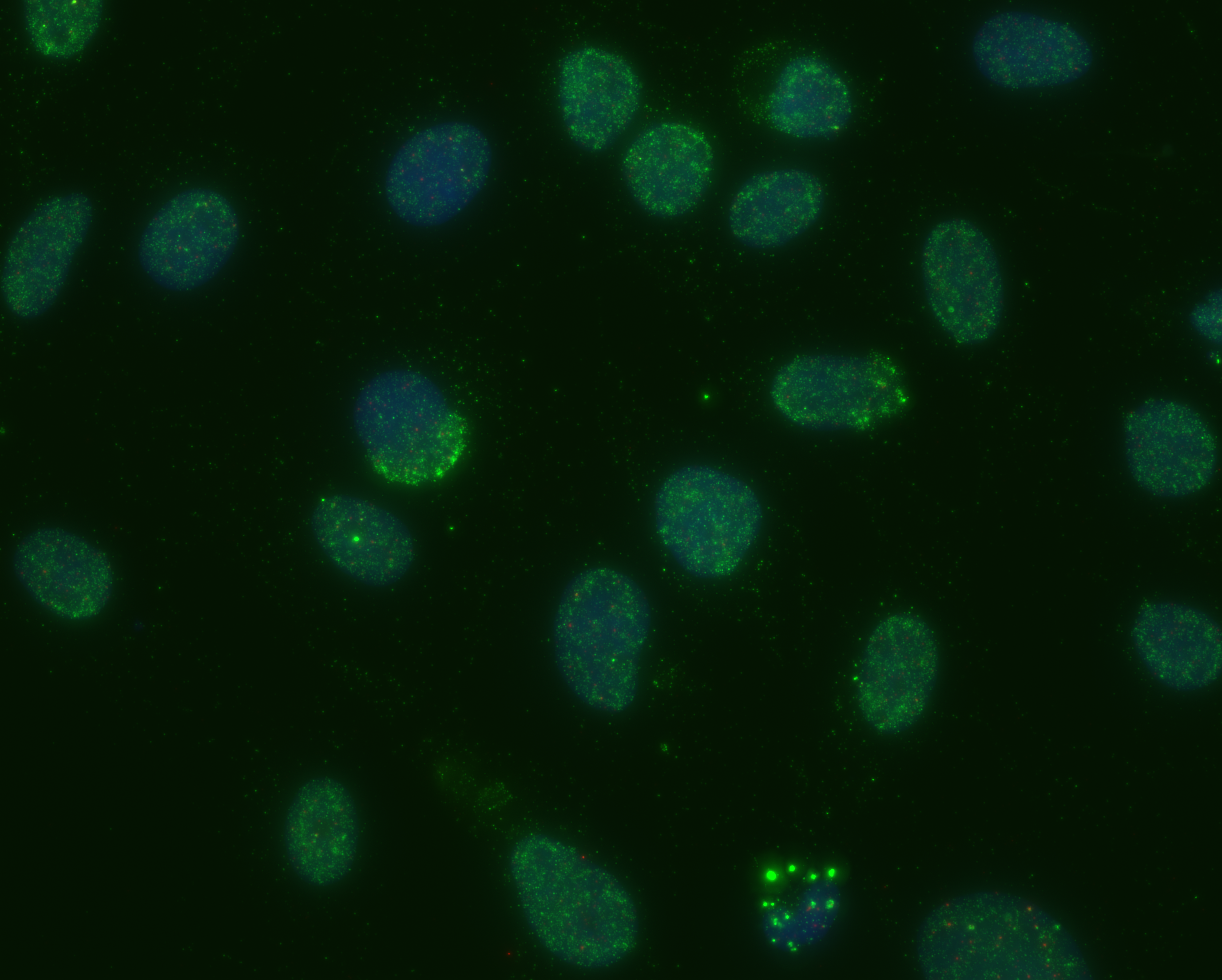

Supplement: Supplementary file 6 — Source data Fig. 5 [file 44319_2024_295_MOESM6_ESM.zip › Figure 5/5B/pS33+TRF2 image - W89A siPc1 +dox.tif]

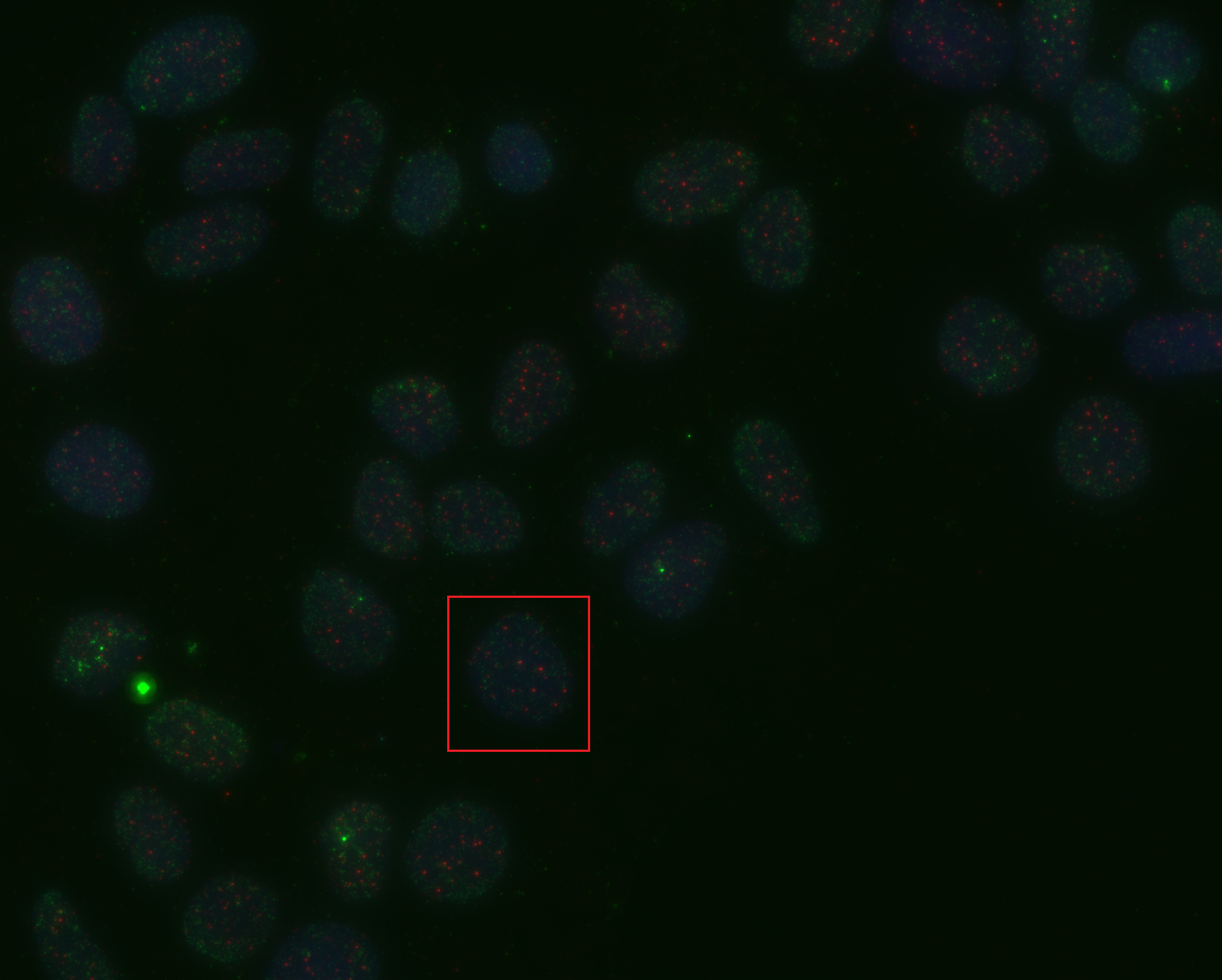

Supplement: Supplementary file 6 — Source data Fig. 5 [file 44319_2024_295_MOESM6_ESM.zip › Figure 5/5B/pS33+TRF2 image - representative nucleus - PC4wt siCt +dox.tif]

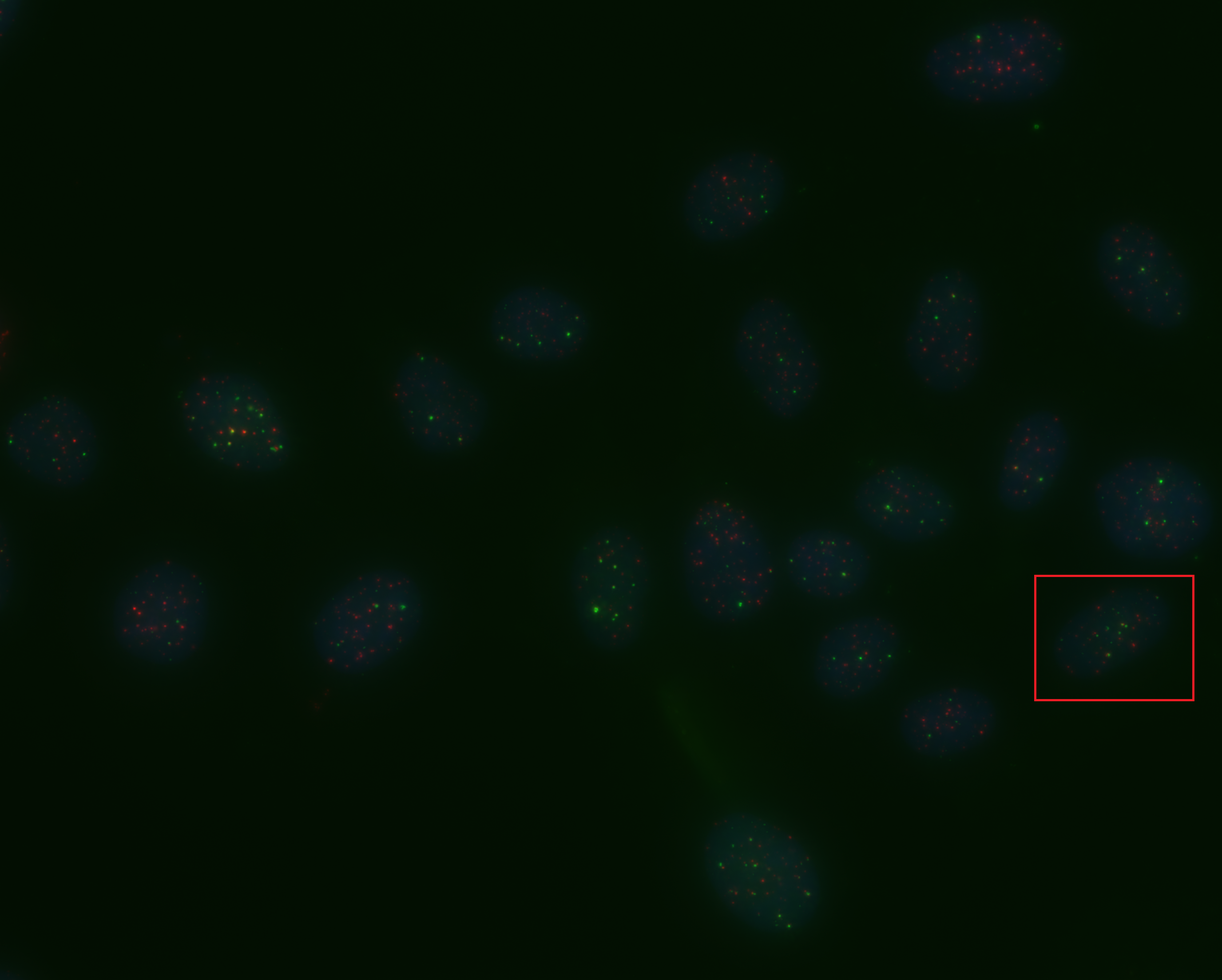

Supplement: Supplementary file 6 — Source data Fig. 5 [file 44319_2024_295_MOESM6_ESM.zip › Figure 5/5B/APBs image - representative nucleus - PC4wt siCt +dox.tif]

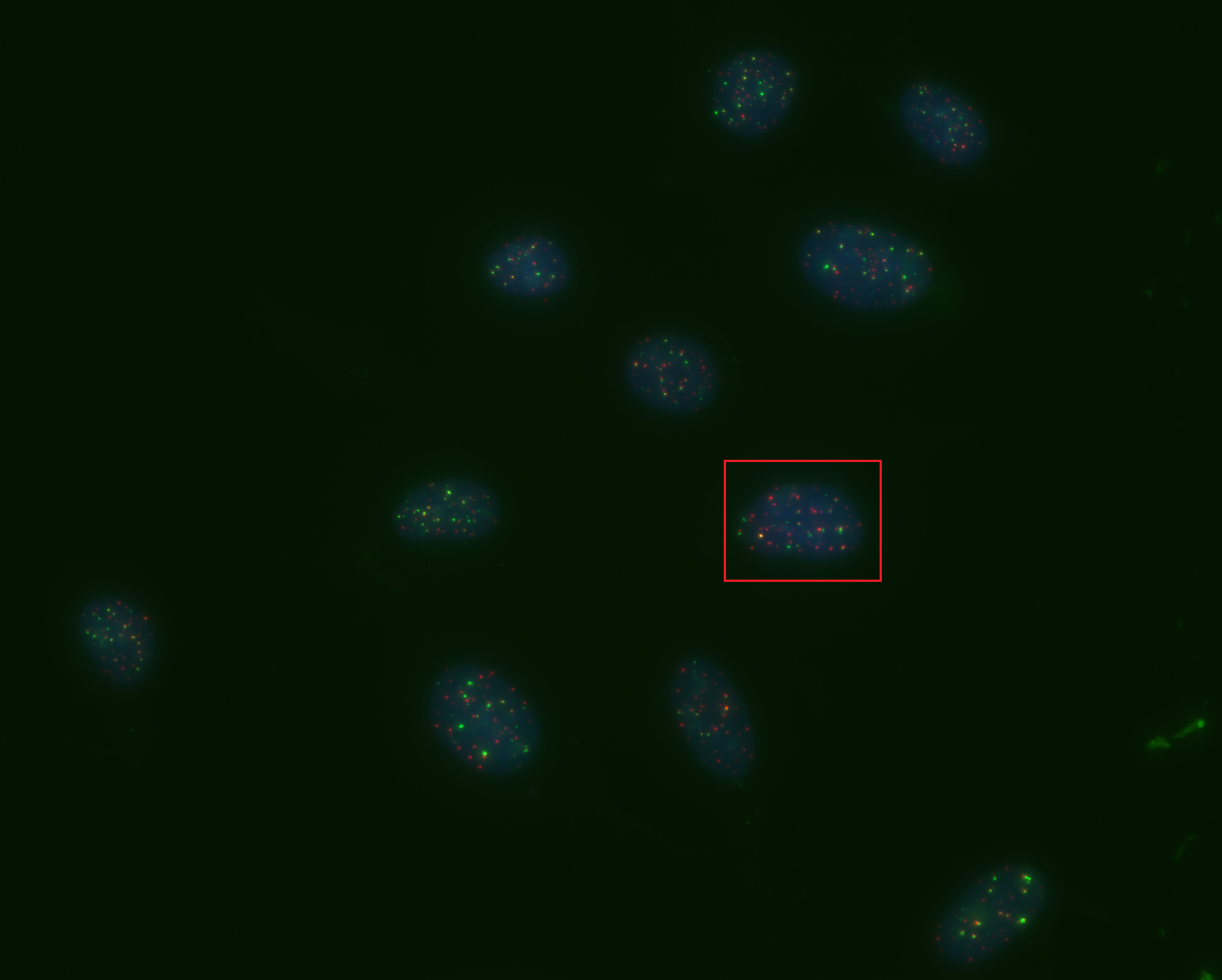

Supplement: Supplementary file 6 — Source data Fig. 5 [file 44319_2024_295_MOESM6_ESM.zip › Figure 5/5B/APBs image - representative nucleus - W89A siPc1 +dox.tif]

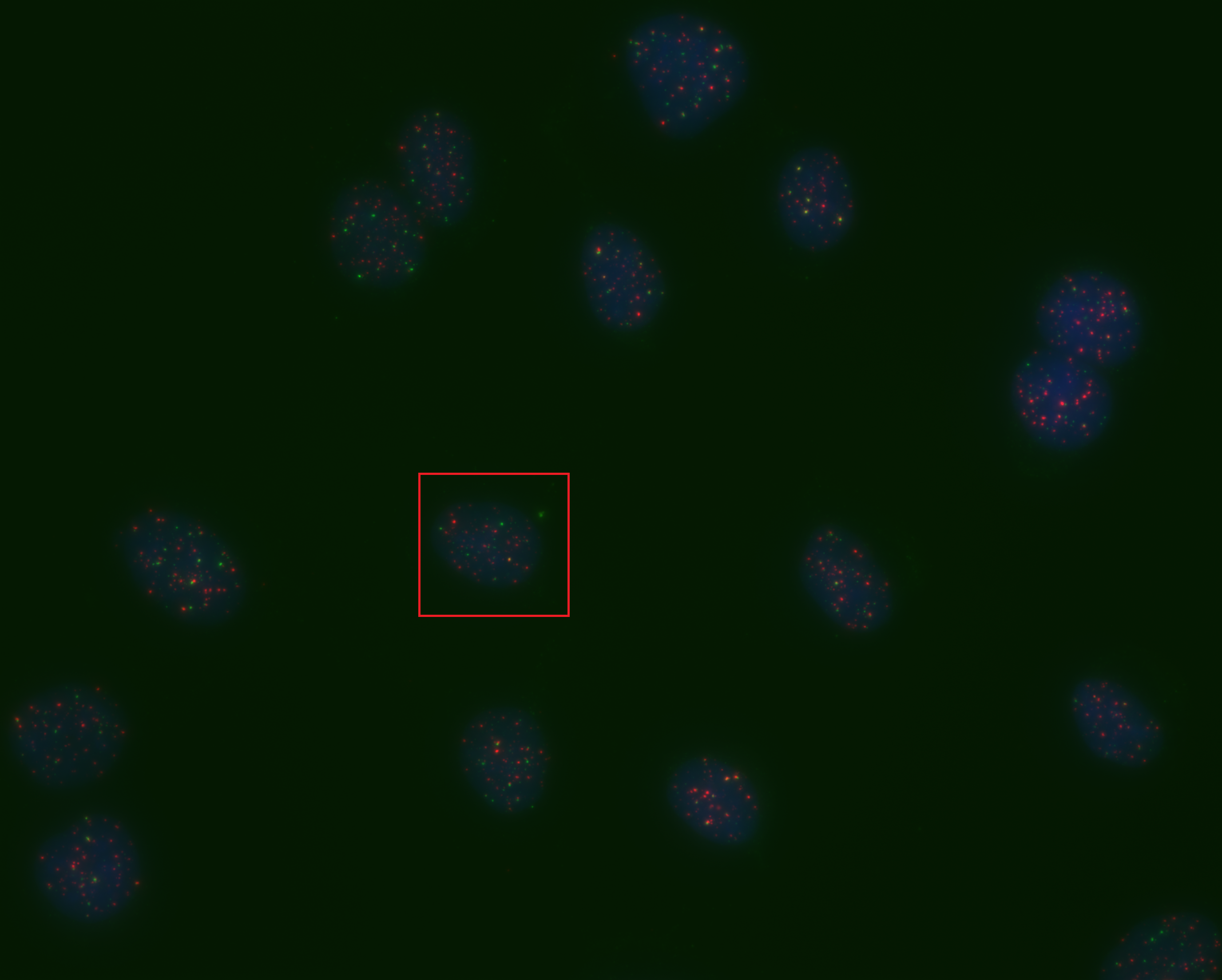

Supplement: Supplementary file 6 — Source data Fig. 5 [file 44319_2024_295_MOESM6_ESM.zip › Figure 5/5B/APBs image - representative nucleus - PC4wt siPc1 +dox.tif]

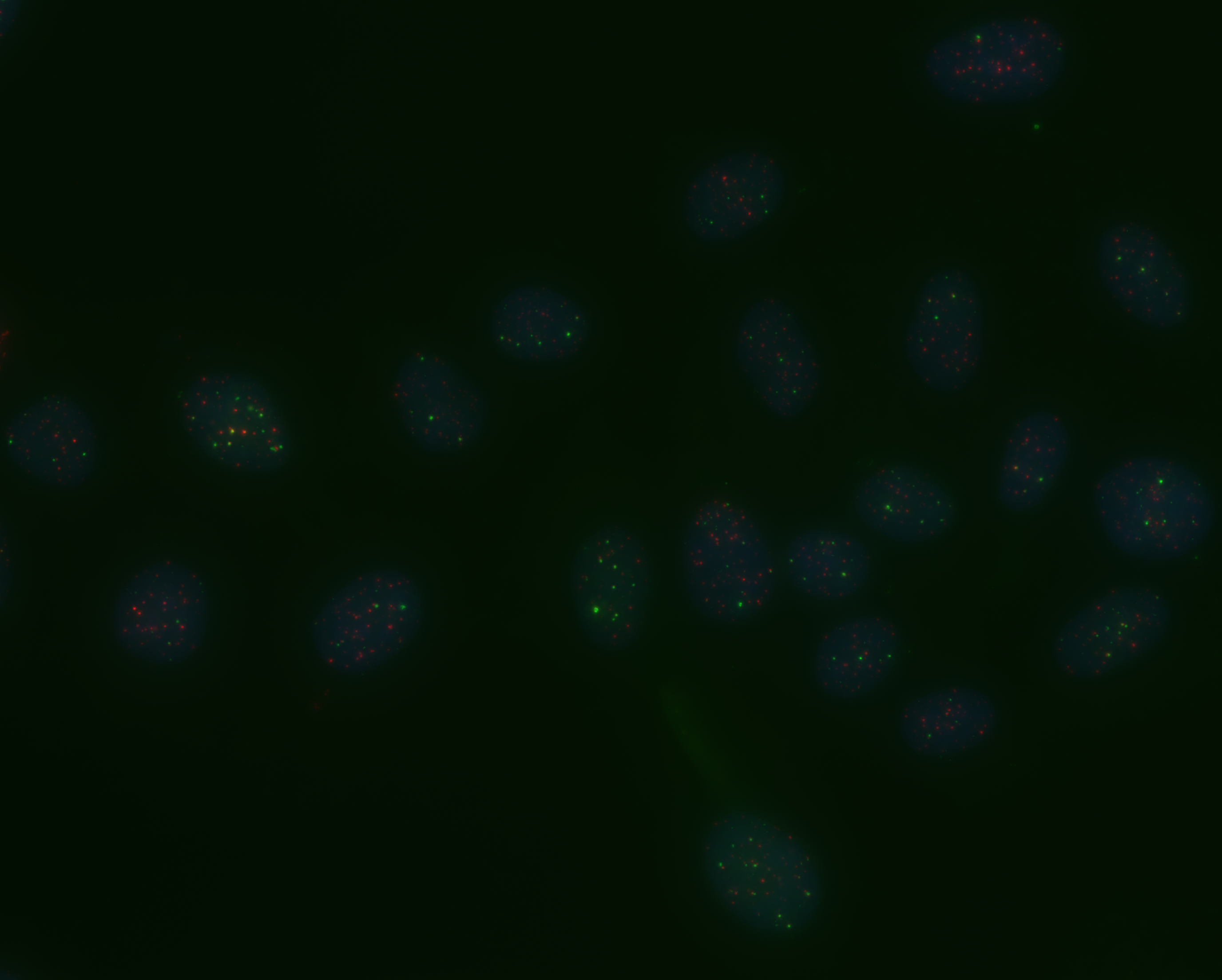

Supplement: Supplementary file 6 — Source data Fig. 5 [file 44319_2024_295_MOESM6_ESM.zip › Figure 5/5B/APBs image - PC4wt siCt +dox.tif]

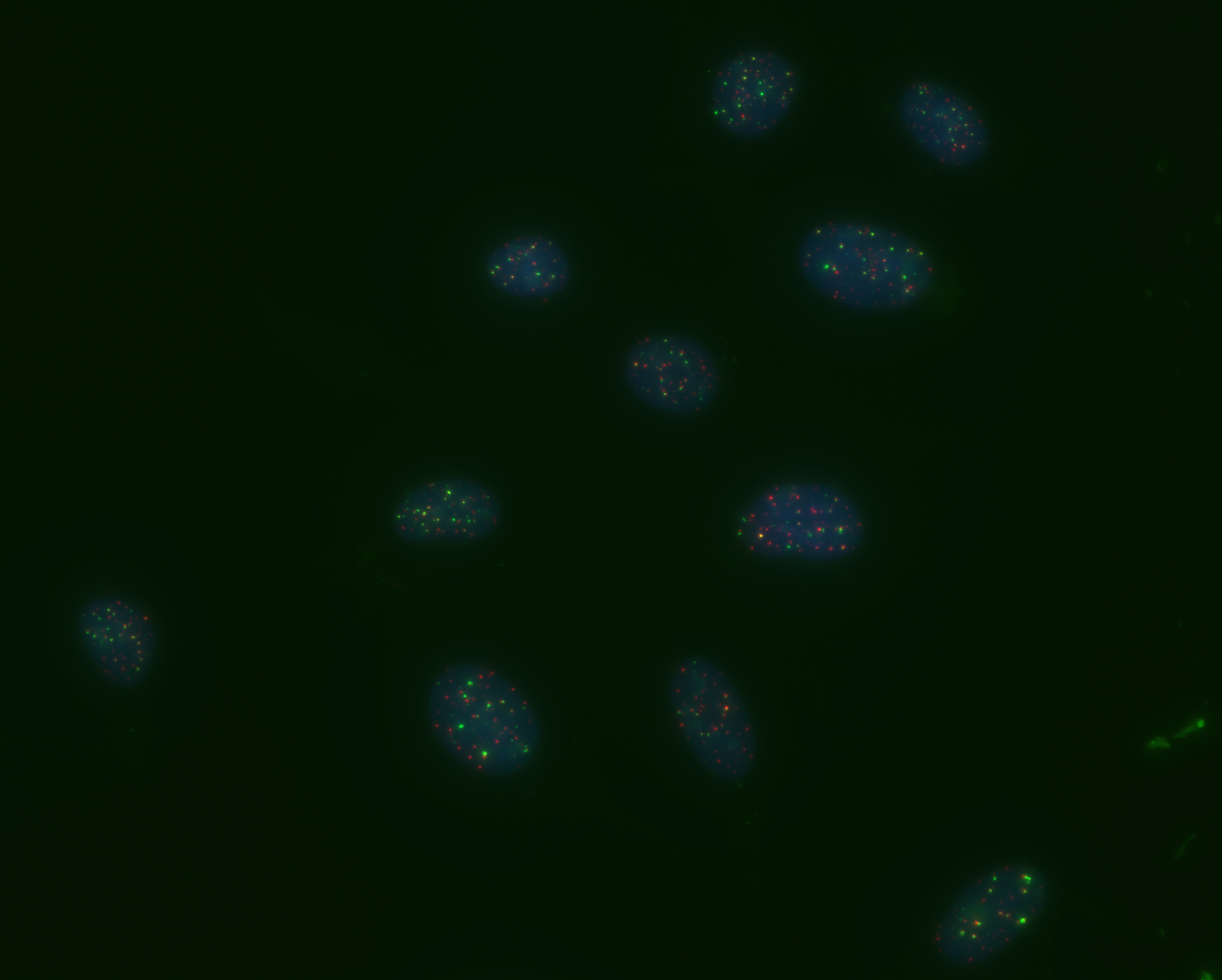

Supplement: Supplementary file 6 — Source data Fig. 5 [file 44319_2024_295_MOESM6_ESM.zip › Figure 5/5B/APBs image - W89A siPc1 +dox.tif]

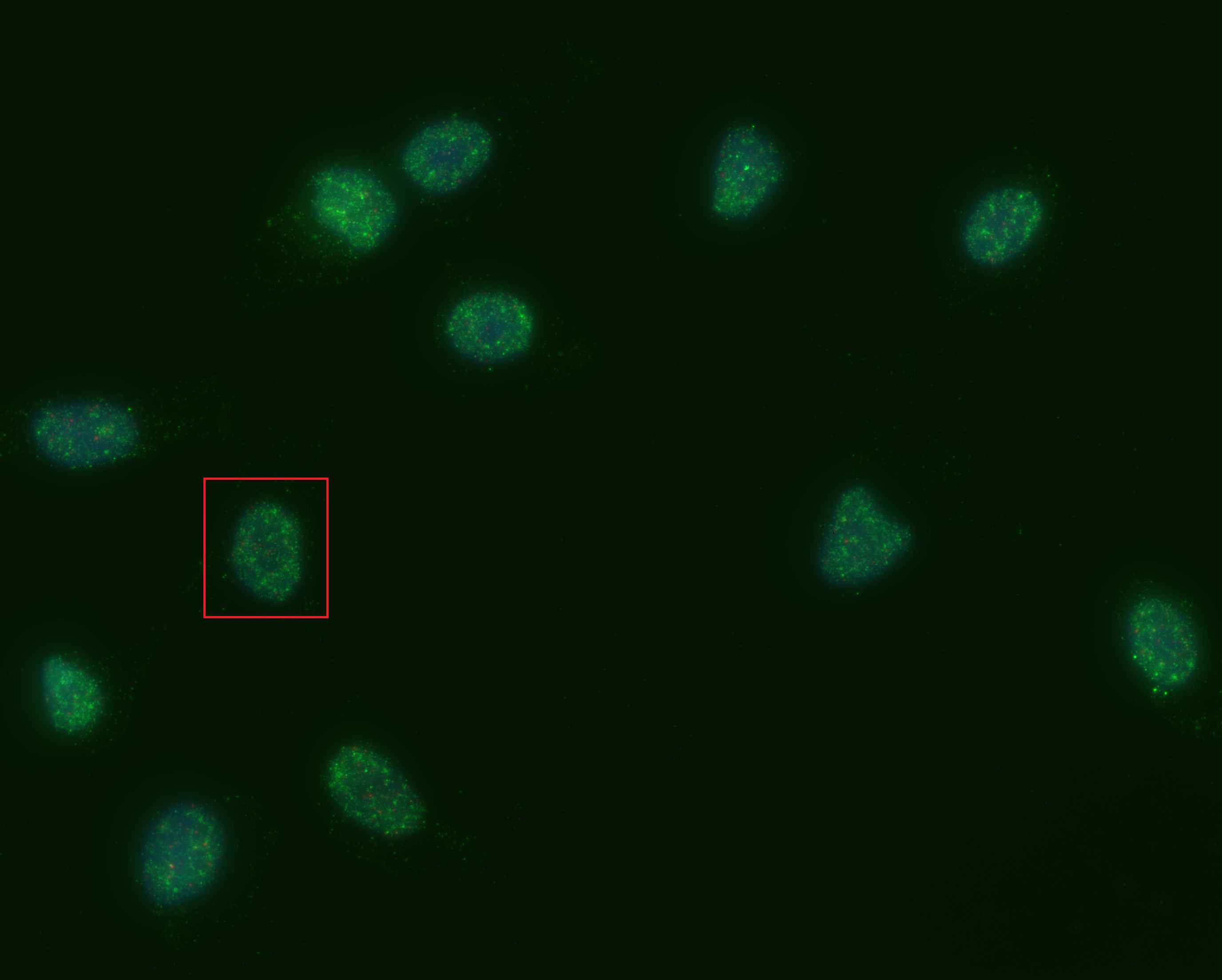

Supplement: Supplementary file 6 — Source data Fig. 5 [file 44319_2024_295_MOESM6_ESM.zip › Figure 5/5B/pS33+TRF2 image - representative nucleus - PC4wt siPc1 +dox.tif]

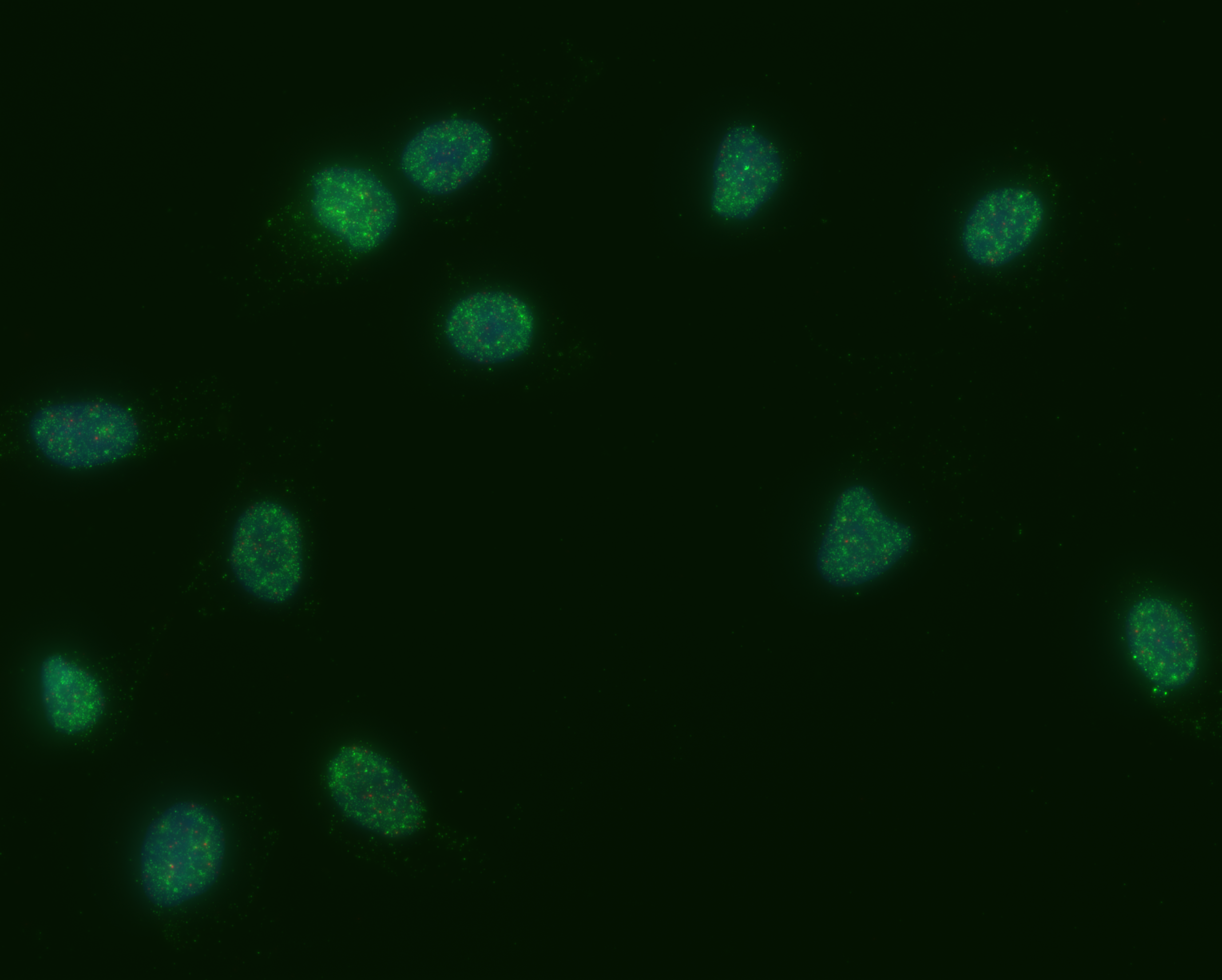

Supplement: Supplementary file 6 — Source data Fig. 5 [file 44319_2024_295_MOESM6_ESM.zip › Figure 5/5B/pS33+TRF2 image - PC4wt siPc1 +dox.tif]

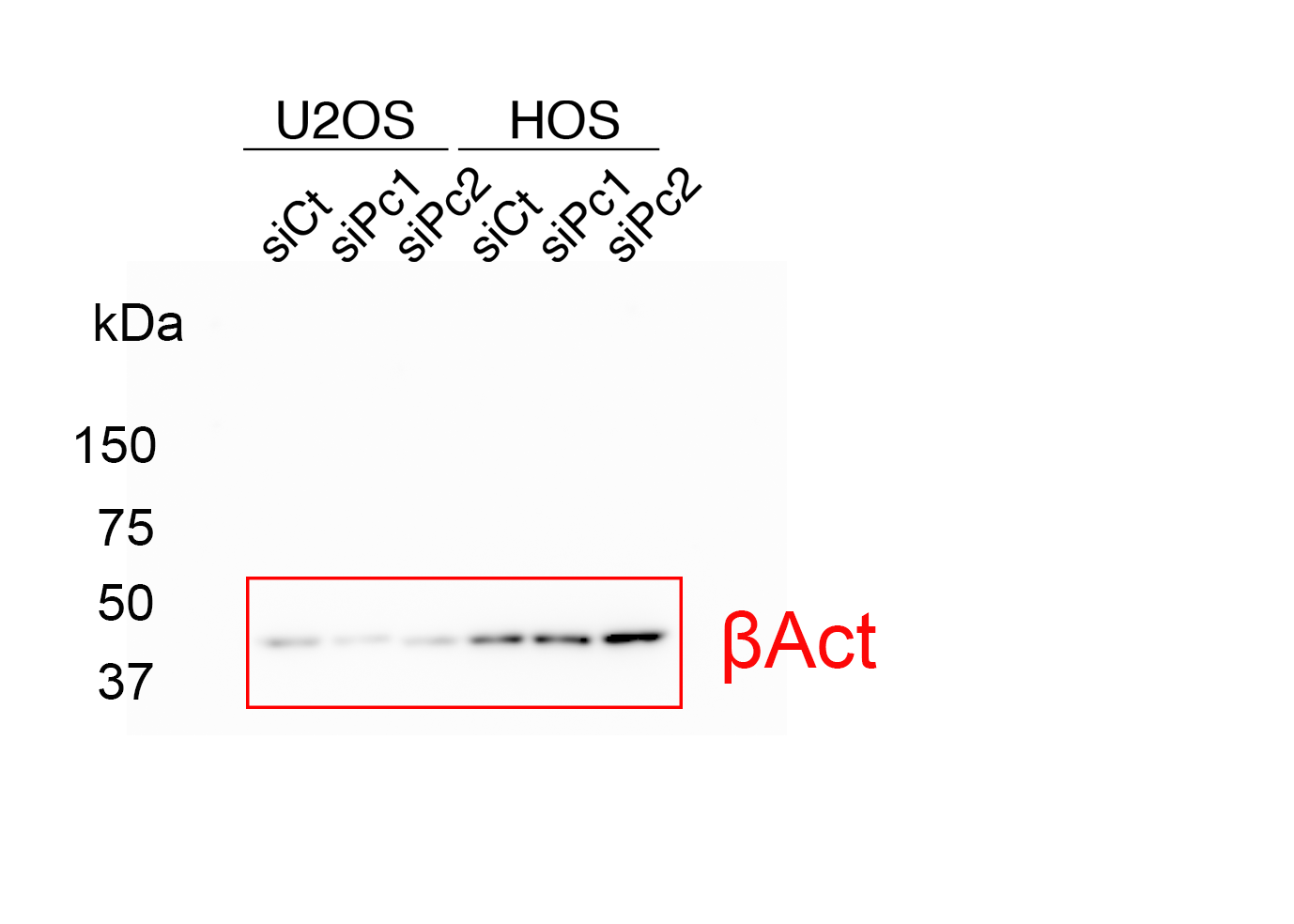

Supplement: Supplementary file 7 — Figure EV4A Source Data [file 44319_2024_295_MOESM7_ESM.zip › Figure EV4/A/WesternBlot-Actin.tif]

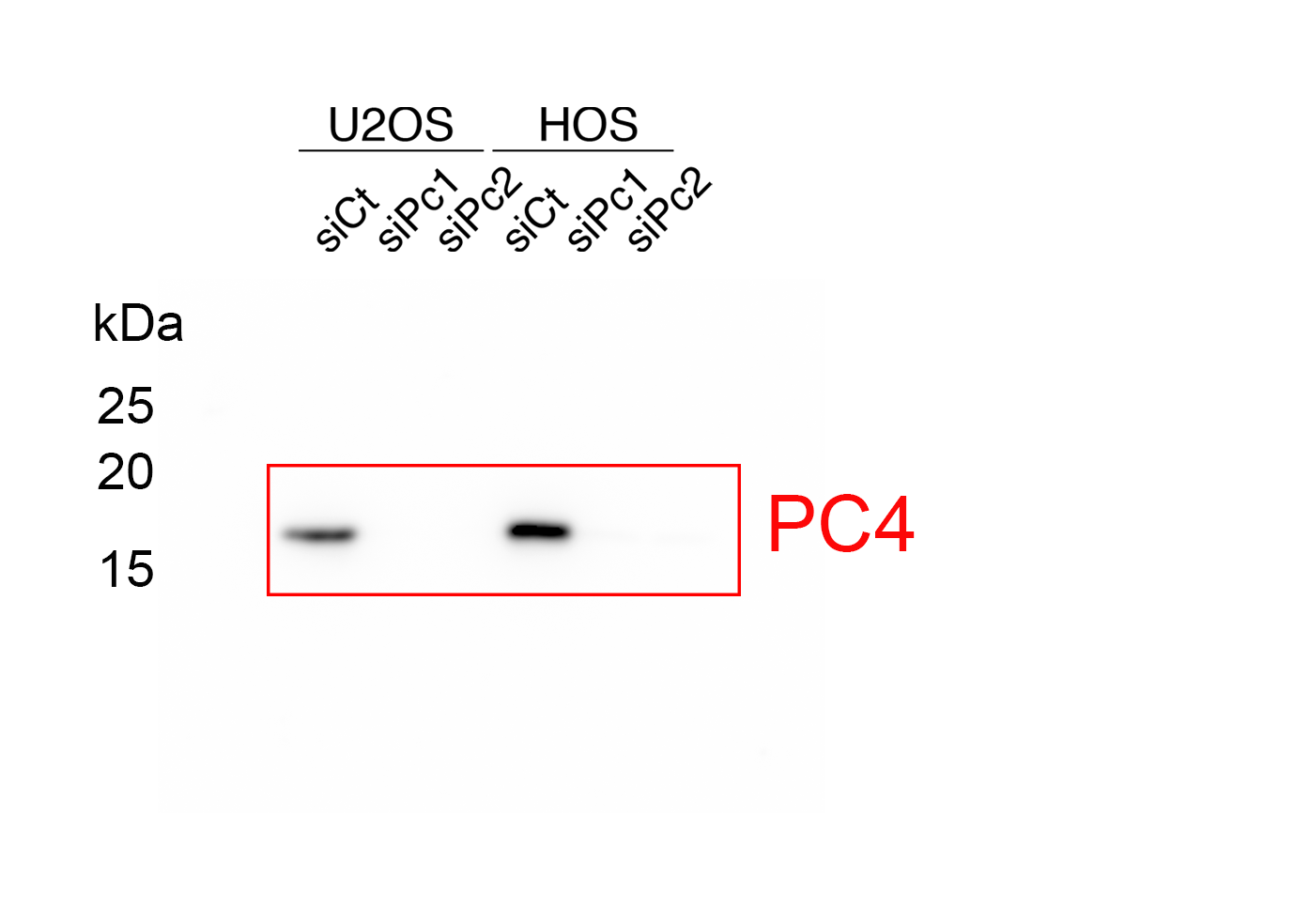

Supplement: Supplementary file 7 — Figure EV4A Source Data [file 44319_2024_295_MOESM7_ESM.zip › Figure EV4/A/WesternBlot-PC4.tif]

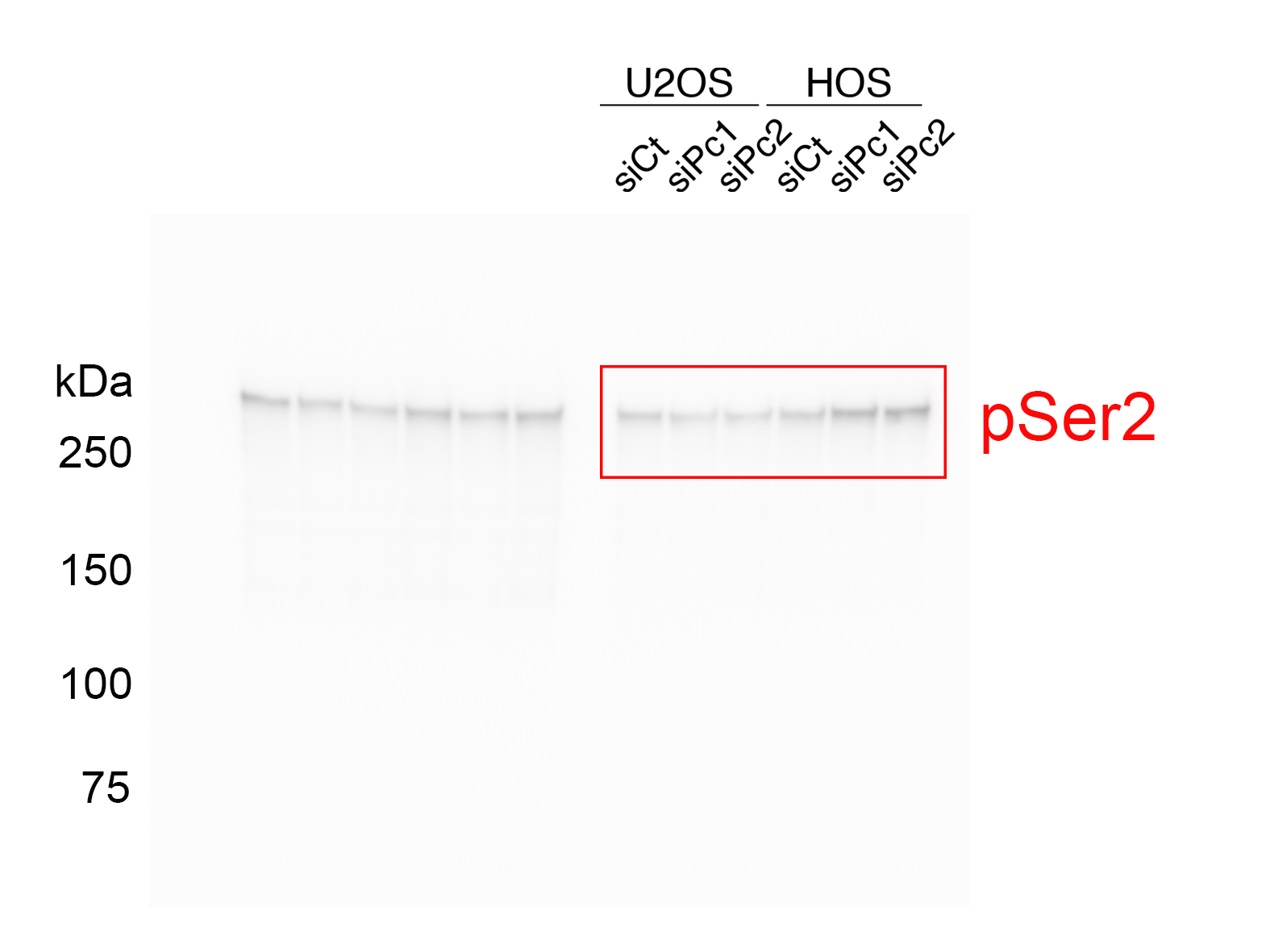

Supplement: Supplementary file 7 — Figure EV4A Source Data [file 44319_2024_295_MOESM7_ESM.zip › Figure EV4/A/WesternBlot-pSer2.tif]

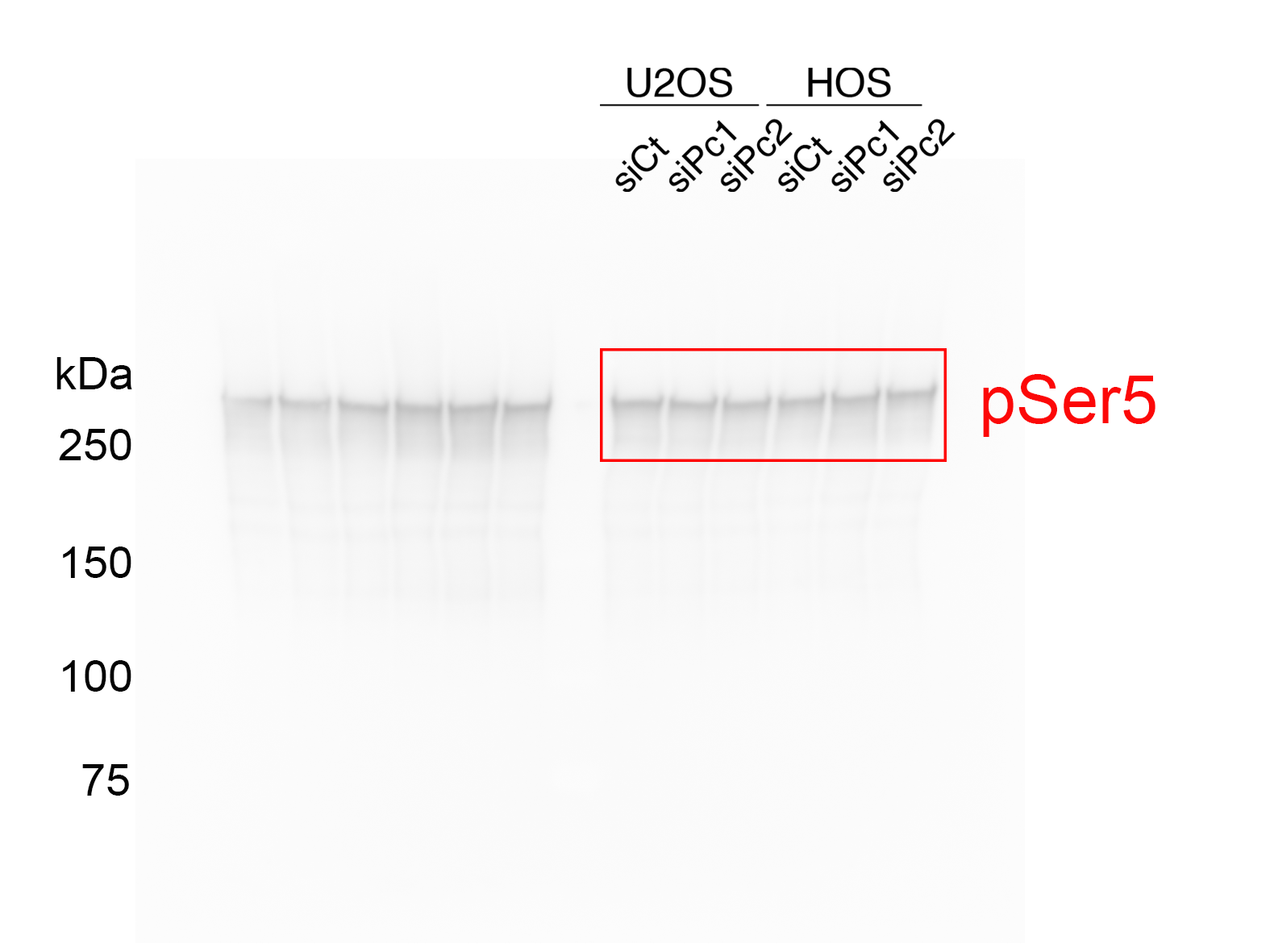

Supplement: Supplementary file 7 — Figure EV4A Source Data [file 44319_2024_295_MOESM7_ESM.zip › Figure EV4/A/WesternBlot-pSer5.tif]

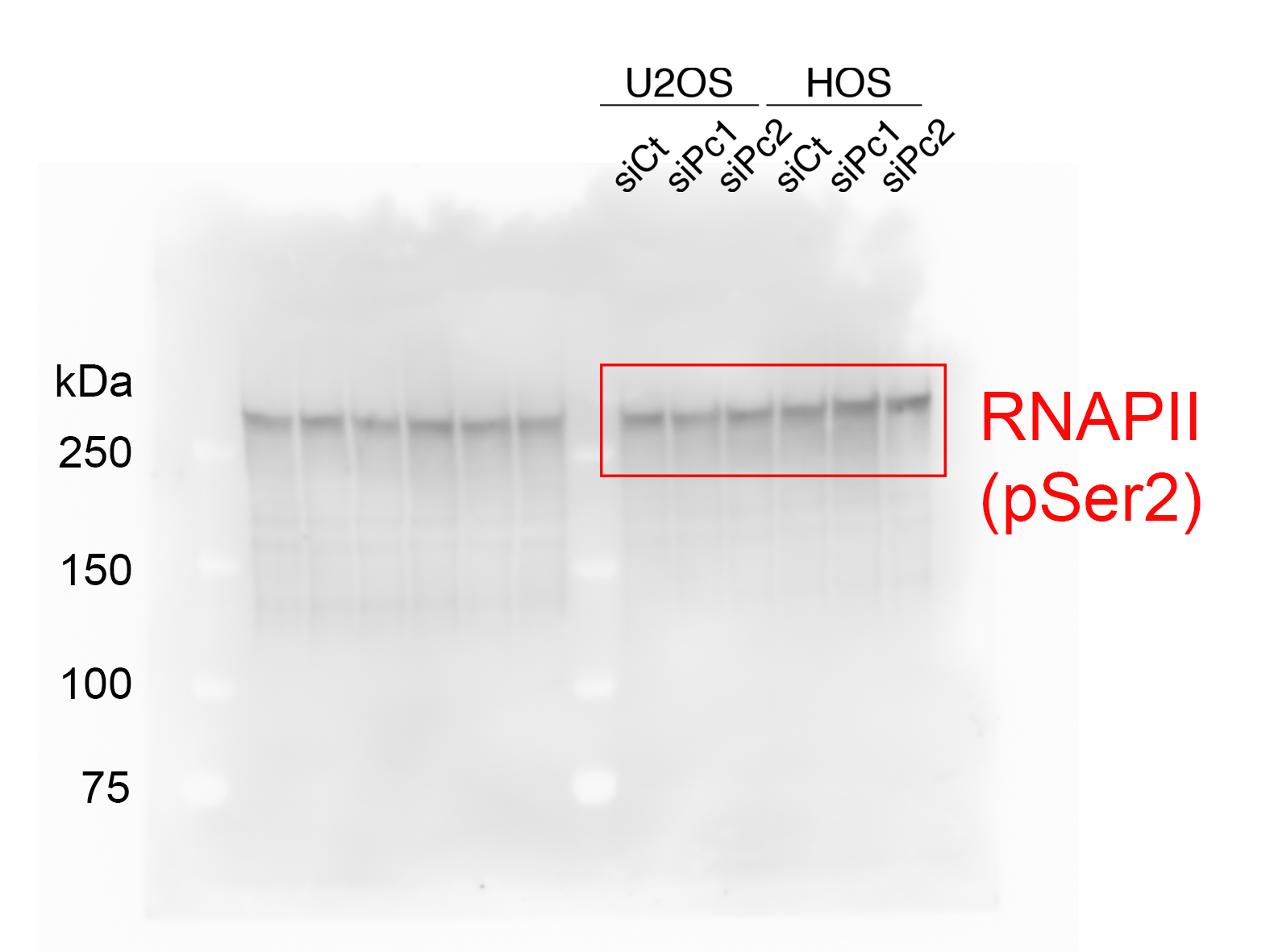

Supplement: Supplementary file 7 — Figure EV4A Source Data [file 44319_2024_295_MOESM7_ESM.zip › Figure EV4/A/WesternBlot-RNAPII(pSer2).tif]

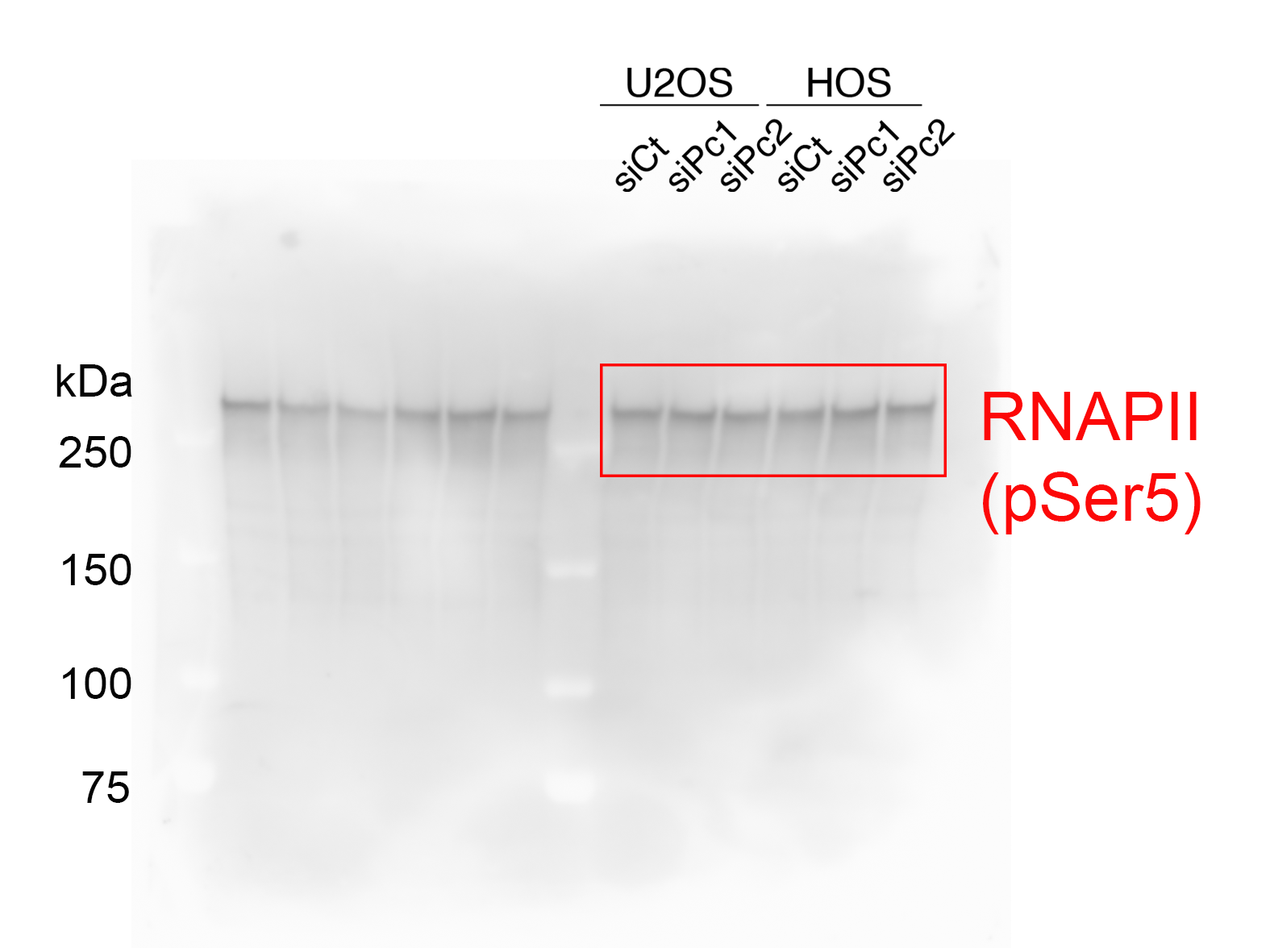

Supplement: Supplementary file 7 — Figure EV4A Source Data [file 44319_2024_295_MOESM7_ESM.zip › Figure EV4/A/WesternBlot-RNAPII(pSer5).tif]
