# Supplementary material for: Human PC4 supports telomere stability and viability in cells utilizing the alternative lengthening of telomeres mechanism
Source: EMBO Rep. 2024 Oct 28;25(12):5294–315. doi: 10.1038/s44319-024-00295-3 (PMC11624207; doi:10.1038/s44319-024-00295-3)
Supplement: Supplementary file 8 — Expanded View Figures [file 44319_2024_295_MOESM8_ESM.pdf]

Expanded View Figures

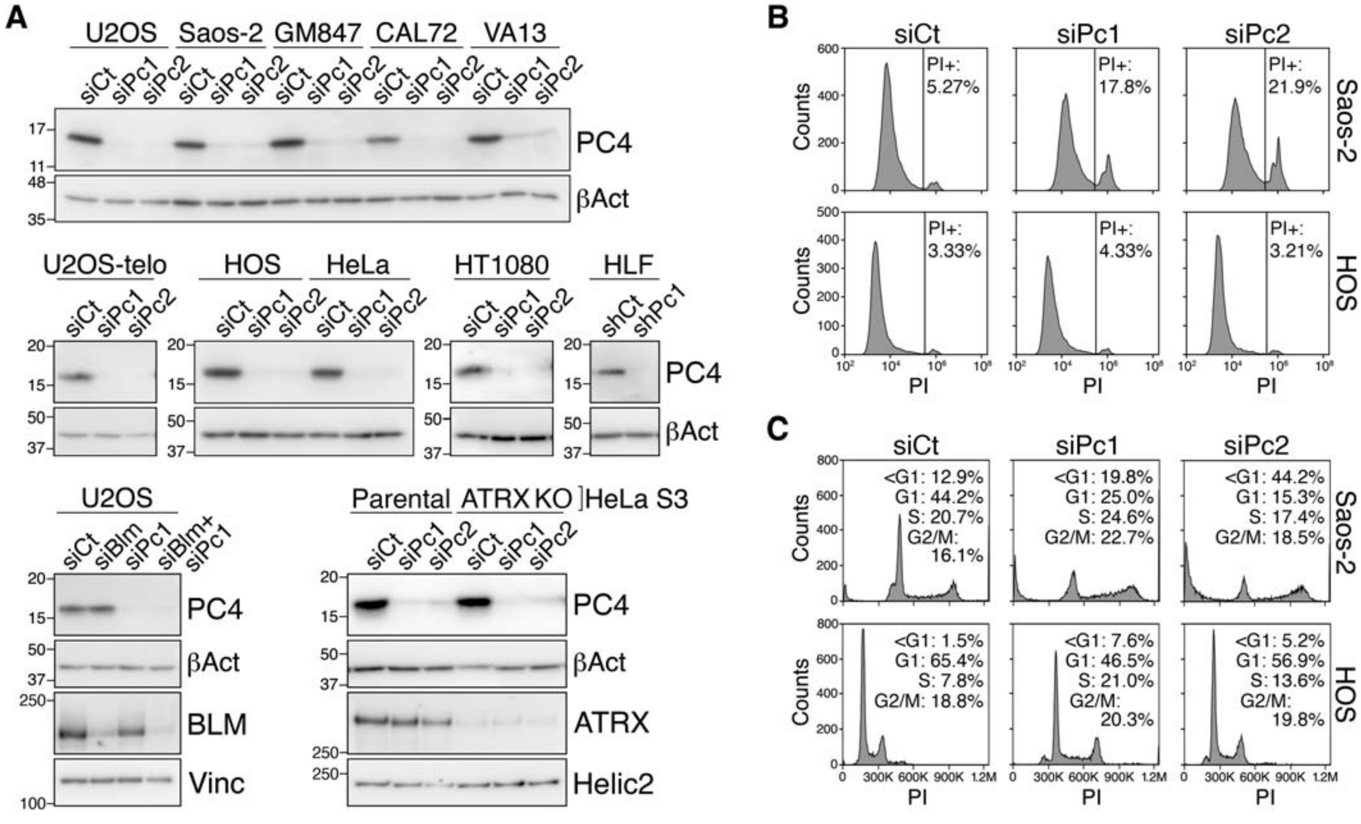

Figure EV1. PC4 depletion induces death in ALT cells.

(**A**) Western blot analysis of PC4, BLM, and ATRX protein levels. Cells were transfected with siRNAs or infected with shRNA lentiviruses, and total proteins were extracted 72 h after siRNA transfections or 5 days after shRNA infections. Beta Actin (βAct), Vinculin (Vinc), and Helic2 serve as loading controls. Marker molecular weights are on the left of the gels in kDa. (**B**) Examples of FACS profiles of cells stained with PI without permeabilization. The indicated cell lines were depleted of PC4 for 9 days. Cell counts (y-axis) are plotted against PI intensity (x-axis) for one representative experiment. Numbers are percentages of cells positive to PI staining as defined by the indicated gates (vertical bars). (**C**) Examples of FACS profiles of Saos-2 and HOS cells ethanol-fixed and stained with PI. Cells were transfected with siRNAs every 72 h for a total of 9 and 15 days, respectively. Cell counts (y-axis) are plotted against PI intensity (x-axis). The percentages of cells with different DNA contents, including sub-G1 (<G1), are indicated.

 © The Author(s)

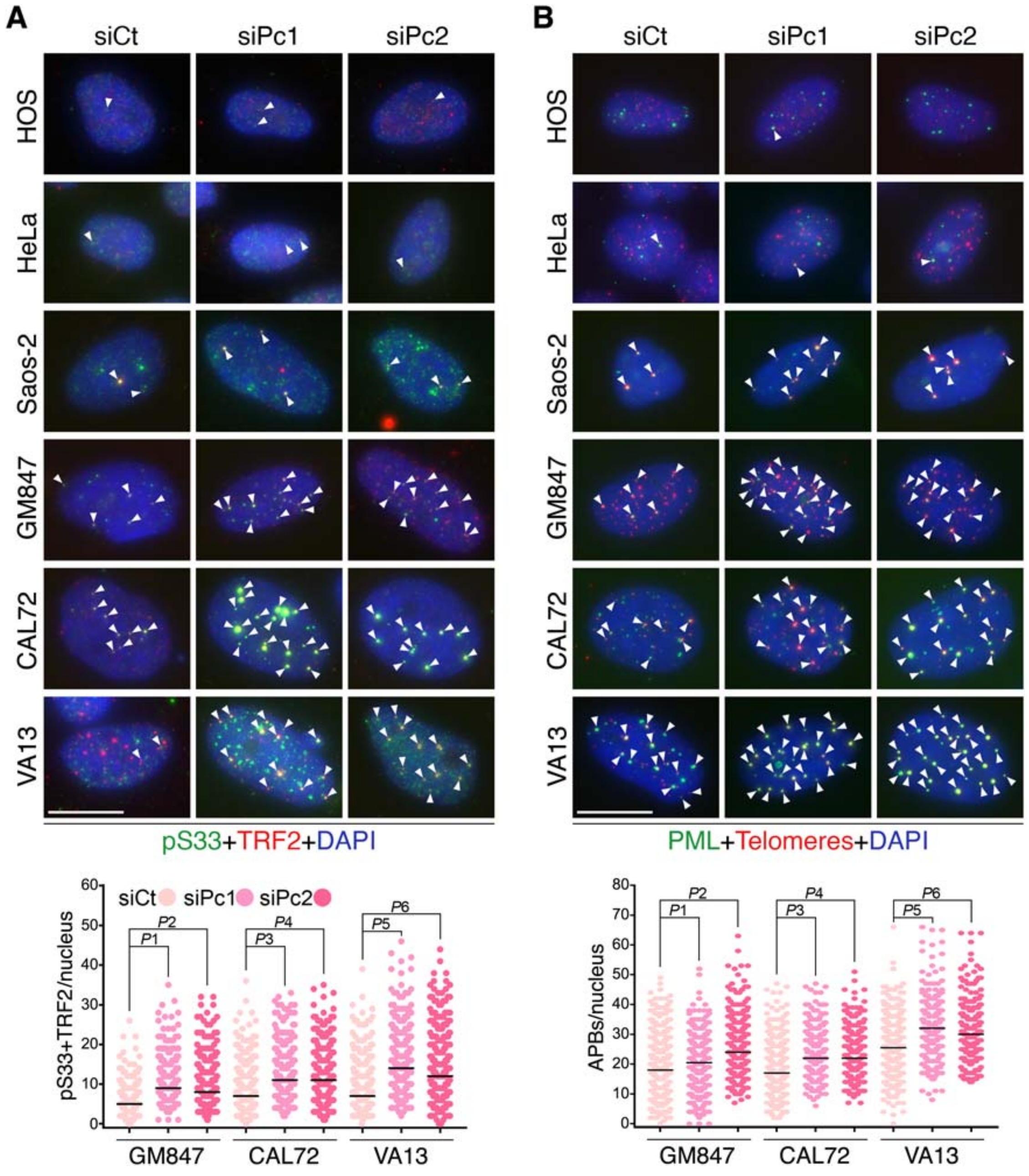

Figure EV2. PC4 restricts telomeric replication stress and APBs.

Examples of pS33 (green) and TRF2 (red) double IF (**A**) and of PML IF (green) combined with telomeric DNA FISH (red; (**B**)) in the indicated cell lines transfected with siRNAs and harvested 72 h after transfection. DAPI-stained DNA is in blue. Arrowheads point to co-localization events. The plots at the bottom show the co-localization events per nucleus. At least 100 nuclei were analyzed for each sample in each of the three biological replicates. For the plots in (**A**): $P1 < 0.0001$; $P2 < 0.0001$; $P3 < 0.0001$; $P4 < 0.0001$; $P5 < 0.0001$; $P6 < 0.0001$. For the plots in (**B**): $P1 = 0.001$; $P2 < 0.0001$; $P3 < 0.0001$; $P4 < 0.0001$; $P5 < 0.0001$; $P6 < 0.0001$ (Mann–Whitney *U*-test). Scale bars: 10 µm.

© The Author(s)

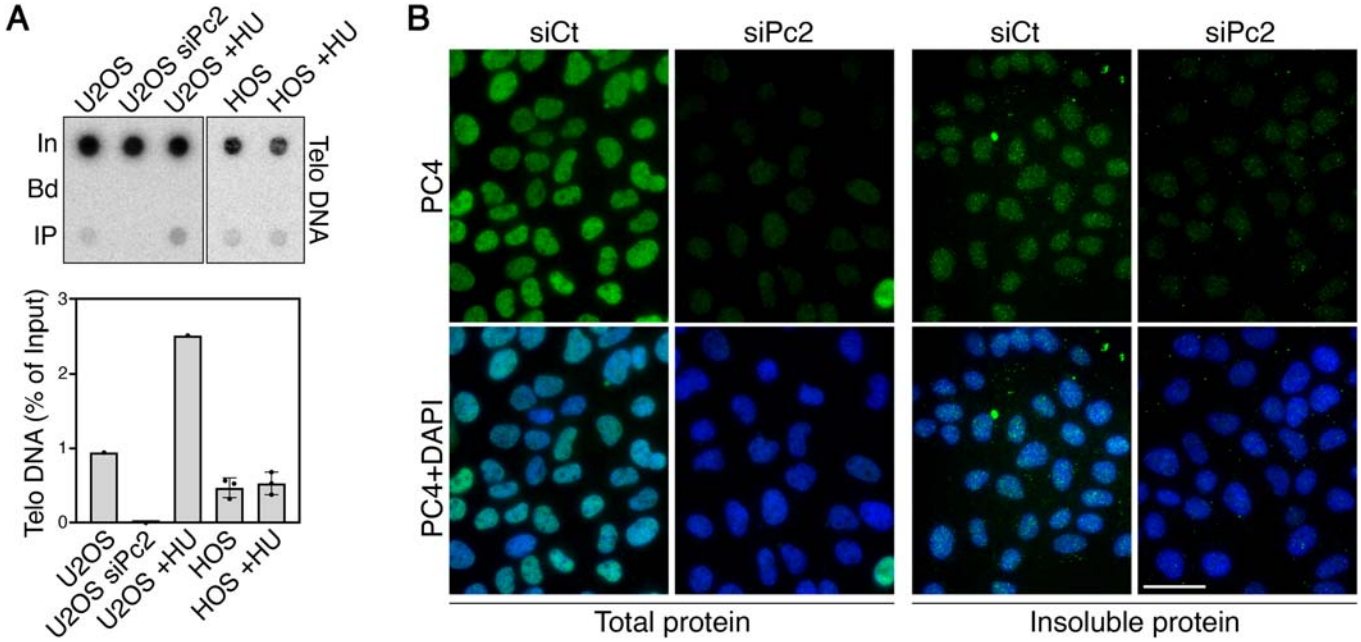

Figure EV3. PC4 is a nuclear protein, partly bound to chromatin regions including telomeres.

(**A**) Dot-blot hybridization of endogenous PC4 ChIPs in U2OS or HOS cells using radiolabeled probes to detect telomeric (Telo) DNA. Cells were either transfected with siPc2 and harvested 72 h after transfection, or treated with 0.2 mM hydroxyurea (HU) for 16 h. In: Input (1%), Bd: only beads control (50%), IP: PC4 immunoprecipitation (50%). Signals were quantified and graphed (bottom) as the fraction of input DNA found in the corresponding IP samples, after subtraction of Bd-associated signals. For U2OS, results are from one biological replicate. For HOS, bars and error bars are means and SDs from three biological replicates. The disappearance of telomeric DNA signal in the IP fraction of PC4-depleted U2OS cells confirms the specificity of the antibody. (**B**) Examples of PC4 (green) immunostaining in U2OS cells transfected with siPc2 or siCt and harvested 72 h after transfection. Cells were either permeabilized with mild detergent prior to fixation (right panels) or left untreated (left panels), in order to visualize chromatin-bound PC4 and total PC4, respectively. The substantial decrease in staining in PC4-depleted cells confirms the specificity of the antibody. Scale bar: 30 µm.

 © The Author(s)

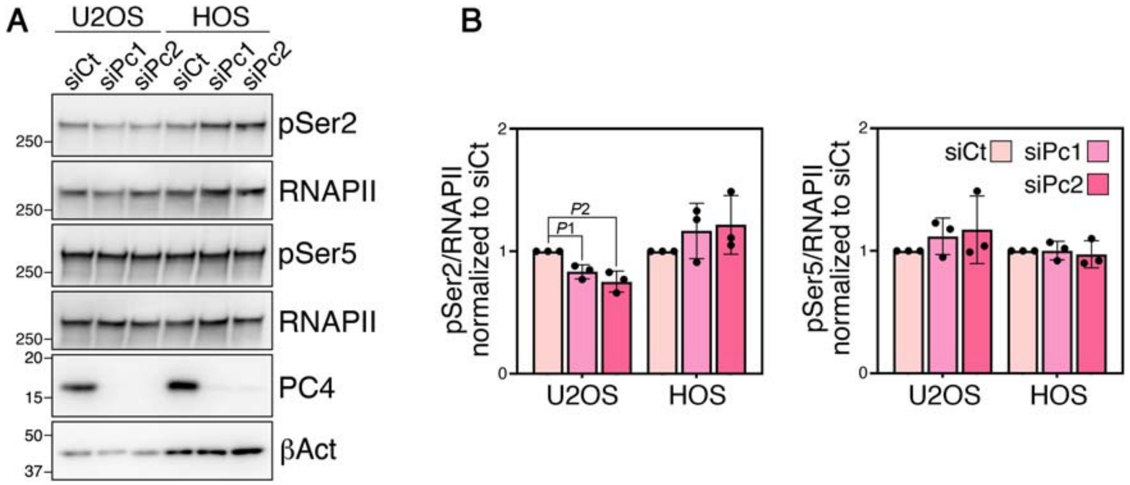

Figure EV4. Effects of PC4 depletion on RNAPII phosphorylation.

(**A**) Western blot analysis of PC4, RNAPII phosphorylated at CTD Serine 2 (pSer2) and 5 (pSer5), and total RNAPII. Total proteins were extracted 72 h after siRNA transfection. Beta Actin (βAct) serves as a loading control. Marker molecular weights are on the left of the gels in kDa. (**B**) pSer2 and pSer5 signals were quantified and graphed after normalization using the corresponding total RNAPII signals. siCt values are set to 1. Bars and error bars are means and SDs from three biological replicates. $P1 = 0.0071$; $P2 = 0.0077$ (Student's *t*-test). Source data are available online for this figure.

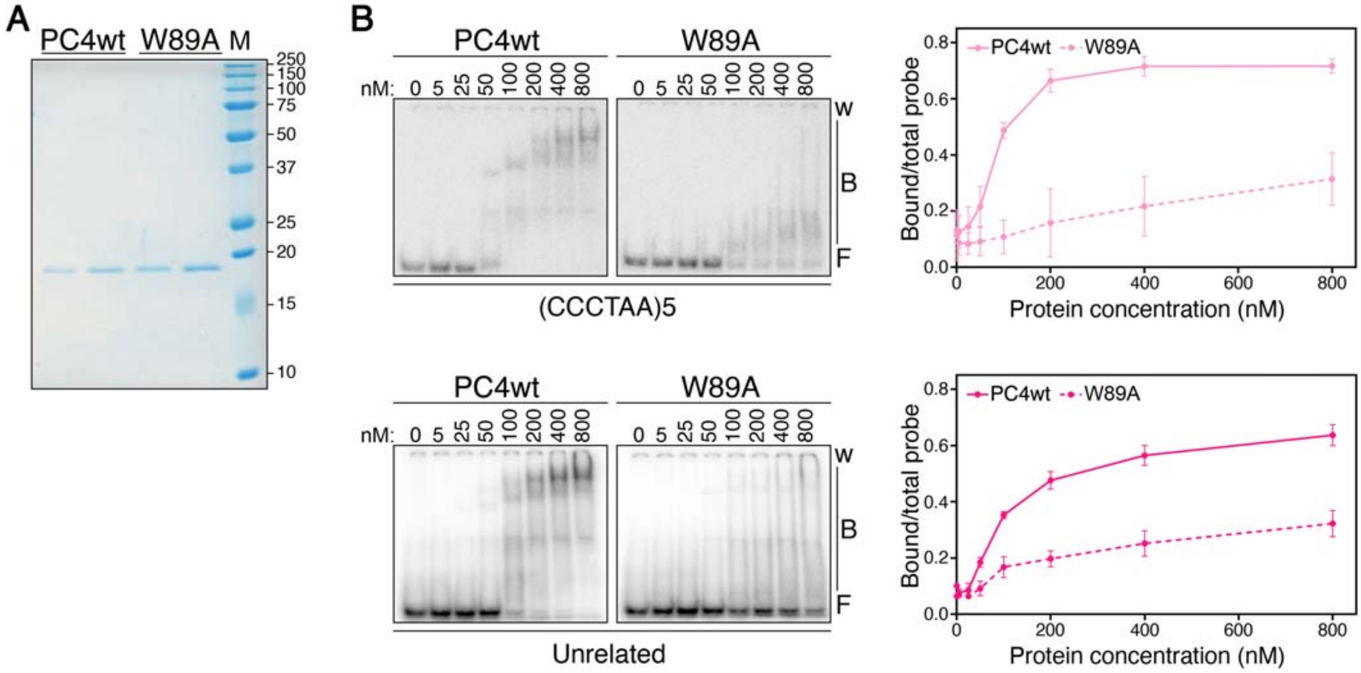

Figure EV5. The ssDNA binding-deficient mutant W89A binds to ssDNA substrates.

(**A**) 150 and 300 ng of PC4wt and 250 and 500 ng of W89A recombinant proteins were size-fractionated by SDS-PAGE and stained with BlueSafe reagent. Marker (M) molecular weights are on the right in kDa. (**B**) Electrophoretic mobility shift assay was performed with recombinant PC4wt and W89A proteins and the indicated ssDNA oligonucleotides. w: wells; B: bound probe; F: free probe. The graphs show quantifications of bound oligonucleotides graphed as a fraction of the total signal within each lane. Data points and error bars are means and SDs from three biological replicates.

 © The Author(s)
